# Population Health Impact and Cost-Effectiveness of Tuberculosis Diagnosis with Xpert MTB/RIF: A Dynamic Simulation and Economic Evaluation

**DOI:** 10.1371/journal.pmed.1001347

**Published:** 2012-11-20

**Authors:** Nicolas A. Menzies, Ted Cohen, Hsien-Ho Lin, Megan Murray, Joshua A. Salomon

**Affiliations:** 1 Center for Health Decision Sciences, Harvard School of Public Health, Boston, Massachusetts, United States of America; 2 Institute for Technology Assessment, Massachusetts General Hospital, Boston, Massachusetts, United States of America; 3 Department of Epidemiology, Harvard School of Public Health, Boston, Massachusetts, United States of America; 4 Division of Global Health Equity, Brigham and Women's Hospital, Boston, Massachusetts, United States of America; 5 Graduate Institute of Epidemiology and Preventive Medicine, National Taiwan University, Taipei, Taiwan; 6 Department of Global Health and Population, Harvard School of Public Health, Boston, Massachusetts, United States of America; Boston University, United States of America

## Abstract

**Background:**

The Xpert MTB/RIF test enables rapid detection of tuberculosis (TB) and rifampicin resistance. The World Health Organization recommends Xpert for initial diagnosis in individuals suspected of having multidrug-resistant TB (MDR-TB) or HIV-associated TB, and many countries are moving quickly toward adopting Xpert. As roll-out proceeds, it is essential to understand the potential health impact and cost-effectiveness of diagnostic strategies based on Xpert.

**Methods and Findings:**

We evaluated potential health and economic consequences of implementing Xpert in five southern African countries—Botswana, Lesotho, Namibia, South Africa, and Swaziland—where drug resistance and TB-HIV coinfection are prevalent. Using a calibrated, dynamic mathematical model, we compared the status quo diagnostic algorithm, emphasizing sputum smear, against an algorithm incorporating Xpert for initial diagnosis. Results were projected over 10- and 20-y time periods starting from 2012. Compared to status quo, implementation of Xpert would avert 132,000 (95% CI: 55,000–284,000) TB cases and 182,000 (97,000–302,000) TB deaths in southern Africa over the 10 y following introduction, and would reduce prevalence by 28% (14%–40%) by 2022, with more modest reductions in incidence. Health system costs are projected to increase substantially with Xpert, by US$460 million (294–699 million) over 10 y. Antiretroviral therapy for HIV represents a substantial fraction of these additional costs, because of improved survival in TB/HIV-infected populations through better TB case-finding and treatment. Costs for treating MDR-TB are also expected to rise significantly with Xpert scale-up. Relative to status quo, Xpert has an estimated cost-effectiveness of US$959 (633–1,485) per disability-adjusted life-year averted over 10 y. Across countries, cost-effectiveness ratios ranged from US$792 (482–1,785) in Swaziland to US$1,257 (767–2,276) in Botswana. Assessing outcomes over a 10-y period focuses on the near-term consequences of Xpert adoption, but the cost-effectiveness results are conservative, with cost-effectiveness ratios assessed over a 20-y time horizon approximately 20% lower than the 10-y values.

**Conclusions:**

Introduction of Xpert could substantially change TB morbidity and mortality through improved case-finding and treatment, with more limited impact on long-term transmission dynamics. Despite extant uncertainty about TB natural history and intervention impact in southern Africa, adoption of Xpert evidently offers reasonable value for its cost, based on conventional benchmarks for cost-effectiveness. However, the additional financial burden would be substantial, including significant increases in costs for treating HIV and MDR-TB. Given the fundamental influence of HIV on TB dynamics and intervention costs, care should be taken when interpreting the results of this analysis outside of settings with high HIV prevalence.

*Please see later in the article for the Editors' Summary*

## Abstract 2

Nicolas Menzies and colleagues investigate the potential impact and cost-effectiveness of implementing Xpert MTB/RIF for diagnosing tuberculosis in five southern African countries.

## Editors' Summary

**Background:**

In 2010, about 9 million people developed tuberculosis (TB)—a contagious bacterial disease that usually infects the lungs—and about 1.5 million people died from the disease. Most of these deaths were in low- and middle-income countries, and a quarter were in HIV-positive individuals, who are particularly susceptible to TB. *Mycobacterium tuberculosis*, the bacterium that causes TB, is spread in airborne droplets when people with active disease cough or sneeze. The characteristic symptoms of TB are a persistent cough, weight loss, fever, and night sweats. Diagnostic tests for TB include sputum smear analysis (microscopic examination of mucus coughed up from the lungs for the presence of *M. tuberculosis*) and mycobacterial liquid culture (growth of *M. tuberculosis* from sputum and determination of its drug sensitivity). TB can be cured by taking several antibiotics daily for at least six months, although the recent emergence of multidrug-resistant TB (MDR-TB) is making the disease increasingly hard to treat.

**Why Was This Study Done?:**

To reduce the global TB burden, active disease must be diagnosed quickly and accurately. In most high-burden settings, however, TB diagnosis relies on sputum smear analysis, which fails to identify some people (especially HIV-infected individuals) who have TB. Mycobacterial culture correctly identifies more infected people but is slow and costly, and many high-burden settings lack the infrastructure for high-volume culture diagnosis of TB. Faced with these diagnostic inadequacies, the World Health Organization (WHO) recently recommended the use of Xpert MTB/RIF for initial diagnosis in patients suspected of having MDR-TB or HIV-associated TB. This new, automated DNA test detects *M. tuberculosis* and DNA differences that make the bacteria resistant to the drug rifampicin (an indicator of MDR-TB) within two hours. Many countries are moving toward adopting Xpert for TB diagnosis, so it is essential to understand the population health impact and cost-effectiveness of diagnostic strategies based on this test. Here, the researchers use a calibrated, dynamic mathematical model of TB to investigate the consequences of Xpert MTB/RIF implementation in five southern African countries where both TB-HIV coinfection and MDR-TB are common.

**What Did the Researchers Do and Find?:**

The researchers used their mathematical model, which simulates the movement of individuals through different stages of TB infection, to investigate the potential health and economic consequences of implementing Xpert for initial TB diagnosis in Botswana, Lesotho, Namibia, South Africa, and Swaziland. In the modeled scenarios, compared to an diagnostic approach based on sputum smear (the “status quo”), implementation of Xpert averted an estimated 132,000 TB cases and 182,000 TB deaths in southern Africa over the ten years following its introduction, reduced the proportion of the population with TB by 28%, and increased health service costs by US$460 million. Much of this cost increase reflected increased antiretroviral therapy costs for TB/HIV-infected individuals who survived TB infection because of better case-finding and treatment. Finally, relative to the status quo, over ten years, Xpert implementation in southern Africa cost US$959 for every DALY (disability-adjusted life-year) averted. Cost-effectiveness ratios in individual countries ranged from US$792 per DALY averted in Swaziland to US$1,257 per DALY averted in Botswana.

**What Do These Findings Mean?:**

These findings suggest that Xpert implementation in southern Africa could substantially reduce TB illness and death through improved case-finding and treatment, but that the impact of Xpert on long-term transmission dynamics may be more limited. Although the additional financial burden associated with Xpert roll-out is likely to be substantial, these findings suggest that using Xpert for TB diagnosis offers reasonable value given its cost. WHO considers any intervention with a cost-effectiveness ratio less than the per-capita gross domestic product (GDP) highly cost-effective—in 2010, the per-capita GDP ranged from US$7,000 in South Africa and Botswana to US$982 in Lesotho.

These findings may not be generalizable to regions with different HIV infection rates, and their accuracy is likely to be affected by the quality of the data fed into the mathematical model and by the structure of the model. Thus, it is essential that the impact of Xpert-based TB diagnosis be carefully evaluated as the approach is rolled out, and that the information generated by these evaluations be used to improve the accuracy of model-based estimates of the long-term effects of this new strategy for TB diagnosis.

**Additional Information:**

Please access these websites via the online version of this summary at http://dx.doi.org/10.1371/journal.pmed.1001347.

- WHO provides information (in several languages) on all aspects of tuberculosis, including general information on tuberculosis diagnostics and specific information on the roll-out of the Xpert MTB/RIF test; further information about WHO's endorsement of Xpert MTB/RIF is included in a recent Strategic and Technical Advisory Group for Tuberculosis report; WHO also provides information about tuberculosis and HIV
- The Stop TB Partnership is working towards tuberculosis elimination; patient stories about TB-HIV coinfection are available
- The US Centers for Disease Control and Prevention has information about tuberculosis, and about TB diagnosis
- The US National Institute of Allergy and Infectious Diseases also has detailed information on all aspects of tuberculosis
- The Tuberculosis Survival Project, which aims to raise awareness of tuberculosis and provide support for people with tuberculosis, provides personal stories about treatment for tuberculosis; the Tuberculosis Vaccine Initiative also provides personal stories about dealing with tuberculosis
- MedlinePlus has links to further information about tuberculosis (in English and Spanish)

## Introduction

Tuberculosis (TB) remains a leading cause of global mortality and morbidity, with an estimated 9 million new TB cases and 1.5 million TB-related deaths in 2010 [1]. Although significant advances have been made in improving TB outcomes under the DOTS approach championed by the World Health Organization (WHO) and its partners in the Stop TB Partnership [2], continued progress is threatened by the inadequacy of existing diagnostic tools [3]. In most high-burden settings, TB diagnosis relies principally on sputum smear microscopy, which has limited sensitivity, especially among HIV-infected patients [4]–[6]. Traditional culture-based diagnosis and evaluation of drug sensitivity is relatively costly and slow [7],[8], and many resource-limited settings lack the laboratory capacity to perform culture and sensitivity testing at high volume [9],[10]. Lack of prompt diagnosis and appropriate treatment of TB increases the risks of transmission, drug resistance, and case fatality [11]–[13].

Recently, the Xpert MTB/RIF automated DNA test has been shown to provide rapid and sensitive detection of TB and rifampicin (RIF) resistance [14]–[17]. The Xpert test uses a cartridge-based system that integrates sample processing and real-time PCR, accommodates use by relatively unskilled healthcare workers, and provides results in <2 h [15],[18]. In a large multicenter evaluation and subsequent implementation study, a single Xpert MTB/RIF test was found to identify >98% of patients with smear-positive TB and >70% of patients with smear-negative TB [14],[15]. Sensitivity and specificity for RIF resistance were above 94% and 98%, respectively. More recent analyses have suggested that Xpert can greatly reduce the delay until treatment initiation for individuals with active TB [19].

In December 2010, WHO recommended that Xpert be used for initial diagnosis in patients suspected of having multidrug-resistant TB (MDR-TB) or HIV-associated TB disease [20]. By the end of May 2012, 66 of 145 countries eligible to purchase Xpert equipment at reduced prices had already done so [21]. A volume-dependent price mechanism is being used for purchase of test cartridges [22], such that by August 2012 the ex-works price of Xpert cartridges had dropped to less than US$10 for eligible countries [23]. Whereas the global TB control community has moved quickly to embrace the new technology, several studies and commentaries have sounded important notes of caution concerning the cost of the technology, the demand it will place on existing infrastructure, and the challenge of addressing false positive indications of RIF resistance [24]–[30]. As implementation advances, evidence on the epidemiologic impact and cost-effectiveness of Xpert is urgently needed, particularly as the consequences of Xpert introduction may vary across epidemiologic settings and may depend on the specific diagnostic algorithms that are considered [31],[32].

In this study we used a calibrated, dynamic mathematical model of TB to quantify the potential health and economic consequences of introducing Xpert in five southern African countries characterized by high prevalence of HIV infection and extant multidrug resistance. Comparing a diagnostic strategy based on Xpert to the status quo, we predicted changes in TB incidence, prevalence, mortality, and drug resistance; estimated health system costs; and assessed the incremental cost-effectiveness of Xpert adoption.

## Methods

### Overview

We evaluated the population health outcomes and health system costs associated with two alternative strategies for diagnosing TB, the first based on current diagnostic algorithms and the second based on implementing Xpert in accordance with current WHO recommendations. Comparisons between these two strategies were made using a calibrated mathematical model of TB, reflecting key features of TB transmission dynamics and natural history, interactions with HIV infection, and patterns and trends in TB control interventions and treatment for HIV/AIDS. Model simulations were undertaken for five southern African countries: Botswana, Lesotho, Namibia, South Africa, and Swaziland. We assessed changes in epidemiological outcomes and health system costs over 10-y and 20-y time horizons, as well as the incremental cost-effectiveness ratio (ICER) of the Xpert strategy compared to the current algorithm.

### Diagnostic Strategies

A “status quo scenario” was created to represent the current diagnostic approach. Under this approach, all patients with suspected TB receive an initial sputum smear, and those diagnosed as smear-positive are directed to treatment. Sputum culture is indicated for patients with suspected TB who test smear-negative but who have a history of TB treatment or in whom there is a strong suspicion of TB. Drug sensitivity testing (DST) is indicated for treatment-experienced patients diagnosed with TB. Those who receive DST are initiated on a treatment regimen appropriate to their drug resistance profile, while those who do not receive DST are initiated on the standard first-line regimen. In the main analysis we assumed that the coverage of culture testing would be 20% (range 10%–30%) among smear-negative, treatment-naïve patients, and 80% (range 70%–90%) among smear-negative, treatment-experienced patients. We assumed further that 80% (range 70%–90%) of treatment-experienced patients diagnosed with TB would go on to receive DST. Given limited empirical data on country-specific coverage of culture and DST, these values were all varied across wide ranges in sensitivity analyses.

An “Xpert scenario” was constructed based on the diagnostic algorithms suggested for high HIV prevalence settings in the May 2011 WHO recommendations for Xpert implementation [33]. These recommendations suggest the use of Xpert as an initial diagnostic for all individuals of HIV-positive or unknown status. Given the high prevalence of HIV among patients with suspected TB in southern Africa and the low number of individuals with a recent HIV test result [34], we modeled an algorithm in which Xpert was used as the initial diagnostic for all patients with suspected TB. According to this algorithm, such patients are first tested with a single Xpert assay, and no sputum smear is performed. Those testing TB-positive but negative for RIF resistance are initiated on a standard first-line regimen. Those testing positive for RIF resistance go on to receive DST. If the DST result indicates drug resistance, the individual is treated with a drug regimen tailored to the observed resistance profile. Under this scenario we assumed that scale-up to full coverage of Xpert within the national TB program would occur over the 3-y period starting in 2012. A diagram of the two alternative diagnostic algorithms is shown in Figure S1.

### Modeling Approach

**Figure 1:**
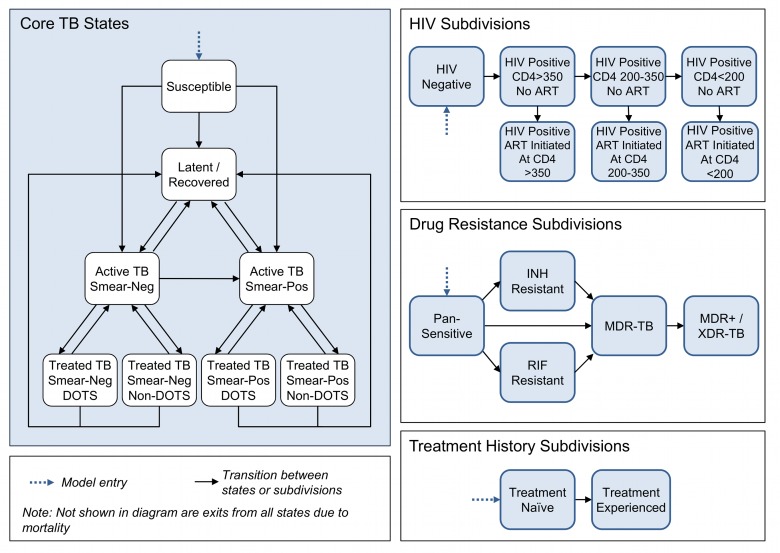
Model states, subdivisions, and transitions.

We developed a dynamic compartmental model of TB following the conventions of earlier models [35]–[41], with additional detail to accommodate evaluation of alternative diagnostic strategies. The model structure (Figure 1) is defined by a set of core TB states, and these states are further subdivided to account for (1) aspects of HIV infection, progression, and treatment relevant to TB epidemiology; (2) multiple circulating TB strains, with different drug resistance profiles; and (3) tracking of TB treatment history.

#### Core TB states

The core TB model simulates the movements of individuals between states that capture important features of TB transmission, natural history, and treatment. Individuals enter the model in the susceptible state, where they face a risk of TB infection. The risk of infection is modeled as a time-dependent variable that reflects contact rates between infected and uninfected individuals, and transmission probabilities that allow for varying infectivity across different categories of active disease. Upon infection, individuals progress either directly to active disease or to latent infection. Individuals with latent infection may subsequently progress to active TB or be superinfected by a different TB strain. Active disease is categorized as smear-positive or smear-negative. Smear-negative cases may progress to smear-positive, and all individuals with active disease may spontaneously self-cure, which returns them to the latent/recovered state. An individual with active disease can be diagnosed as a TB case, according to the characteristics of the diagnostic algorithm, and initiated on treatment (as described in detail below). All individuals in the model are subject to a background mortality rate and to TB-related mortality specific to each active disease state.

#### HIV subdivisions

HIV coinfection can alter the natural history of TB, with HIV-infected individuals having a higher probability of primary progressive TB upon initial infection [42],[43], a higher rate of breakdown from latent infection to active TB [44], a lower probability of smear-positivity amongst those with active disease [4]–[6], and higher mortality rates [4],[45],[46]. The HIV sub-model draws on model structure and key parameters from an array of published HIV models [47]–[50]. Seven HIV subdivisions were created, defined by CD4 cell count (>350 cells/µl, 200–350 cells/µl, and <200 cells/µl) and by whether or not an individual is receiving antiretroviral therapy (ART). HIV incidence is modeled as a transition from the HIV-negative category to the HIV-positive, CD4 count >350 cells/µl category, with time-varying incidence rates defined as exogenous model parameters. HIV-positive individuals not on ART progress over time to subdivisions with lower CD4 counts. Untreated individuals transition onto ART at rates specific to their CD4 category. These rates are allowed to vary over time to capture changing eligibility criteria and coverage of testing and referral. HIV-related mortality occurs at rates specific to each subdivision.

#### Drug resistance subdivisions

Model states are further subdivided to account for differences in drug resistance among circulating TB strains, including (1) pan-sensitive TB, (2) isoniazid (INH) mono-resistant TB, (3) RIF mono-resistant TB, (4) TB resistant to both INH and RIF (MDR-TB), and (5) TB resistant to INH and RIF plus one or more second-line drugs (MDR+/XDR-TB). An individual in the susceptible state who is newly infected with TB transitions to the subdivision of the infecting strain. An individual with latent TB who is superinfected by a different strain transitions to the subdivision of the superinfecting strain. Individuals may also develop acquired drug resistance as a result of TB treatment, transitioning to subdivisions with broader resistance profiles.

#### Treatment history subdivisions

A final subdivision of model states distinguishes treatment-naïve from treatment-experienced individuals, as diagnostic algorithms may dictate different confirmatory tests depending on an individual's history of prior treatment. Individuals enter the model in the treatment-naïve subdivision, and all individuals exiting their first course of TB treatment (through default, failure, or cure) transition to the treatment-experienced subdivision.

The model is implemented as a series of difference equations with a monthly time step. A full description of model structure and equations is given in Text S1.

### TB Diagnosis and Treatment

The model allows for TB diagnosis and treatment through the national TB DOTS program, or through non-DOTS providers functioning outside the national program. Uptake into treatment programs requires that individuals (1) present to a health facility and are identified as patients with suspected TB, (2) are diagnosed as active cases, and (3) are initiated on regimens determined by their background characteristics and information on drug sensitivity, if available. The model accounts for differences in test performance and information provided by each diagnostic algorithm, and for attrition between diagnosis and treatment, which varies depending on the delay to test results [51]. Individuals with false negative diagnoses for active TB will remain in the pool of undiagnosed active TB cases, with the possibility of presenting for diagnosis again. Individuals without active TB who attend with TB symptoms and are incorrectly diagnosed with active TB are assumed to undergo TB treatment, incurring costs but no positive or negative health effects. Algorithms for diagnosis and treatment in non-DOTS programs are assumed to be the same in both the status quo and Xpert scenarios, i.e., independent of the choice of diagnostic algorithm in the national DOTS program.

Individuals on TB treatment may successfully complete treatment, fail, default (become lost to follow-up), or die. Those who successfully complete treatment return to the latent/recovered state. A percentage of individuals failing therapy are identified as failures by the treatment program and reinitiate treatment, while all others return to active disease. Individuals who fail or default from treatment may acquire resistance to the drugs they have received. The model allows individuals with pan-sensitive TB to develop mono-INH-resistant TB, mono-RIF-resistant TB, or MDR-TB directly. Individuals with mono-INH- or mono-RIF-resistant TB can develop MDR-TB, and individuals with MDR-TB can develop MDR+/XDR-TB, with the rates of acquiring drug resistance dependent on a patient's TB drug regimen and current drug resistance profile (see details in Text S1).

### Impact of Diagnostic Algorithms on TB Epidemiology

Any change in diagnostic algorithm is assumed to impact TB epidemiology through two channels. The first major effect is via changes in the overall sensitivity and specificity of TB diagnosis. For the population with undiagnosed active TB, an improvement in diagnostic sensitivity results in improved case detection and reduced delay to treatment initiation and, consequently, increases survival and decreases the duration of infectiousness. The second major effect is via changes in the distribution of regimens received by newly diagnosed TB cases. Drug-resistant TB cases identified by an algorithm with better sensitivity for diagnosing resistance have a higher probability of being initiated on a more effective treatment regimen, which in turn improves cure rates, increases survival, and reduces the probability that a patient will return to an infectious state.

### Estimation Approach

We used a Bayesian estimation approach developed by Raftery and colleagues [52],[53] and recently adopted by the Joint United Nations Programme on HIV/AIDS for HIV epidemic projections [54]–[56]. This approach provides a method for calibrating complex nonlinear models to reported data on disease burden, and for characterizing uncertainty in analysis results using Bayesian posterior intervals and similar metrics. These features are particularly important for our analysis, given the substantial uncertainty around many of the parameters describing TB epidemiology. We used this approach to calibrate the model to independent WHO estimates of TB incidence and prevalence in each of the five countries [57], and to data from drug resistance surveys available for all countries except Namibia [58]. The analysis was implemented using a sampling/importance resampling algorithm [52],[55],[59]. First, a large number of parameter sets were drawn from the joint prior distribution of the input parameters. For each of these parameter sets the model was run and a likelihood statistic calculated by comparing model outcomes to the corresponding calibration data. The likelihood for each parameter set was then used as the probability weight in a second-stage resample of the parameter sets, which yielded draws representing the posterior parameter distribution, reflecting the information available on both model inputs and calibration data. The results of this simulation are similar to those produced by traditional Monte Carlo simulation and probabilistic sensitivity analyses, with the additional benefit of being constrained to be consistent with independent estimates on TB outcomes for each country. For each modeled outcome, uncertainty intervals were calculated by taking the 2.5th and 97.5th percentiles of the distribution for this outcome generated by the resampled parameter sets, and the point estimate was calculated by taking the arithmetic mean of this distribution (see Text S1 for further detail).

### Model Parameter Values

We parameterized the model using historical demographic and epidemiologic data available for each country. Parameter values relating to population demographics were derived from United Nations Population Division estimates and projections. Parameter values relating to TB transmission dynamics were chosen to be consistent with data and assumptions used in earlier TB models [35]–[41]. Parameter values relating to TB program coverage and treatment outcomes were derived from published reporting data [57]. Key parameter values relating to TB diagnosis and treatment are summarized in Table 1. Estimates for HIV incidence and ART access between 1983 and 2010 were derived from unpublished data provided by the Joint United Nations Programme on HIV/AIDS. Future ART access was assumed to increase from current levels to the WHO universal access target of 80% coverage [60] over the course of 10 y. For Botswana, which was providing ART to an estimated 83% of those in need by 2009, coverage was maintained at current levels. ART eligibility was initially limited to individuals with CD4 count <200 cells/µl and then extended to include those with a CD4 count in the range 200–350 cells/µl from 2010 onward, consistent with the expansion of ART eligibility in WHO HIV treatment guidelines [61],[62]. A full description of all parameters in the model is provided in Text S1.

**Table 1:** Selected model parameter values and ranges.

Description	Base-Case Value	Range	Source
**Sensitivity of sputum smear microscopy**			Assumed^a^
Smear-negative TB	0.0	—	
Smear-positive TB	1.0	—	
**Specificity of sputum smear microscopy**	0.974	(0.965–0.982)	[82]
**Sensitivity of sputum culture**	1.0	—	Assumed^b^
**Specificity of sputum culture**	0.984	(0.978–0.989)	[83]
**Sensitivity of Xpert for TB**			[14]
Smear-negative TB	0.725	(0.655–0.788)	
Smear-positive TB	0.982	(0.969–0.991)	
**Specificity of Xpert for TB**	0.992	(0.982–0.997)	[14]
**Sensitivity of Xpert for RIF resistance**	0.976	(0.946–0.992)	[14]
**Specificity of Xpert for RIF resistance**	0.981	(0.966–0.990)	[14]
**Probability of sputum culture following a negative sputum smear (status quo algorithm)**			[84]
Treatment-naïve patients	0.20	(0.11–0.31)	
Treatment-experienced patients	0.80	(0.69–0.89)	
**Probability of DST following a positive TB diagnosis (status quo algorithm)**			[84]
Treatment-naïve patients	0.00	—	
Treatment-experienced patients	0.80	(0.69–0.89)	
**Probability of loss to follow-up between initial presentation and treatment initiation**			[51]
With prompt diagnosis (smear, Xpert)	0.15	(0.09–0.24)	
With delayed diagnosis (culture, DST)	0.25	(0.14–0.39)	
**Background mortality rate (ages 15+ y)**	Time-varying	—	WHO unpublished data
**Excess mortality rate for active TB**			[38]
Smear-negative	0.21	(0.18–0.25)	
Smear-positive	0.30	(0.21–0.41)	
**Excess mortality rate for HIV**			[85]–[90]
CD4 >350 cells/µl, no ART	0. 008	(0.005–0.012)	
CD4 200–350 cells/µl, no ART	0.030	(0.018–0.048)	
CD4<200 cells/µl, no ART	0.230	(0.136–0.366)	
On ART initiated at CD4 >350 cells/µl	0.008	(0.005–0.012)	
On ART initiated at CD4 200–350 cells/µl	0.023	(0.014–0.037)	
On ART initiated at CD4<200 cells/µl	0.050	(0.031–0.076)	
**Excess mortality rate for advanced HIV (CD4<200 cells/µl) and active TB without ART**	0.80	(0.472–1.272)	[45],[46]
**Per-test cost of Xpert**	$20, $30, $40	Fixed^c^	[33],[64],[65]
**Per-test cost of smear diagnosis**			[51],[91]–[97]
Botswana	$6.13	(4.18–8.68)	
Lesotho	$3.31	(2.26–4.68)	
Namibia	$5.31	(3.63–7.51)	
South Africa	$5.94	(4.06–8.39)	
Swaziland	$4.24	(2.90–5.99)	
**Per-test cost of culture**			[51],[91],[93],[94],[97]
Botswana	$15.83	(13.07–18.99)	
Lesotho	$8.56	(7.07–10.27)	
Namibia	$13.72	(11.33–16.46)	
South Africa	$15.33	(12.66–18.39)	
Swaziland	$10.94	(9.04–13.13)	
**Per-test cost of chest X-ray**			[91],[96],[98]
Botswana	$16.69	(11.35–23.70)	
Lesotho	$9.03	(6.14–12.81)	
Namibia	$14.46	(9.83–20.52)	
South Africa	$16.16	(10.99–22.94)	
Swaziland	$11.54	(7.85–16.38)	
**Per-test cost of DST**			[7],[99]
Botswana	$81.97	(61.44–107.17)	
Lesotho	$44.32	(33.22–57.94)	
Namibia	$71.02	(53.24–92.85)	
South Africa	$79.37	(59.50–103.77)	
Swaziland	$56.65	(42.47–74.07)	
**Cost of outpatient diagnostic visit**			[100]
Botswana	$10.32	(6.09–16.40)	
Lesotho	$2.94	(1.73–4.67)	
Namibia	$7.99	(4.71–12.70)	
South Africa	$10.30	(6.08–16.39)	
Swaziland	$6.21	(3.66–9.87)	
**Cost of outpatient treatment visit**			[100]
Botswana	$6.85	(4.04–10.89)	
Lesotho	$1.95	(1.15–3.10)	
Namibia	$5.31	(3.13–8.44)	
South Africa	$6.85	(4.04–10.89)	
Swaziland	$4.13	(2.44–6.57)	
**Cost of inpatient care, per day**			[100]
Botswana	$38.99	(23.00–61.99)	
Lesotho	$8.78	(5.18–13.96)	
Namibia	$28.76	(16.97–45.73)	
South Africa	$39.38	(23.23–62.61)	
Swaziland	$21.91	(12.93–34.84)	
**Monthly TB regimen cost**			[63]
First-line	$5.86	(3.46–9.32)	
Mono-INH resistant	$18.02	(10.63–28.65)	
Mono-RIF resistant	$33.91	(20.01–53.92)	
MDR-TB	$119.37	(70.43–189.79)	
MDR+/XDR-TB	$179.06	(105.64–284.70)	
**Monthly cost of ART**			[63],[101]–[105]
Botswana	$104.97	(84–80–128.48)	
Lesotho	$69.63	(57.22–83.92)	
Namibia	$94.68	(76.78–115.52)	
South Africa	$102.53	(82.90–125.40)	
Swaziland	$81.20	(66.25–98.52)	
**Disability weights**			[66],[67]
Active TB	0.271	(0.151–0.422)	
HIV-positive, CD4 >350 cells/µl, no ART	0.135	(0.078–0.213)	
HIV-positive, CD4 200–350 cells/µl, no ART	0.320	(0.176–0.496)	
HIV-positive, CD4<200 cells/µl, no ART	0.505	(0.252–0.757)	
HIV-positive, on ART initiated at CD4 >350 cells/µl	0.135	(0.078–0.213)	
HIV-positive, on ART initiated at CD4 200–350 cells/µl	0.151	(0.087–0.238)	
HIV-positive, on ART initiated at CD4<200 cells/µl	0.167	(0.096–0.262)	

All costs are given in 2011 US dollars.

a As smear status is tracked in the model, the sensitivity of sputum smear for individuals classed as smear-negative and smear-positive is 0% and 100% (respectively) by construction.

b As sputum culture is the gold standard for TB detection, the sensitivity is assumed to be 100%.

c As the per-test cost of Xpert is of key interest to policy-makers (and potentially subject to price negotiation), the results of the analyses are presented for three separate values for the Xpert cost.

### Measurement of Resource Use and Costs

Costs were assessed from a health system perspective and expressed in 2011 US dollars. Costs reflected resources used to deliver TB diagnosis and treatment, as provided by both public and private providers, and resources used in providing ART to HIV-infected individuals. An ingredients approach to costing was used, by which the total cost to provide a particular diagnostic procedure or a course of treatment was calculated by estimating the number of units of each specific type of resource input needed to deliver the service, multiplying each quantity by the corresponding unit cost of that resource input, and summing across all inputs.

Average costs for each type of service are shown in Table 1. Cost estimates extrapolated from the literature were adjusted for inflation, currency conversions, and price levels, where relevant. Treatment costs for TB and HIV included drugs, clinic visits, and monitoring tests, including regular smear examinations during TB treatment. Drug costs were derived from the WHO price reporting mechanism [63]. Costs for laboratory tests (excluding Xpert) were derived from the literature. Numbers of treatment monitoring visits and laboratory tests followed a previous global analysis [35]. For Xpert, estimates in WHO implementation guidelines [33] suggest an economic cost of US$25–US$35 per test in southern Africa (including consumables, equipment, personnel, transport, facilities, and managerial overheads). This range of estimates is consistent with the results from a cost analysis conducted for the South African national program, which found a cost range of US$25–US$33 [64], as well as an analysis of potential implementation strategies that reported costs of US$27 per patient with suspected TB for placement of equipment at central laboratories and US$39 for placement of equipment at point of care [65]. Costs of Xpert may continue to change as volume increases, through reductions in the prices of equipment and consumables [22],[23], economies of scale, and accumulated implementation experience; we therefore conducted analyses using Xpert per-test costs of US$20, US$30, and US$40.

### Outcomes

We estimated trends in population-level epidemiological outcomes including TB prevalence, incidence, mortality, and resistance to anti-TB drugs, prior to Xpert introduction in 2012, and over the subsequent 20-y period. Summary outcome measures computed based on population survivorship in the model included life-years and disability-adjusted life-years (DALYs), the latter incorporating disability weights from the Global Burden of Disease study [66],[67]. We evaluated the cost-effectiveness of introducing Xpert in terms of the ICER, expressed as the difference in total costs between the Xpert and status quo scenarios, divided by the difference in life-years or DALYs between the two scenarios. Cost-effectiveness ratios were computed over both 10-y and 20-y time horizons following Xpert introduction, in each case based only on the costs and health outcomes accrued during that period. Costs and health benefits were discounted at an annual rate of 3% [68],[69]. Following standard benchmarks proposed in international work on cost-effectiveness, we compared the ICER to thresholds for cost-effectiveness defined in reference to the annual gross domestic product (GDP) per capita in each country. Interventions are considered to be highly cost-effective when they have ICERs that fall below the annual per-capita GDP, and are regarded as being potentially cost-effective if they have ICERs between one and three times annual per-capita GDP [70].

### Sensitivity Analysis

The sensitivity of the model to changes in individual parameters was investigated through traditional one-way sensitivity analyses as well as by computing partial rank correlation coefficients across the set of simulation results produced by the Bayesian uncertainty analysis [38],[71],[72]. For the one-way sensitivity analyses, we computed the change in the ICER (calculated over a 10-y time horizon) that would occur when we changed one parameter value by ±1 standard deviation from its posterior mean value while holding all other parameter values at their posterior means. We also conducted an array of additional sensitivity analyses that varied assumptions regarding the diagnostic algorithms being compared, the use of inpatient care as part of MDR-TB treatment, future ART coverage decisions, and trends in antiretroviral drug prices.

Finally, we conducted a probabilistic sensitivity analysis to assess the uncertainty around the optimal choice of diagnostic strategy resulting from the joint effects of uncertainty around all input parameters simultaneously, and these results are presented as posterior intervals around key model outcomes and as cost-effectiveness acceptability curves.

## Results

### Epidemiological Projections under the Current Diagnostic Algorithm

**Figure 2:**
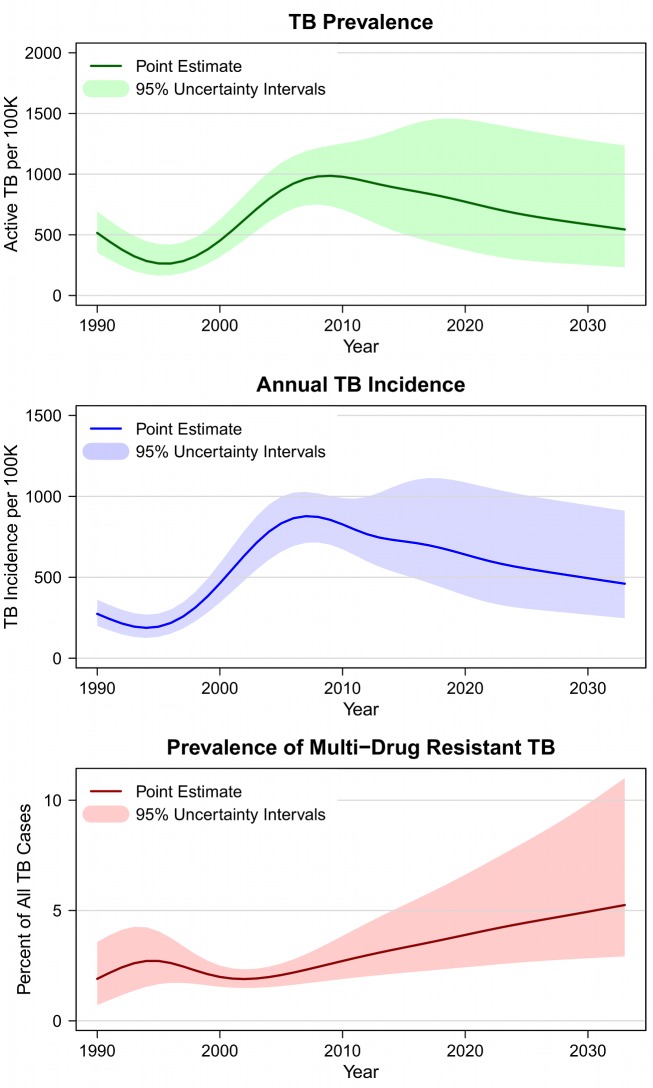
Estimated and projected TB prevalence, TB incidence, and multidrug-resistant TB prevalence in southern Africa under status quo diagnostic algorithm, 1990–2032.

Figure 2 shows estimates and projections for TB prevalence and incidence in the southern Africa region from 1990 through the end of 2032, under the assumption that the current (status quo) diagnostic algorithm is used over the whole period. The results for individual countries followed the general trend seen in the regional results, with historical declines in TB prevalence and incidence reversed over the period 1995–2010 as a consequence of concurrent HIV epidemics. The magnitude of the TB epidemic differed across individual countries, with Lesotho having the lowest prevalence and incidence and Swaziland the highest.

### Performance of Diagnostic Algorithms

Based on our model simulations, the positive predictive value for Xpert diagnosis of active TB, at full coverage by 2014, would be 96.9% (95% CI: 93.4–98.7), compared to 88.4% (81.5–93.1) for the status quo algorithm. The negative predictive values for Xpert and the status quo would be 93.9% (88.8–97.2) and 79.3% (67.6–87.9), respectively. We estimate the positive predictive value for the diagnosis of RIF resistance by Xpert to be 67.3% (51.3–82.0) and the negative predictive value 99.9% (99.8–100.0). The relatively low positive predictive value indicates that Xpert is expected to produce a number of false positive diagnoses of RIF resistance, with relatively modest implications for treatment outcomes, as we assume that a subsequent DST is required before individuals receive an MDR-TB diagnosis. Under the Xpert algorithm, 5.8 (95% CI: 3.8–9.2) patients are tested for TB for each active case starting treatment, compared to 7.5 (4.9–12.1) under the status quo, a consequence of improved sensitivity in the Xpert algorithm. The average duration of infectiousness is 9.9 mo (95% CI: 6.7–14.0) under the Xpert algorithm compared to 12.8 mo (9.6–14.0) under the status quo. The benefit of the reduced duration of infectiousness is primarily accrued among individuals with smear-negative TB, for whom the duration of infectiousness is reduced from 19.3 mo (13.8–24.6) under the status quo to 12.1 mo (7.8–18.0) under the Xpert scenario. Results for those with smear-positive disease are comparable under both scenarios. Treatment effectiveness (the probability of cure for individuals starting treatment) rises only marginally under the Xpert scenario, with the probability of cure 2.7 (95% CI: 1.6–4.4) percentage points higher than in the status quo scenario. Table 2 presents estimates for the average cost per programmatic outcome for the status quo and Xpert strategies, summed over the first 10 y of Xpert implementation (2012–2022). These results show that adopting the Xpert algorithm increases the cost of achieving various diagnostic and treatment outcomes.

**Table 2:** Average programmatic outcomes and costs over 10 y following choice of strategy.

Outcome	Status Quo Strategy	Xpert Strategy
**Programmatic measures for DOTS diagnosis**		
Average annual DOTS diagnosis costs	$27 million (15–46 million)	$37 million (21–61 million)
Average annual number of patients receiving TB testing	892,000 (519,000–1,508,000)	829,000 (487,000–1,400,000)
Average annual number of true positive diagnoses	151,000 (100,000–215,000)	175,000 (120,000–245,000)
Average diagnosis cost per patient with suspected TB	$31 (25–38)	$45 (40–50)
Average diagnosis cost per true positive diagnosis	$181 (117–287)	$211 (136–334)
**Programmatic measures for DOTS treatment**		
Average annual DOTS treatment costs	$57 million (30–102 million)	$81 million (42–137 million)
Average treatment volume	57,000 (38,000–85,000)	69,000 (48,000–100,000)
Average annual number of true positive treatment initiations	122,000 (81,000–175,000)	147,000 (103,000–206,000)
Average number of annual cures	100,000 (66,000–146,000)	121,000 (84,000–172,000)
Average treatment cost per month	$84 (59–135)	$98 (67–147)
Average treatment cost per TB case initiated	$469 (321–761)	$556 (371–861)
Average treatment cost per TB case cured	$575 (396–914)	$675 (461–1,008)

All costs are given in 2011 US dollars. Results are based on US$30 Xpert per-test cost. Range in parentheses represents the 95% posterior interval for each estimate.

### Population Health Impact of Introducing Xpert

**Figure 3:**
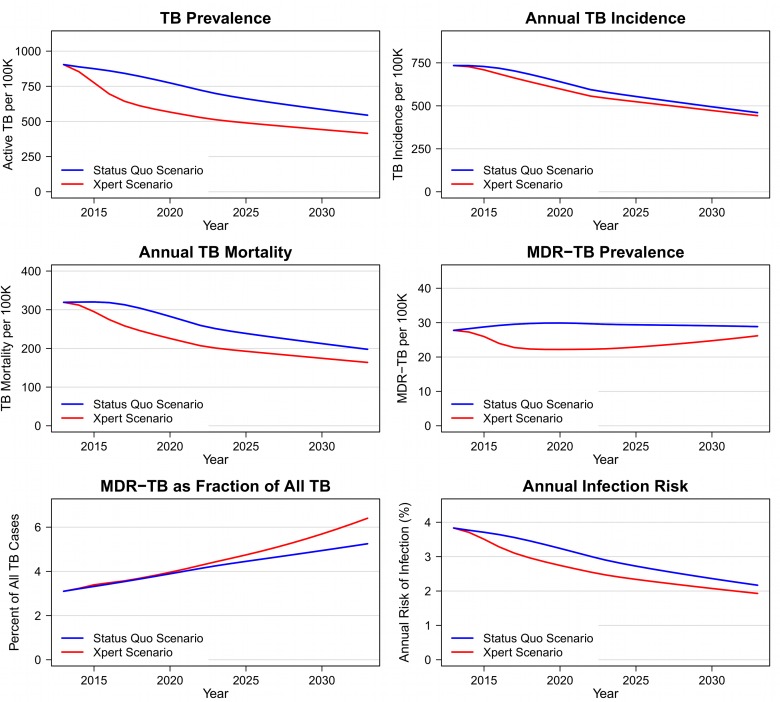
Epidemiologic outcomes in Xpert and status quo scenarios, 2012–2032.

Introduction of Xpert is projected to produce immediate and sustained changes in TB epidemiology (Figure 3). Within 10 y after the introduction of Xpert, prevalence would be lower by 186 (95% CI: 86–350) per 100,000 (28% [95% CI: 14–40]), incidence by 35 (13–79) per 100,000 (6% [2]–[13]), and annual TB mortality by 50 (23–89) per 100,000 (21% [10]–[32]), compared to status quo projections. The absolute number of MDR-TB cases after 10 y would be lower by 25% (6–44) in the Xpert scenario compared to the status quo scenario. The decline in MDR-TB cases parallels the overall decline in TB prevalence in these projections. There is no significant change expected in MDR-TB as a percentage of all TB under the Xpert scenario (4.3% [−17.5 to 34.6] greater after 10 y). Figure S2 shows the incremental differences between Xpert and the status quo for these health outcomes, including uncertainty intervals around these differences.

Summing the health effects of Xpert introduction over the first 10 y of implementation, this strategy is estimated to prevent 132,000 (95% CI: 55,000–284,000) of the estimated 2.6 million (1.7–4.3 million) new TB cases and 182,000 (97,000–302,000) of the estimated 1.2 million (0.6–2.0 million) TB deaths projected for southern Africa under the status quo.

### Health System Costs of Introducing Xpert

**Figure 4:**
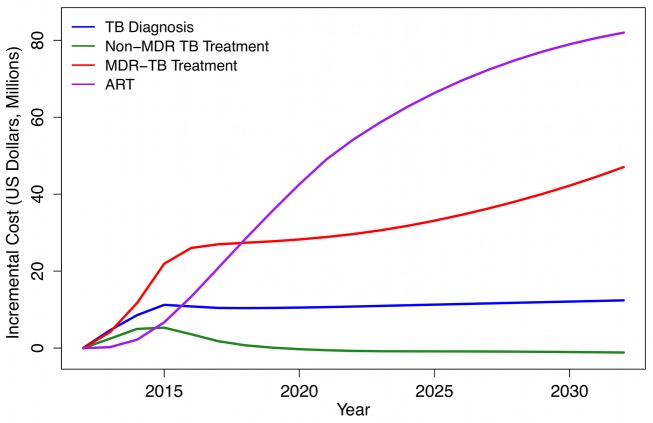
Incremental costs of Xpert strategy (based on US$30 Xpert per-test cost) compared to status quo strategy, by cost category, 2012–2032 (2011 US dollars).

Figure 4 shows the additional annual costs associated with the Xpert scenario compared to the status quo, subdivided by type of cost. TB program costs rise rapidly as Xpert scales up to full coverage over 2012–2015. While implementation of Xpert requires increased spending on TB diagnosis and treatment, the major financial impact of Xpert introduction in this region is on HIV treatment programs. This is because prompt TB treatment extends survival among TB/HIV-coinfected individuals, leading to increases in HIV treatment demand. The model predicts that at 10 y after Xpert introduction, HIV treatment costs will comprise 58% (95% CI: 40–72) of the total incremental costs associated with the Xpert strategy (assuming an Xpert per-test cost of US$30). Considering only the additional costs incurred by national DOTS programs, almost three-quarters (71% [47]–[87]) of these will be due to growth in TB treatment costs, with almost all of this increase coming from a higher volume of MDR-TB treatment.

### Cost-Effectiveness of Xpert Strategy versus the Status Quo

Table 3 shows ICERs for the Xpert strategy versus the status quo strategy under 10-y and 20-y analytic horizons and a range of Xpert costs. Assuming an Xpert cost of US$30 per test, the Xpert scenario is expected to avert approximately half a million DALYs during the first 10 y following introduction, at a cost of US$959 (95% CI: 633–1,485) per DALY averted.

**Table 3:** Cost-effectiveness results for Xpert algorithm compared to status quo algorithm in southern Africa.

Outcome	Xpert Cost		
	US$20	US$30	US$40
**10-y analytic horizon (costs and benefits summed over 2012–2022)**			
Incremental costs, health system	$401 million (248–623 million)	$460 million (294–699 million)	$520 million (333–772 million)
Incremental costs, DOTS program only	$225 million (119–378 million)	$284 million (166–448 million)	$344 million (209–522 million)
Incremental life-years saved	421,000 (234,000–679,000)	421,000 (234,000–679,000)	421,000 (234,000–679,000)
Incremental DALYs averted	480,000 (261,000–809,000)	480,000 (261,000–809,000)	480,000 (261,000–809,000)
Incremental cost per life-year saved^a^	$952 (606–1,326)	$1,093 (746–1,592)	$1,234 (836–1,872)
Incremental cost per DALY averted^a^	$836 (531–1,223)	$959 (633–1,485)	$1,083 (716–1,760)
**20-y analytic horizon (costs and benefits summed over 2012–2032)**			
Incremental costs, health system	$1,103 million (594–1,979 million)	$1,217 million (691–2,093 million)	1,330 (784–2,205)
Incremental costs, DOTS program only	$481 million (205–993 million)	$594 million (295–1,125 million)	707 (379–1,262)
Incremental life-years saved	1,500,000 (800,000–2,570,000)	1,500,000 (800,000–2,570,000)	1,500,000 (800,000–2,570,000)
Incremental DALYs averted	1,550,000 (800,000–2,770,000)	1,550,000 (800,000–2,770,000)	1,550,000 (800,000–2,770,000)
Incremental cost per life-year saved^a^	$734 (459–1,173)	$810 (504–1,311)	$885 (557–1,467)
Incremental cost per DALY averted^a^	$711 (422–1,187)	$784 (476–1,345)	$857 (523–1,534)

All costs are given in 2011 US dollars.

a ICERs calculated using health system costs (including DOTS costs). Both costs and health outcomes discounted at 3%. Range in parentheses represents the 95% posterior interval for each estimate.

**Figure 5:**
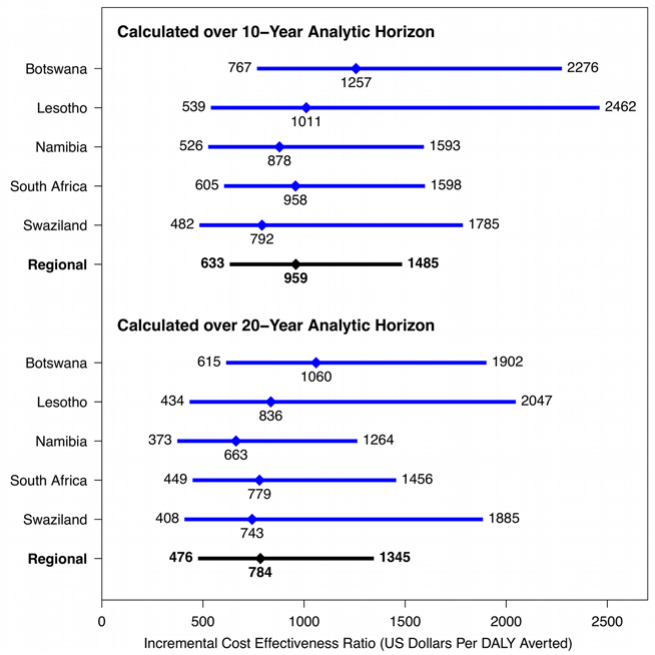
Cost-effectiveness of Xpert strategy compared to status quo strategy in five southern African countries (2011 US dollars). For each ratio, the diamond indicates the point estimate (mean incremental costs divided by mean incremental DALYs averted), and the bar indicates the width of the 95% posterior interval. Results based on US$30 Xpert per-test cost.

Figure 5 presents the costs per DALY averted through implementation of Xpert in each of the five southern African countries. In almost all cases, the cost-effectiveness ratios fall below the standard benchmarks for cost-effectiveness suggested by WHO, whereby interventions with cost-effectiveness ratios less than three-times annual per-capita GDP are regarded as potentially cost-effective, and interventions with cost-effectiveness ratios less than annual per-capita GDP are deemed very cost-effective. Among these five countries, per-capita GDP in 2010 ranged from above US$7,000 in South Africa and Botswana down to US$982 in Lesotho [73].

### Sensitivity Analyses

**Figure 6:**
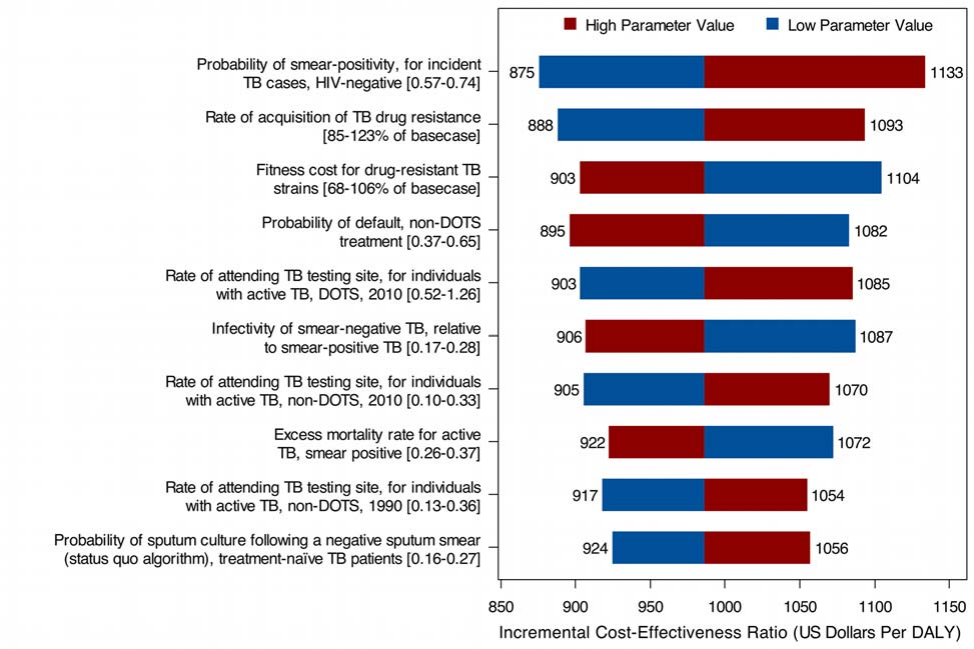
Results from univariate sensitivity analyses, showing the ten parameters with the greatest influence on the cost-effectiveness of Xpert compared to status quo, South Africa. Sensitivity analyses on the incremental cost per DALY averted (2011 US dollars) over a 10-y analytic horizon, assuming a US$30 Xpert per-test cost. In each one-way analysis, one parameter was varied ±1 standard deviation from its posterior mean, with all other variables fixed at their posterior means.

We conducted one-way sensitivity analyses for all model inputs. Figure 6 shows the results for South Africa for the ten parameters producing the greatest variation in the cost-effectiveness ratio when varied by ±1 standard deviation from their posterior means. While the overall uncertainty in model results—as expressed in the posterior intervals and in the cost-effectiveness acceptability curves described below—is not small, the uncertainty generated by any individual parameter is relatively small, and does not change the general conclusions of the study. Complete results, by country, for the one-way sensitivity analyses on all parameters are reported in Text S1. Partial rank correlation coefficients, which reflect a probabilistic approach to identifying influential parameters, were calculated for all model inputs based on the simulation results, and yielded conclusions that were largely consistent with those based on the one-way sensitivity analyses (results for South Africa presented in Figure S3).

The cost-effectiveness ratios presented in Table 3 and Figure 5 attempt to capture the major changes in health system resource use and health outcomes resulting from the adoption of the Xpert algorithm, including increases in TB treatment and HIV treatment volume. The increase in TB treatment volume is a direct consequence of better case-finding under the Xpert algorithm. The increase in ART volume is an indirect consequence of Xpert introduction, resulting from improved survival of TB/HIV-coinfected individuals who are currently receiving ART or who will go on to receive ART in the future. As shown in Figure 4, the increase in health system costs due to increased ART volume is substantial. In order to disentangle the direct effect of Xpert from this secondary effect through HIV survival, we constructed a scenario in which access to ART under a scaled-up Xpert approach was constrained to be the same as in the status quo scenario (as might be the case if the future HIV treatment budget were fixed and did not increase as a function of HIV treatment need). While artificial, this scenario allowed us to estimate the cost-effectiveness of Xpert adoption separate from the effects on HIV treatment. In this scenario, incremental costs and DALYs averted dropped by 35%–40% and 10%–15%, respectively, compared to the main analysis, and the cost per DALY averted (assuming a US$30 per-test cost for Xpert) dropped to US$656 (95% CI: 386–1,115) over a 10-y analytic horizon.

Further sensitivity analyses (described in Text S1) tested the robustness of the cost-effectiveness results to the use of clinical diagnosis as part of the status quo algorithm, to the removal of inpatient care from MDR-TB treatment, to the provision of empiric MDR-TB treatment while awaiting results from DST for all patients diagnosed with RIF resistance by Xpert, and to a revised assumption about ART cost trends, in which ART prices drop 50% over 10 y. Each of these changes produced a change in the 10-y ICER of <20% and did not change the qualitative conclusions about Xpert cost-effectiveness. Detailed three-way sensitivity analyses were conducted to understand how current coverage of culture (among treatment-naïve and treatment-experienced patients) and DST affected the incremental costs, health benefits, and cost-effectiveness of Xpert in each country. These analyses (Figure S4) show that if use of culture under the status quo algorithm is higher than the value used in the main analysis, this reduces the incremental costs and health benefits produced by adopting Xpert and results in a less favorable cost-effectiveness ratio. In some countries, very high values of culture use would result in the status quo strategy dominating the Xpert strategy, i.e., having lower costs and greater health benefits. The coverage levels that produce such a result (80% of all treatment- naïve and treatment-experienced TB patients diagnosed via culture), however, are unlikely to be in place at present, given current infrastructure and program constraints. Higher than expected DST access under the status quo would produce modest reductions in incremental costs and minimal changes in cost-effectiveness ratios.

We also considered an alternative Xpert algorithm that requires more aggressive investigation (via culture, chest X-ray, and antibiotic trial) of Xpert-determined TB-negative individuals with HIV-positive or unknown status, as described in recent South African Xpert guidelines [74]. The ICER for this aggressive Xpert algorithm, compared to the base-case Xpert algorithm evaluated in the main analysis, was US$2,128 (95% CI: 1,215–3,954) per DALY averted, suggesting that while this more aggressive algorithm may be cost-effective in some settings, limited programmatic resources might yield higher benefits by expanding access to a simplified Xpert algorithm.

Finally, we constructed cost-effectiveness acceptability curves to consider the likelihood that Xpert would be cost-effective under different thresholds for societal willingness to pay for an additional year of healthy life (Figure S5). If society were willing to pay up to the average per-capita GDP (US$6,850 for the region) for each averted DALY, our results suggest essentially no uncertainty in the conclusion that Xpert would be cost-effective. At a threshold of only US$1,000 (representing <15% of per-capita GDP in the region), the probability that Xpert would be regarded as cost-effective was 85%, when we considered the benefits that would accumulate over 20 y, or 55%, over a 10-y horizon.

## Discussion

In this study, we used a dynamic, calibrated mathematical model of TB to evaluate the potential health and economic consequences associated with scaling up the new Xpert MTB/RIF test in settings with high TB burden, prevalent MDR-TB, and high concurrent prevalence of HIV. Our modeling approach enables quantification of the population-level health effects of alternative diagnostic strategies, projections of impact over the short term and longer time horizons, and assessment of the economic impact and cost-effectiveness of scaling up Xpert compared to continuation of the status quo diagnostic approach.

Our results indicate that the introduction of the Xpert MTB/RIF diagnostic has the potential to produce a substantial reduction in TB morbidity and mortality in southern Africa. For individuals with smear-negative TB, the benefits of Xpert implementation would be immediate, leading to the diagnosis and early treatment of many individuals who would be missed by the conventional diagnostic algorithm. Over a longer time frame, the introduction of Xpert would reduce transmission and reduce the reservoir of latent TB infection in the population, but these secondary effects are smaller than might have been anticipated. Even accounting for indirect transmission benefits, we project that TB incidence will remain substantial after three decades of Xpert use, in the absence of other modifications to the status quo TB control strategy. This is due to the large existing pool of latently infected individuals whose progression to active disease would be unmitigated by improved diagnostics, and to the fact that a substantial fraction of the additional cases diagnosed using Xpert will be smear-negative cases, who are less likely to transmit infection than smear-positive cases.

Along with the projected health benefits of scaling up Xpert will come significantly increased demands on healthcare resources. The large increase in funding required under the Xpert scenario raises the question of affordability. Although our cost-effectiveness results suggest that the introduction of Xpert represents good value for money according to typical international benchmarks, it does not automatically follow that TB program budgets will be able to absorb these changes. Whereas current debate about the costs of Xpert roll-out focuses largely on equipment and consumables connected directly to the assay, our results show that the indirect cost consequences associated with improved case-finding overshadow the direct costs of diagnosis. If current guidelines are followed, the adoption of Xpert places three key demands on a health system that are additional to the direct costs of diagnosis: providing first-line TB treatment to the large number of additional pan-sensitive TB cases that will be identified, providing additional HIV treatment to coinfected individuals who will live longer as a result of better TB care, and providing second-line TB treatment to the limited number of individuals diagnosed with drug-resistant TB. While our analysis accounts for all three demands, we recognize that response to each of these demands could be evaluated as a separate policy question. Such analyses are beyond the scope of our present study, but it is nevertheless important to note how the economics of Xpert are dependent on the additional interventions triggered by Xpert introduction—which are sensitive to both epidemiologic context and policy decisions. It is likely that existing resources and infrastructure will be called upon to support the introduction of Xpert and the cascade of complementary services this will trigger, and our findings underscore the concern raised by other commentators regarding the possible pitfalls of introducing Xpert into health systems that are already facing capacity constraints [26],[29].

An important observation in this study is that substantial increases in HIV treatment costs are expected following introduction of Xpert. This critical insight has a large influence on the cost-effectiveness of Xpert that would be missed in simpler models that do not capture the concurrent dynamics of TB and HIV, and is consistent with other analyses pointing to the importance of HIV and ART access for TB outcomes in this setting [27],[75]. Sensitivity analyses show that if future HIV treatment access were limited by a hard budget constraint, this would actually result in a more attractive cost-effectiveness ratio for Xpert adoption (reducing the ICER to less than US$700 per DALY over a 10-y analytic horizon), with the subtraction of ART costs from the numerator of the ICER outweighing the reduction in health benefits in the denominator. Note that this finding provides no evidence about the appropriate level of ART access in the future, but does provide a clear illustration of the interlinked nature of TB and HIV policy in settings with dual epidemics. Although the absolute increase in HIV treatment spending would eventually be larger than the increase in TB program costs, the relative effects on total budgets for HIV and TB control are reversed; we estimate that introduction of Xpert would result in a 2% increase in HIV treatment costs after 10 y, but a 40% increase in the costs of TB control.

Providing treatment to additional cases diagnosed with MDR-TB represents another major component of the incremental costs of Xpert adoption. In our base-case analysis, we assumed that second-line TB treatment would be available for diagnosed MDR-TB cases, which resulted in an estimated 2- to 3-fold increase in the volume of MDR-TB treatment under an Xpert scale-up scenario. If second-line therapy were less available than we assumed, the cost-effectiveness of Xpert would actually improve in the short term (at the cost of faster growth in drug resistance), as the reduction in treatment costs would outweigh the reduction in survival among MDR-TB patients receiving ineffective first-line regimens. Recent empirical cost analyses suggest that MDR-TB care costs may be even higher than estimated in our analysis, with a South African study estimating per-patient costs of over US$17,000 during the inpatient phase of therapy alone, more than 40 times the cost of treating drug-sensitive TB [76]. While this might motivate the development of more efficient approaches to MDR-TB treatment, it also highlights the trade-offs involved in Xpert introduction.

Although the scenarios considered in this analysis assumed that DST would be used prior to the initiation of patients on second-line regimens, the availability of DST remains limited in some settings. Of note, the 67% positive predictive value of the Xpert test for RIF resistance in this setting suggests that a positive result on the Xpert RIF test would be insufficient evidence to initiate individuals on second-line regimens, and further screening would be necessary. Further, the benefits achieved through better detection and treatment of drug-resistant TB would be offset by increases in the number of cases developing resistance, resulting from Xpert's better case detection and the resulting increase in treatment volume. Consequently, the percentage of all TB cases with MDR-TB after 10 and 20 y is projected to be higher under the Xpert scenario, although this result is not statistically significant, and—given the overall reduction in TB prevalence produced by Xpert—the absolute number of MDR-TB cases would be lower than under the status quo.

A recent modeling study on Xpert introduction in three countries [77] reported an ICER of US$138 per DALY in South Africa for Xpert versus the status quo, which is around 5–8 times lower than the estimated ratios in our study. Because the prior study used a cohort model of patients with suspected TB, its results pertained only to the direct effects of diagnosis and treatment in a defined cohort, rather than reflecting the population-level health and economic consequences. The higher ratios in our study relate in part to our inclusion of HIV treatment costs, which are relevant to a health system or societal perspective. Exclusion of these costs from the prior analysis resulted in a more favorable assessment of Xpert, since the survival benefits of antiretroviral treatment were credited to Xpert when estimating DALYs averted, but at an implicit zero cost. An additional point of difference is that this prior study assumed no access to culture as part of the status quo algorithm, which also contributed to a lower cost-effectiveness ratio for Xpert when compared to the base-case assumptions about culture access used in our analysis. Another recent analysis looked at the use of Xpert for TB screening prior to ART initiation in South Africa. This analysis included ART costs in the cost-effectiveness ratio, and reported a cost-effectiveness ratio of US$5,100 per life-year saved for the Xpert algorithm compared to current diagnostics [78]. This analysis considered only the health benefits for the individual being screened, rather than counting the cases averted by reducing transmission, and focused on a population in which ART costs would dominate the cost-effectiveness ratio, and so it is understandable that the cost-effectiveness ratio was considerably higher than the cost per life-year saved estimated in our study.

Our analysis has several limitations. The application of any mathematical model of TB is inevitably limited by uncertainty regarding the true values of epidemiologic and programmatic parameters. Our approach aims to reduce this parameter uncertainty through calibration, and to provide a valid quantitative expression of what parameter uncertainty remains based on Bayesian statistical inference; however, the uncertainty associated with model structure is impossible to quantify without building and assessing the whole range of possible model structures that might be adopted. For example, the results of this analysis would be different if the interdependency of TB and HIV epidemics were not considered, or if the indirect effect of Xpert on TB transmission were not captured. It will therefore be important to undertake continued empirical research evaluating the impact of Xpert as it is rolled out in practice, with the information generated by these evaluation efforts used to progressively refine the mathematical models used to estimate long-term intervention effects.

In the results reported here, we constrained estimates on costs and health outcomes to account only for those that would accrue during either the first 10 y or the first 20 y following introduction of Xpert. While the choice of a limited time horizon acknowledges our increasing uncertainty about the distant future and reflects the immediacy of policy decisions, it also makes our results somewhat conservative. This is particularly true for the 10-y results, which truncate the full streams of future benefits that will be enjoyed by those patients who avert TB mortality or infection during the 10-y analysis period. Likewise, we observe that cost-effectiveness ratios are more attractive over the 20-y horizon than the 10-y horizon, reflecting the compounding benefits of interrupting transmission dynamics through better diagnosis and treatment. Moreover, the restriction of our study to adult populations will underestimate the total burden of disease that might be averted, with Xpert adoption likely to reduce pediatric TB through reduced exposure to actively infected adults as well as the direct application of the test for pediatric diagnosis [79],[80].

Finally, we note that the results of the present analysis emphasize the importance of interactions between TB and HIV epidemiology in settings where both are highly prevalent, but we caution against generalizing these results to regions where HIV rates are meaningfully different from those in southern Africa. Additional analyses are urgently needed to assess the consequences of introducing Xpert elsewhere, particularly regions of low HIV prevalence or with different TB drug resistance patterns. Similarly, this study focused on the relative benefits of the status quo algorithm and the Xpert algorithm suggested by WHO for diagnosis of patients with suspected TB in settings with high HIV burden. While this is an important comparison to make, there is abundant scope for considering a wide array of alternatives, for example, considering different potential roles for sputum smear microscopy or chest X-ray within diagnostic algorithms designed around Xpert, or use of Xpert for different purposes, such as prior to provision of INH preventive therapy for individuals with HIV, or as part of active case-finding efforts [81]. Because the model developed for this analysis reflects detailed structure relating both to HIV and to patterns of resistance to major anti-TB drugs, it offers substantial flexibility to accommodate adaptation to other settings. In view of these features, and our statistical approach to calibrate this model to available epidemiologic data, we envision that the model can provide a durable platform for evaluating an array of different diagnostic strategies in diverse settings in the future.

## Supporting Information

Figure S1 **Status quo and Xpert diagnostic algorithms.**
(PDF)

Figure S2 **Incremental difference in epidemiologic outcomes between Xpert and status quo scenarios, 2012–2032.**
(PDF)

Figure S3 **Partial rank correlation coefficients for ten parameters with greatest influence on the cost-effectiveness of Xpert compared to status quo, South Africa, 10-y time horizon.**
(PDF)

Figure S4 **Three-way sensitivity analyses showing effects of changes in culture and DST coverage on major study outcomes, by country.** (A) Botswana; (B) Lesotho; (C) Namibia; (D) South Africa; (E) Swaziland. Costs, DALYs, and ICERs assessed over a 10-y analytic horizon with a US$30 Xpert unit cost. All other parameters held at their mean posterior values.
(PDF)

Figure S5 **Cost-effectiveness acceptability curves showing probability that Xpert strategy is cost-effective as a function of willingness to pay for health benefits.**
(PDF)

Text S1 **Technical appendix.** Figures S1, S2, S3, S4, S5 are available as individual files, but are also included here for ease of access.
(PDF)

## Abbreviations

ART: antiretroviral therapy
DALY: disability-adjusted life-year
DST: drug sensitivity testing
GDP: gross domestic product
ICER: incremental cost-effectiveness ratio
INH: isoniazid
MDR-TB: multidrug-resistant tuberculosis
MDR+/XDR-TB: tuberculosis resistant to isoniazid and rifampicin plus one or more second-line drugs
RIF: rifampicin
TB: tuberculosis
WHO: World Health Organization
