# Supplementary material for: Population Health Impact and Cost-Effectiveness of Tuberculosis Diagnosis with Xpert MTB/RIF: A Dynamic Simulation and Economic Evaluation
Source: PLoS Med. 2012 Nov 20;9(11):e1001347. doi: 10.1371/journal.pmed.1001347 (PMC3502465; doi:10.1371/journal.pmed.1001347)
Supplement: Figure S1 — Status quo and Xpert diagnostic algorithms. (PDF) [file pmed.1001347.s001.pdf]

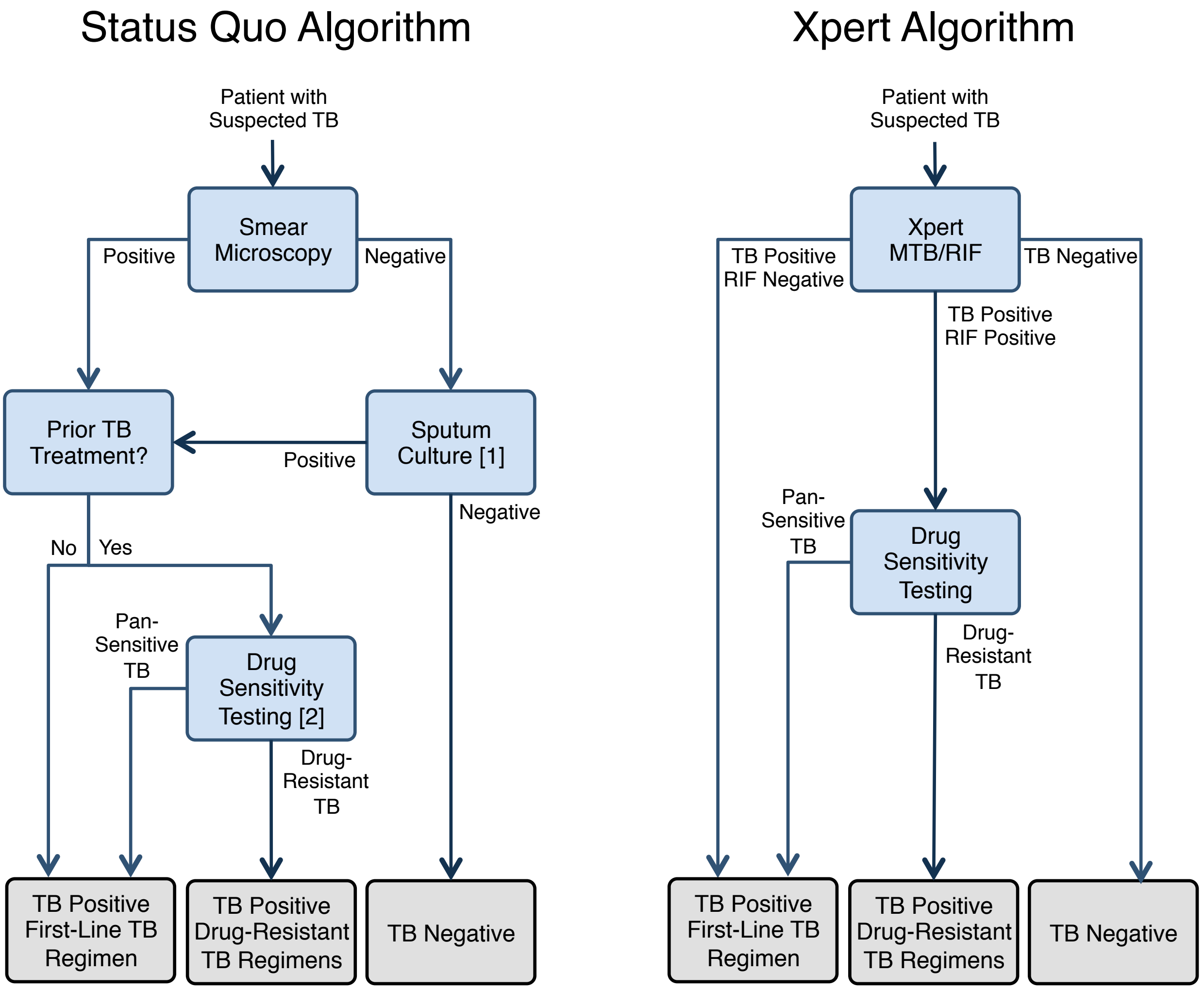

[1] Access to sputum culture assumed to be 80% for treatment-experienced, 20% for treatment-naïve patients.
[2] Access to DST assumed to be 80% for treatment-experienced patients.
