# Supplementary material for: Population Health Impact and Cost-Effectiveness of Tuberculosis Diagnosis with Xpert MTB/RIF: A Dynamic Simulation and Economic Evaluation
Source: PLoS Med. 2012 Nov 20;9(11):e1001347. doi: 10.1371/journal.pmed.1001347 (PMC3502465; doi:10.1371/journal.pmed.1001347)
Supplement: Figure S2 — Incremental difference in epidemiologic outcomes between Xpert and status quo scenarios, 2012–2032. (PDF) [file pmed.1001347.s002.pdf]

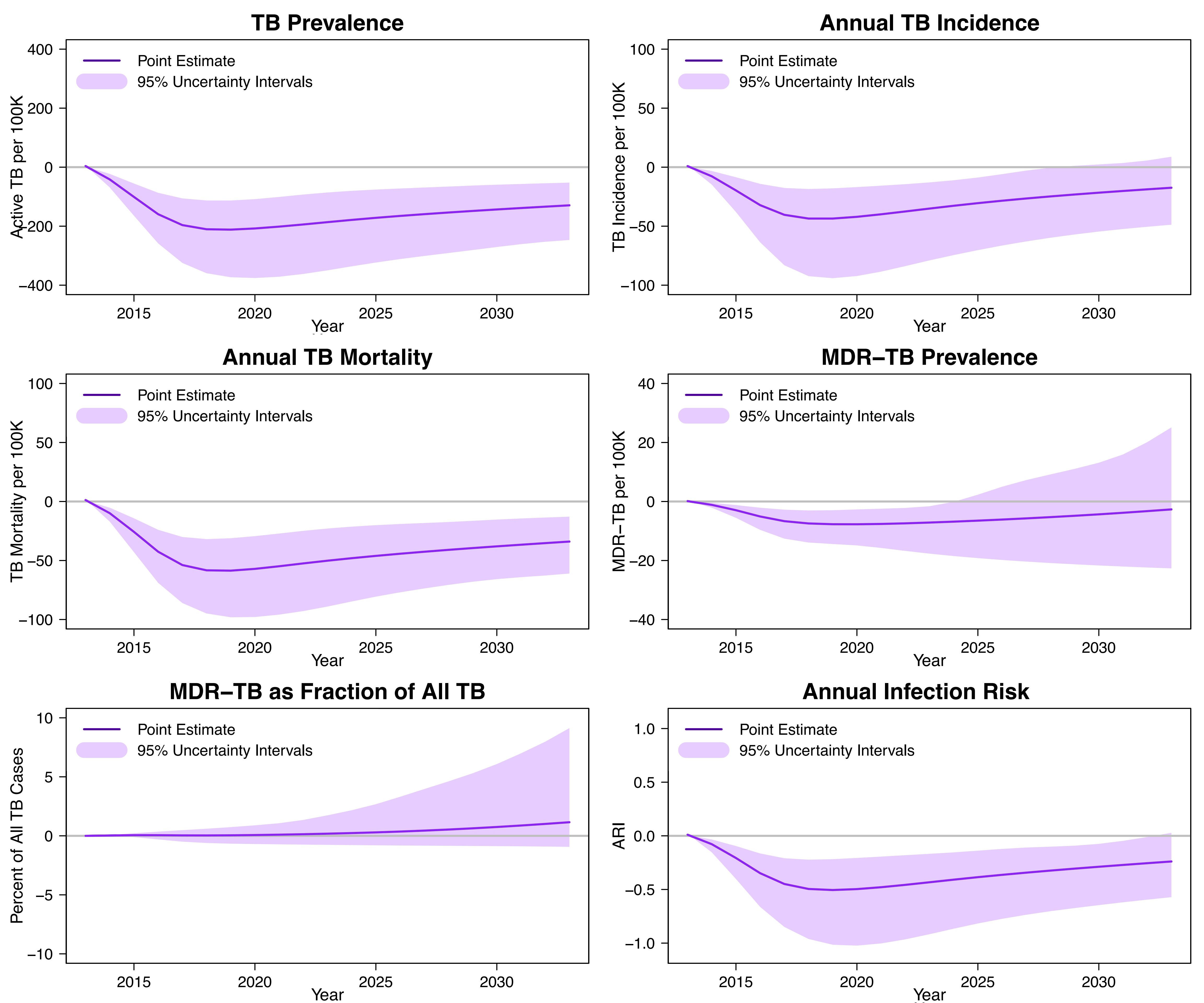

TB Prevalence
Point Estimate
95% Uncertainty Intervals
Active TB per 100K
400
200
0
−200
−400
2015
2020
2025
2030
Year
Annual TB Incidence
Point Estimate
95% Uncertainty Intervals
TB Incidence per 100K
100
50
0
−50
−100
2015
2020
2025
2030
Year
Annual TB Mortality
Point Estimate
95% Uncertainty Intervals
TB Mortality per 100K
100
50
0
−50
−100
2015
2020
2025
2030
Year
MDR−TB Prevalence
Point Estimate
95% Uncertainty Intervals
MDR−TB per 100K
40
20
0
−20
−40
2015
2020
2025
2030
Year
MDR−TB as Fraction of All TB
Point Estimate
95% Uncertainty Intervals
Percent of All TB Cases
10
5
0
−5
−10
2015
2020
2025
2030
Year
Annual Infection Risk
Point Estimate
95% Uncertainty Intervals
ARI
1.0
0.5
0.0
−0.5
−1.0
2015
2020
2025
2030
Year
