# Supplementary figures and images for: Population Health Impact and Cost-Effectiveness of Tuberculosis Diagnosis with Xpert MTB/RIF: A Dynamic Simulation and Economic Evaluation
Source: PLoS Med. 2012 Nov 20;9(11):e1001347. doi: 10.1371/journal.pmed.1001347 (PMC3502465; doi:10.1371/journal.pmed.1001347)

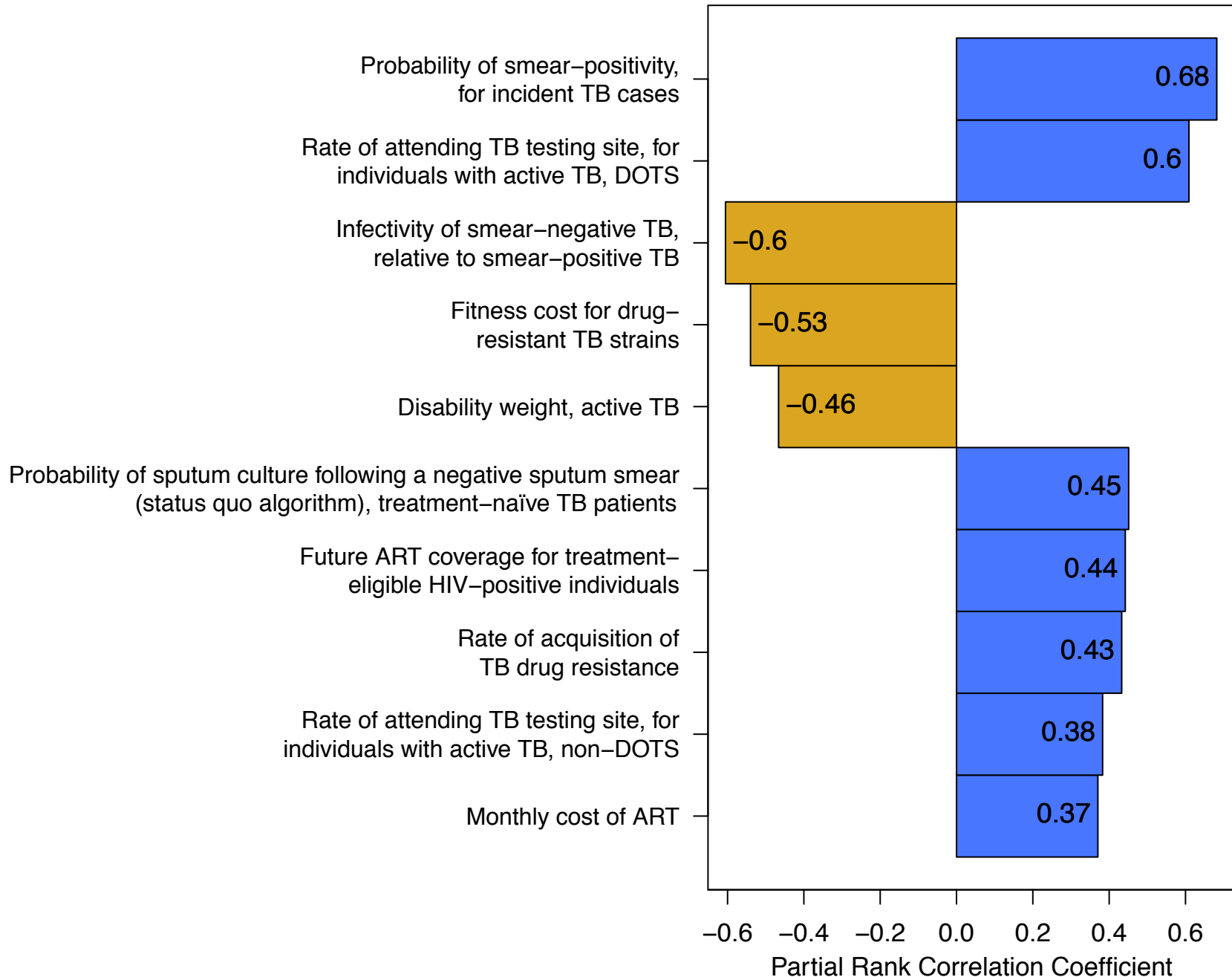

Supplement: Figure S3 — Partial rank correlation coefficients for ten parameters with greatest influence on the cost-effectiveness of Xpert compared to status quo, South Africa, 10-y time horizon. (PDF) [file pmed.1001347.s003.pdf]

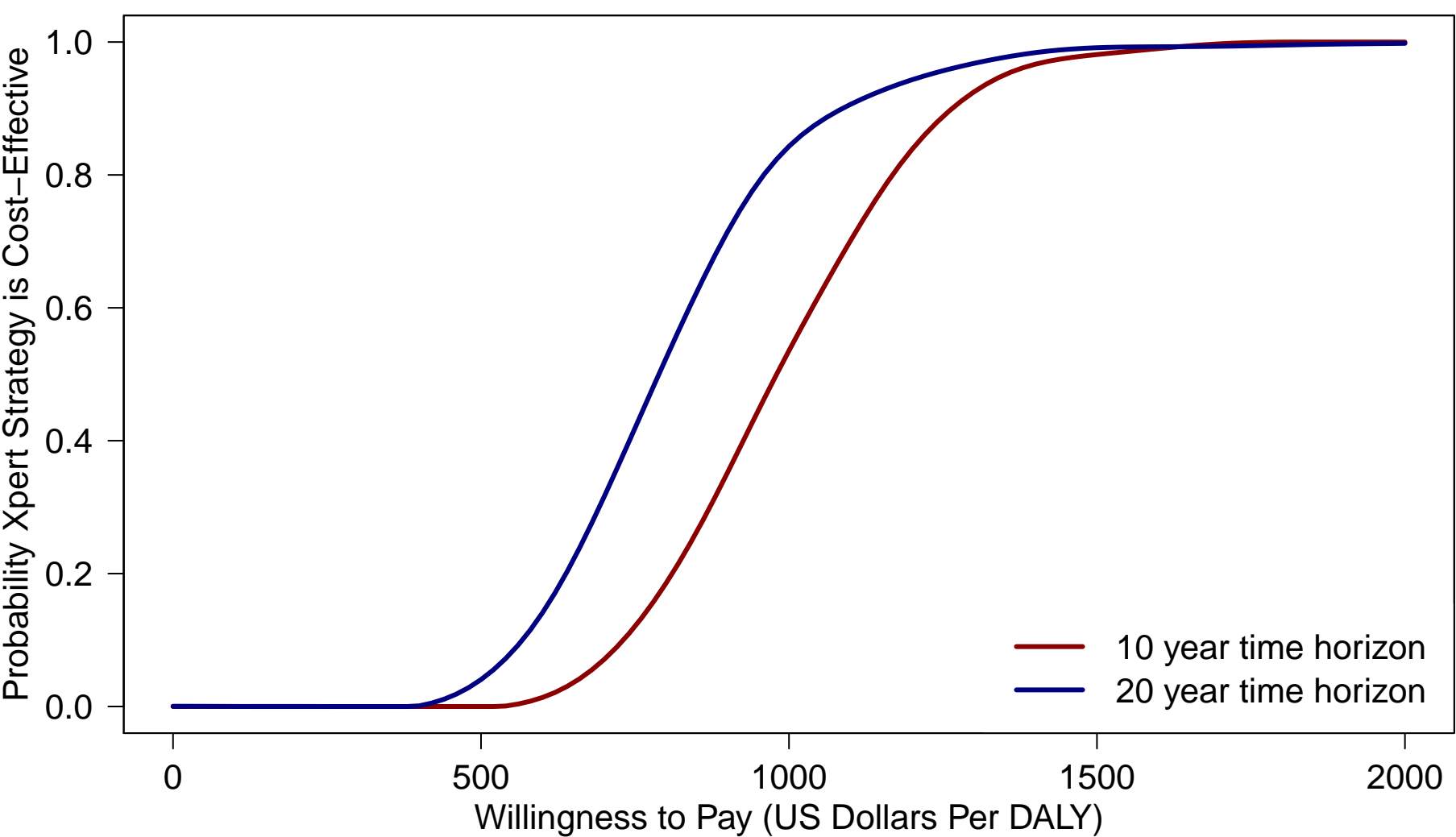

Supplement: Figure S5 — Cost-effectiveness acceptability curves showing probability that Xpert strategy is cost-effective as a function of willingness to pay for health benefits. (PDF) [file pmed.1001347.s005.pdf]
