# Supplementary material for: Population Health Impact and Cost-Effectiveness of Tuberculosis Diagnosis with Xpert MTB/RIF: A Dynamic Simulation and Economic Evaluation
Source: PLoS Med. 2012 Nov 20;9(11):e1001347. doi: 10.1371/journal.pmed.1001347 (PMC3502465; doi:10.1371/journal.pmed.1001347)
Supplement: Figure S4 — Three-way sensitivity analyses showing effects of changes in culture and DST coverage on major study outcomes, by country. (A) Botswana; (B) Lesotho; (C) Namibia; (D) South Africa; (E) Swaziland. Costs, DALYs, and ICERs assessed over a 10-y analytic horizon with a US$30 Xpert unit cost. All other parameters held at their mean posterior values. (PDF) [file pmed.1001347.s004.pdf]

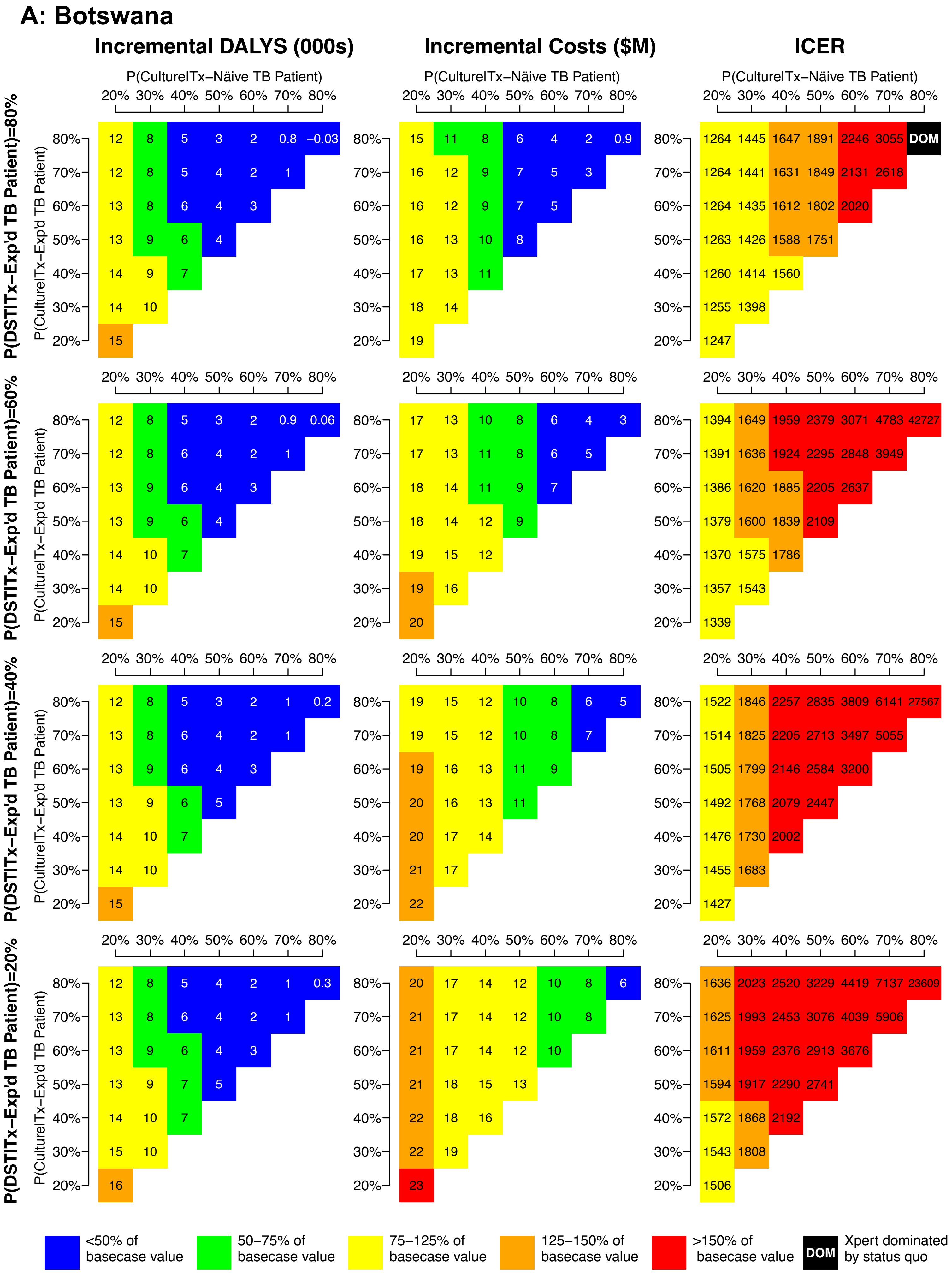
A: Botswana
Incremental DALYS (000s)
Incremental Costs ($M)
ICER
P(Culture|Tx−Näive TB Patient)
20% 30% 40% 50% 60% 70% 80%
P(Culture|Tx−Exp'd TB Patient)
80% 70% 60% 50% 40% 30% 20%
P(DST|Tx−Exp'd TB Patient)=80%
12 8 5 3 2 0.8 −0.03
12 8 5 4 2 1
13 8 6 4 3
13 9 6 4
14 9 7
14 10
15
15 11 8 6 4 2 0.9
16 12 9 7 5 3
16 12 9 7 5
16 13 10 8
17 13 11
18 14
19
1264 1445 1647 1891 2246 3055 DOM
1264 1441 1631 1849 2131 2618
1264 1435 1612 1802 2020
1263 1426 1588 1751
1260 1414 1560
1255 1398
1247
P(DST|Tx−Exp'd TB Patient)=60%
12 8 5 3 2 0.9 0.06
12 8 6 4 2 1
13 9 6 4 3
13 9 6 4
14 10 7
14 10
15
17 13 10 8 6 4 3
17 13 11 8 6 5
18 14 11 9 7
18 14 12 9
19 15 12
19 16
20
1394 1649 1959 2379 3071 4783 42727
1391 1636 1924 2295 2848 3949
1386 1620 1885 2205 2637
1379 1600 1839 2109
1370 1575 1786
1357 1543
1339
P(DST|Tx−Exp'd TB Patient)=40%
12 8 5 3 2 1 0.2
13 8 6 4 2 1
13 9 6 4 3
13 9 6 5
14 10 7
14 10
15
19 15 12 10 8 6 5
19 15 12 10 8 7
19 16 13 11 9
20 16 13 11
20 17 14
21 17
22
1522 1846 2257 2835 3809 6141 27567
1514 1825 2205 2713 3497 5055
1505 1799 2146 2584 3200
1492 1768 2079 2447
1476 1730 2002
1455 1683
1427
P(DST|Tx−Exp'd TB Patient)=20%
12 8 5 4 2 1 0.3
13 8 6 4 2 1
13 9 6 4 3
13 9 7 5
14 10 7
15 10
16
20 17 14 12 10 8 6
21 17 14 12 10 8
21 17 14 12 10
21 18 15 13
22 18 16
22 19
23
1636 2023 2520 3229 4419 7137 23609
1625 1993 2453 3076 4039 5906
1611 1959 2376 2913 3676
1594 1917 2290 2741
1572 1868 2192
1543 1808
1506
<50% of basecase value
50–75% of basecase value
75–125% of basecase value
125–150% of basecase value
>150% of basecase value
DOM
Xpert dominated by status quo

B: Lesotho

Columns: P(Culture|Tx−Näive TB Patient); rows: P(Culture|Tx−Exp'd TB Patient)

P(DST|Tx−Exp'd TB Patient)=80%

Incremental DALYS (000s)

	20%	30%	40%	50%	60%	70%	80%
80%	15	10	6	4	2	1	0.06
70%	15	10	7	4	3	1	
60%	15	10	7	5	3		
50%	16	11	7	5			
40%	16	11	8				
30%	17	12					
20%	18						

Incremental Costs ($M)

	20%	30%	40%	50%	60%	70%	80%
80%	15	13	11	9	8	7	6
70%	15	13	11	10	9	7	
60%	16	13	11	10	9		
50%	16	13	12	10			
40%	16	14	12				
30%	17	14					
20%	17						

ICER

	20%	30%	40%	50%	60%	70%	80%
80%	1038	1331	1740	2378	3579	6935	114751
70%	1031	1311	1692	2260	3250	5555	
60%	1021	1288	1638	2136	2938		
50%	1010	1261	1578	2007			
40%	997	1229	1512				
30%	980	1192					
20%	959						

P(DST|Tx−Exp'd TB Patient)=60%

Incremental DALYS (000s)

	20%	30%	40%	50%	60%	70%	80%
80%	15	10	6	4	2	1	0.1
70%	15	10	7	4	3	1	
60%	15	10	7	5	3		
50%	16	11	7	5			
40%	16	11	8				
30%	17	12					
20%	18						

Incremental Costs ($M)

	20%	30%	40%	50%	60%	70%	80%
80%	16	13	11	10	9	8	7
70%	16	13	12	10	9	8	
60%	16	14	12	10	9		
50%	16	14	12	11			
40%	17	14	12				
30%	17	15					
20%	17						

ICER

	20%	30%	40%	50%	60%	70%	80%
80%	1064	1371	1798	2462	3697	7028	62747
70%	1056	1349	1747	2338	3359	5675	
60%	1046	1324	1690	2209	3036		
50%	1034	1295	1626	2072			
40%	1019	1261	1555				
30%	1001	1221					
20%	978						

P(DST|Tx−Exp'd TB Patient)=40%

Incremental DALYS (000s)

	20%	30%	40%	50%	60%	70%	80%
80%	15	10	6	4	2	1	0.2
70%	15	10	7	4	3	1	
60%	15	10	7	5	3		
50%	16	11	7	5			
40%	16	11	8				
30%	17	12					
20%	18						

Incremental Costs ($M)

	20%	30%	40%	50%	60%	70%	80%
80%	16	14	12	10	9	8	7
70%	16	14	12	11	9	8	
60%	16	14	12	11	10		
50%	17	14	12	11			
40%	17	15	13				
30%	17	15					
20%	18						

ICER

	20%	30%	40%	50%	60%	70%	80%
80%	1090	1411	1856	2544	3809	7106	44612
70%	1081	1387	1801	2414	3462	5783	
60%	1070	1360	1741	2279	3131		
50%	1057	1329	1673	2136			
40%	1041	1293	1598				
30%	1021	1249					
20%	996						

P(DST|Tx−Exp'd TB Patient)=20%

Incremental DALYS (000s)

	20%	30%	40%	50%	60%	70%	80%
80%	15	10	6	4	2	1	0.2
70%	15	10	7	4	3	2	
60%	15	10	7	5	3		
50%	16	11	8	5			
40%	16	11	8				
30%	17	12					
20%	18						

Incremental Costs ($M)

	20%	30%	40%	50%	60%	70%	80%
80%	16	14	12	11	10	9	8
70%	17	14	12	11	10	9	
60%	17	14	13	11	10		
50%	17	15	13	12			
40%	17	15	13				
30%	18	15					
20%	18						

ICER

	20%	30%	40%	50%	60%	70%	80%
80%	1115	1447	1908	2619	3910	7176	36078
70%	1104	1422	1851	2484	3556	5878	
60%	1093	1393	1787	2343	3216		
50%	1078	1360	1717	2194			
40%	1061	1321	1637				
30%	1039	1275					
20%	1012						

Legend (cell colours):
- Blue: <50% of basecase value
- Green: 50−75% of basecase value
- Yellow: 75−125% of basecase value
- Orange: 125−150% of basecase value
- Red: >150% of basecase value

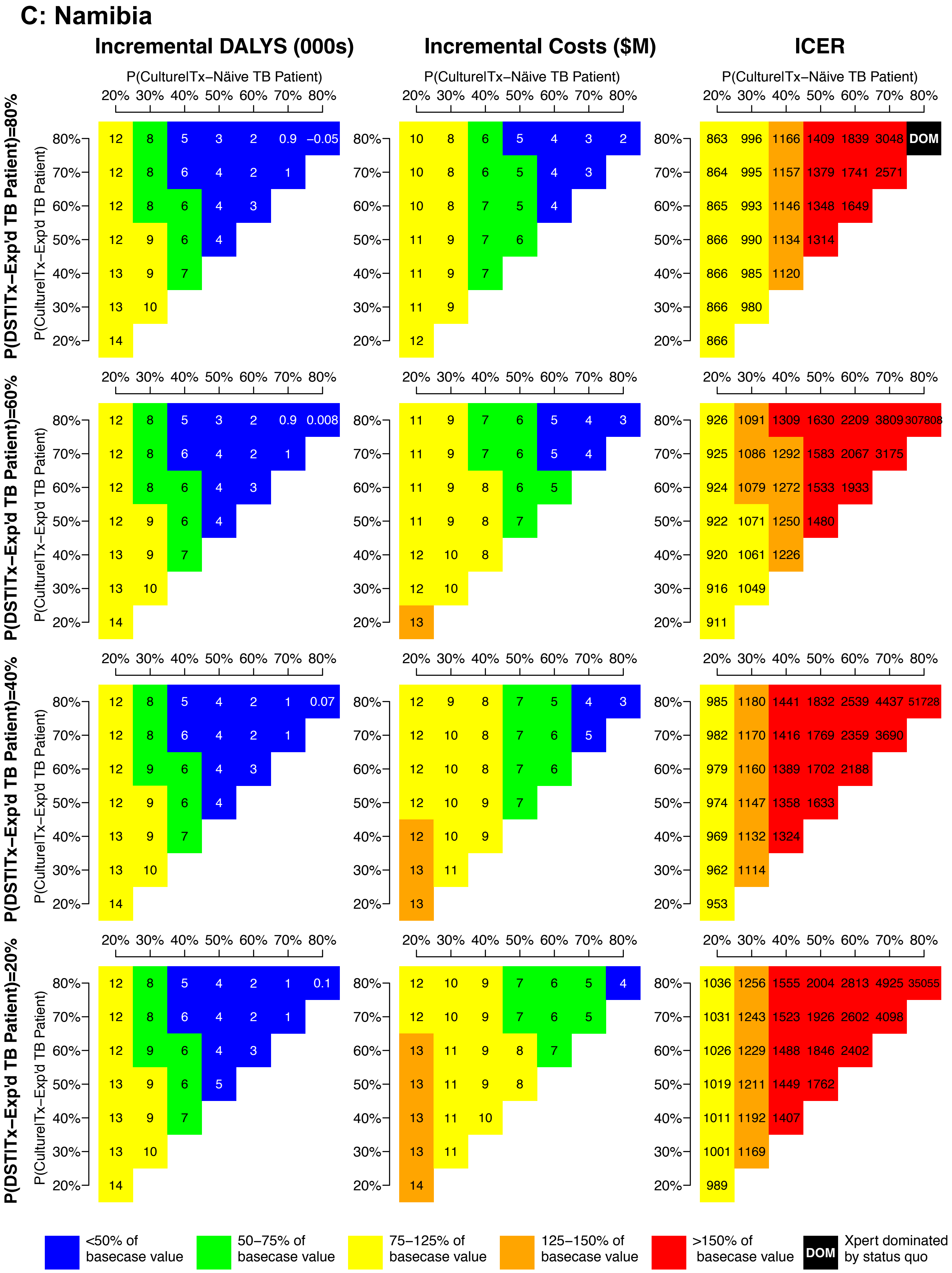
C: Namibia
Incremental DALYS (000s)
Incremental Costs ($M)
ICER
P(Culture|Tx–Näive TB Patient)
20% 30% 40% 50% 60% 70% 80%
P(DST|Tx–Exp'd TB Patient)=80%
P(Culture|Tx–Exp'd TB Patient)
80% 12 8 5 3 2 0.9 −0.05
70% 12 8 6 4 2 1
60% 12 8 6 4 3
50% 12 9 6 4
40% 13 9 7
30% 13 10
20% 14
80% 10 8 6 5 4 3 2
70% 10 8 6 5 4 3
60% 10 8 7 5 4
50% 11 9 7 6
40% 11 9 7
30% 11 9
20% 12
80% 863 996 1166 1409 1839 3048 DOM
70% 864 995 1157 1379 1741 2571
60% 865 993 1146 1348 1649
50% 866 990 1134 1314
40% 866 985 1120
30% 866 980
20% 866
P(DST|Tx–Exp'd TB Patient)=60%
80% 12 8 5 3 2 0.9 0.008
70% 12 8 6 4 2 1
60% 12 8 6 4 3
50% 12 9 6 4
40% 13 9 7
30% 13 10
20% 14
80% 11 9 7 6 5 4 3
70% 11 9 7 6 5 4
60% 11 9 8 6 5
50% 11 9 8 7
40% 12 10 8
30% 12 10
20% 13
80% 926 1091 1309 1630 2209 3809 307808
70% 925 1086 1292 1583 2067 3175
60% 924 1079 1272 1533 1933
50% 922 1071 1250 1480
40% 920 1061 1226
30% 916 1049
20% 911
P(DST|Tx–Exp'd TB Patient)=40%
80% 12 8 5 4 2 1 0.07
70% 12 8 6 4 2 1
60% 12 9 6 4 3
50% 12 9 6 4
40% 13 9 7
30% 13 10
20% 14
80% 12 9 8 7 5 4 3
70% 12 10 8 7 6 5
60% 12 10 8 7 6
50% 12 10 9 7
40% 12 10 9
30% 13 11
20% 13
80% 985 1180 1441 1832 2539 4437 51728
70% 982 1170 1416 1769 2359 3690
60% 979 1160 1389 1702 2188
50% 974 1147 1358 1633
40% 969 1132 1324
30% 962 1114
20% 953
P(DST|Tx–Exp'd TB Patient)=20%
80% 12 8 5 4 2 1 0.1
70% 12 8 6 4 2 1
60% 12 9 6 4 3
50% 13 9 6 5
40% 13 9 7
30% 13 10
20% 14
80% 12 10 9 7 6 5 4
70% 12 10 9 7 6 5
60% 13 11 9 8 7
50% 13 11 9 8
40% 13 11 10
30% 13 11
20% 14
80% 1036 1256 1555 2004 2813 4925 35055
70% 1031 1243 1523 1926 2602 4098
60% 1026 1229 1488 1846 2402
50% 1019 1211 1449 1762
40% 1011 1192 1407
30% 1001 1169
20% 989
<50% of basecase value
50–75% of basecase value
75–125% of basecase value
125–150% of basecase value
>150% of basecase value
DOM Xpert dominated by status quo

D: South Africa

Columns: P(Culture|Tx–Näive TB Patient). Rows: P(Culture|Tx–Exp'd TB Patient).

P(DST|Tx–Exp'd TB Patient)=80%

Incremental DALYS (000s)

	20%	30%	40%	50%	60%	70%	80%
80%	451	327	233	159	99	50	10
70%	457	334	241	167	108	59	
60%	465	343	249	176	117		
50%	474	352	260	187			
40%	484	363	271				
30%	496	377					
20%	510						

Incremental Costs ($M)

	20%	30%	40%	50%	60%	70%	80%
80%	440	362	300	248	203	165	131
70%	447	370	308	257	213	175	
60%	455	380	318	267	224		
50%	465	390	330	279			
40%	475	402	343				
30%	488	416					
20%	502						

ICER

	20%	30%	40%	50%	60%	70%	80%
80%	976	1107	1286	1559	2048	3267	13548
70%	978	1107	1282	1540	1980	2966	
60%	980	1107	1276	1519	1913		
50%	981	1107	1270	1496			
40%	983	1106	1262				
30%	984	1104					
20%	985						

P(DST|Tx–Exp'd TB Patient)=60%

Incremental DALYS (000s)

	20%	30%	40%	50%	60%	70%	80%
80%	456	333	238	164	105	56	15
70%	463	340	246	172	113	64	
60%	470	348	255	182	123		
50%	479	358	265	192			
40%	489	369	277				
30%	501	382					
20%	516						

Incremental Costs ($M)

	20%	30%	40%	50%	60%	70%	80%
80%	493	417	356	306	262	225	191
70%	500	425	365	314	271	234	
60%	508	434	374	324	282		
50%	516	443	384	335			
40%	526	454	396				
30%	538	467					
20%	551						

ICER

	20%	30%	40%	50%	60%	70%	80%
80%	1081	1255	1495	1860	2503	4015	12615
70%	1081	1251	1482	1823	2399	3628	
60%	1080	1245	1467	1784	2296		
50%	1078	1239	1450	1743			
40%	1076	1232	1431				
30%	1073	1223					
20%	1069						

P(DST|Tx–Exp'd TB Patient)=40%

Incremental DALYS (000s)

	20%	30%	40%	50%	60%	70%	80%
80%	462	338	244	170	111	62	21
70%	468	345	252	178	119	70	
60%	476	354	261	187	128		
50%	484	363	271	198			
40%	495	374	283				
30%	507	388					
20%	521						

Incremental Costs ($M)

	20%	30%	40%	50%	60%	70%	80%
80%	544	471	411	362	320	283	250
70%	551	478	419	370	328	291	
60%	558	486	427	379	337		
50%	566	495	437	389			
40%	575	505	448				
30%	586	517					
20%	598						

ICER

	20%	30%	40%	50%	60%	70%	80%
80%	1180	1392	1686	2129	2893	4585	11946
70%	1177	1383	1664	2077	2760	4149	
60%	1173	1373	1641	2023	2627		
50%	1168	1362	1614	1965			
40%	1163	1348	1585				
30%	1156	1333					
20%	1148						

P(DST|Tx–Exp'd TB Patient)=20%

Incremental DALYS (000s)

	20%	30%	40%	50%	60%	70%	80%
80%	467	344	250	176	116	67	27
70%	473	351	257	184	125	76	
60%	481	359	266	193	134		
50%	490	369	276	204			
40%	500	380	288				
30%	512	393					
20%	526						

Incremental Costs ($M)

	20%	30%	40%	50%	60%	70%	80%
80%	590	519	461	412	371	335	303
70%	596	525	468	420	379	343	
60%	602	532	475	428	387		
50%	610	540	484	437			
40%	618	549	494				
30%	628	560					
20%	639						

ICER

	20%	30%	40%	50%	60%	70%	80%
80%	1264	1509	1846	2349	3196	4977	11385
70%	1259	1496	1818	2285	3043	4518	
60%	1253	1482	1786	2218	2889		
50%	1245	1465	1752	2147			
40%	1237	1447	1715				
30%	1227	1425					
20%	1215						

Legend (cell colours): blue = <50% of basecase value; green = 50–75% of basecase value; yellow = 75–125% of basecase value; orange = 125–150% of basecase value; red = >150% of basecase value

E: Swaziland

Columns: P(Culture|Tx–Näive TB Patient); Rows: P(Culture|Tx–Exp'd TB Patient)

P(DST|Tx–Exp'd TB Patient)=80%

Incremental DALYS (000s)

	20%	30%	40%	50%	60%	70%	80%
80%	15	11	7	5	3	1	0.1
70%	16	11	7	5	3	2	
60%	16	11	8	5	3		
50%	16	12	8	6			
40%	17	12	9				
30%	18	13					
20%	18						

Incremental Costs ($M)

	20%	30%	40%	50%	60%	70%	80%
80%	12	9	7	6	5	4	3
70%	12	9	8	6	5	4	
60%	12	10	8	7	5		
50%	12	10	8	7			
40%	13	10	9				
30%	13	11					
20%	14						

ICER

	20%	30%	40%	50%	60%	70%	80%
80%	758	877	1041	1292	1758	3020	27809
70%	759	875	1031	1263	1665	2598	
60%	759	872	1021	1232	1575		
50%	759	869	1008	1200			
40%	758	864	994				
30%	758	858					
20%	756						

P(DST|Tx–Exp'd TB Patient)=60%

Incremental DALYS (000s)

	20%	30%	40%	50%	60%	70%	80%
80%	16	11	7	5	3	1	0.2
70%	16	11	7	5	3	2	
60%	16	11	8	5	4		
50%	17	12	8	6			
40%	17	12	9				
30%	18	13					
20%	18						

Incremental Costs ($M)

	20%	30%	40%	50%	60%	70%	80%
80%	13	10	8	7	6	5	4
70%	13	10	9	7	6	5	
60%	13	11	9	7	6		
50%	13	11	9	8			
40%	14	11	10				
30%	14	12					
20%	15						

ICER

	20%	30%	40%	50%	60%	70%	80%
80%	812	957	1158	1469	2039	3510	19205
70%	811	952	1143	1428	1918	3017	
60%	809	946	1126	1384	1801		
50%	807	938	1107	1338			
40%	805	929	1085				
30%	801	919					
20%	796						

P(DST|Tx–Exp'd TB Patient)=40%

Incremental DALYS (000s)

	20%	30%	40%	50%	60%	70%	80%
80%	16	11	7	5	3	1	0.3
70%	16	11	8	5	3	2	
60%	16	11	8	5	4		
50%	17	12	8	6			
40%	17	12	9				
30%	18	13					
20%	18						

Incremental Costs ($M)

	20%	30%	40%	50%	60%	70%	80%
80%	14	11	9	8	7	6	5
70%	14	11	10	8	7	6	
60%	14	12	10	8	7		
50%	14	12	10	9			
40%	15	12	10				
30%	15	13					
20%	15						

ICER

	20%	30%	40%	50%	60%	70%	80%
80%	864	1034	1272	1637	2296	3924	15962
70%	861	1026	1251	1584	2151	3381	
60%	858	1017	1227	1529	2010		
50%	854	1005	1201	1470			
40%	849	992	1172				
30%	843	977					
20%	835						

P(DST|Tx–Exp'd TB Patient)=20%

Incremental DALYS (000s)

	20%	30%	40%	50%	60%	70%	80%
80%	16	11	7	5	3	2	0.4
70%	16	11	8	5	3	2	
60%	16	11	8	6	4		
50%	17	12	8	6			
40%	17	12	9				
30%	18	13					
20%	19						

Incremental Costs ($M)

	20%	30%	40%	50%	60%	70%	80%
80%	14	12	10	9	8	7	6
70%	15	12	10	9	8	7	
60%	15	12	11	9	8		
50%	15	13	11	10			
40%	15	13	11				
30%	16	13					
20%	16						

ICER

	20%	30%	40%	50%	60%	70%	80%
80%	911	1103	1372	1784	2516	4249	14334
70%	907	1093	1346	1721	2350	3673	
60%	902	1080	1317	1655	2189		
50%	896	1065	1285	1585			
40%	889	1048	1249				
30%	880	1028					
20%	869						

Legend: <50% of basecase value (blue); 50–75% of basecase value (green); 75–125% of basecase value (yellow); 125–150% of basecase value (orange); >150% of basecase value (red)
