# Supplementary material for: Population Health Impact and Cost-Effectiveness of Tuberculosis Diagnosis with Xpert MTB/RIF: A Dynamic Simulation and Economic Evaluation
Source: PLoS Med. 2012 Nov 20;9(11):e1001347. doi: 10.1371/journal.pmed.1001347 (PMC3502465; doi:10.1371/journal.pmed.1001347)
Supplement: Text S1 — Technical appendix. Figures S1, S2, S3, S4, S5 are available as individual files, but are also included here for ease of access. (PDF) [file pmed.1001347.s006.pdf]

Text S1. TECHNICAL APPENDIX

Population health impact and cost-effectiveness of tuberculosis diagnosis with Xpert MTB/RIF: a dynamic simulation and economic evaluation

Nicolas A Menzies, Ted Cohen, Hsien-Ho Lin, Megan Murray, Joshua A Salomon

Contents

1 Model overview and structure ... 2
2 Transitions between model states and subdivisions ... 4
3 Model parameterization ... 8
4 Model calibration ... 17
5 Sensitivity and uncertainty analyses ... 20
6 References ... 39
7 Supplementary figures ... 45

1 Model overview and structure

Analyses were conducted using a dynamic compartmental model of tuberculosis (TB) in adult populations. The model simulates transitions between health states deterministically, recalculating the population distribution across states in discrete monthly time steps. The model was constructed and run using **R** statistical computing software.

The model follows the conventions of earlier TB models [1-7], with additional detail to accommodate evaluation of alternative diagnostic strategies. The model structure is defined by a set of core TB states, and these states are further subdivided to account for: (1) aspects of HIV infection, progression and treatment relevant to TB epidemiology; (2) multiple circulating TB strains, with different drug resistance profiles; and (3) tracking of TB treatment history.

1.1 Core TB states

The core TB states capture important features of TB transmission, natural history, and treatment. Eight states are included. Individuals who have never been infected reside in the susceptible state. Those who are infected but do not have active disease are in the latent infection/recovered state. Active disease is categorized as smear-negative or smear-positive. Smear-negative or smear-positive active cases may be treated either through the national TB control program (DOTS), or through providers outside of the national program (non-DOTS).

1.2 HIV subdivisions

HIV co-infection can alter the rate of progression of TB disease, with HIV-infected individuals having a higher probability of primary progressive TB upon initial infection [8,9], a higher rate of breakdown from latent infection to active TB [10], a lower probability of smear-positivity amongst those with active disease [11-13] and higher mortality rates [11,14,15]. The HIV sub-model draws on structure and assumptions from an array of published HIV models [16-19]. There are seven HIV subdivisions. Individuals may be HIV-negative, they may be in one of three categories reflecting untreated HIV infection with a specified CD4 cell count (>350 cells/μL, 200-350 cells/μL, and <200 cells/μL), or they may be receiving antiretroviral therapy (ART) in one of three categories distinguished by the CD4 count at treatment initiation.

1.3 Drug resistance subdivisions

Five subdivisions were created to account for differences in drug resistance among circulating TB strains, including: (1) pan-sensitive TB, (2) isoniazid (INH) mono-resistant TB, (3) rifampicin (RIF) mono-resistant TB, (4) resistance to both INH and RIF (MDR-TB), and (5) resistance to INH and RIF plus one or more second-line drugs (MDR+/XDR-TB).

1.4 Treatment history subdivisions

A final subdivision of model states distinguishes treatment-naïve from treatment-experienced individuals, as diagnostic algorithms may dictate different confirmatory tests depending on an individual's history of prior treatment.

1.5 Summary of model structure

At any point in time, all individuals in the model are categorized by the combination of their TB status and their status with respect to each of the three subdivisions. Thus, each of the 8 core states is 'exploded' into 70 unique sub-states (resulting from 7 HIV categories × 5 drug resistance categories × 2 treatment history categories), which yields a total of 8 × 70 = 560 unique compartments in the model. We note that some of these 560 compartments are null, in instances where the crossing of specific categories is meaningless; for example, susceptible individuals are defined by having never been infected, which means that they cannot be characterized in terms of a TB strain with a specific drug resistance profile.

2 Transitions between model states and subdivisions

The model transitions may be represented by a set of difference equations. Appendix Table 1 defines the general notation used in the formal description of the model that follows.

Appendix Table 1. Definition of core model states and transitions.

Symbol	Description
	Core model states
X_1	Number of individuals in the susceptible state at time t
X_2	Number of individuals in the latent/recovered state at time t
X_3	Number of individuals in the smear-negative active TB state at time t
X_4	Number of individuals in the smear-positive active TB state at time t
X_5	Number of individuals in smear-negative DOTS treatment state, at time t
X_6	Number of individuals in smear-negative non-DOTS treatment state, at time t
X_7	Number of individuals in smear-positive DOTS treatment state, at time t
X_8	Number of individuals in smear-positive non-DOTS treatment state, at time t
	Time-varying model transitions
η_t	New entrants at time t
λ_t	Force of infection at time t
γ_{Dt}, γ_{Nt}	Rate of attending TB testing site, in DOTS (D) or non-DOTS (N) program, for individuals with active TB, at time t
y_{Dit}, y_{Nit}	Probability of positive diagnosis for individuals attending testing site in DOTS or non-DOTS program, for state $i \in \{3,4\}$, at time t
h_{Dit}, h_{Nit}	Probability of loss to follow-up between initial presentation and treatment initiation, for individuals attending testing site in DOTS or non-DOTS program, for state $i \in \{3,4\}$, at time t
δ_{it}	Default rate for treatment state $i \in \{5,6,7,8\}$, at time t
g_{it}	Probability of treatment success, for individuals completing treatment in state $i \in \{5,6,7,8\}$, at time t
μ_{it}	All-cause mortality rate for model state *i* at time *t*, calculated as the sum of background mortality at time t (μ_{bt}), and disease-specific excess mortality (μ_{TB}, μ_{HIV}, μ_{TB-HIV})
	Time-invariant model transitions
m	Partial immunity afforded by prior infection
p	Probability of fast breakdown to active TB, for new infections
f	Probability of smear positivity, for incident TB cases
τ	Rate of breakdown from latent / recovered to active TB
α	Rate of conversion from smear-negative to smear-positive active TB
σ	Rate of self-cure for active TB
κ_i	Rate of treatment completion for treatment state i
v	Probability that failed treatment cases are correctly identified and returned to treatment

2.1 Transitions between core TB states

We begin with a set of model equations that describe changes in the population distribution across the eight core TB states between one time step and the next. In the following equations X_i indicates the number of residents in state i at time t, and $\dot{X}_i$ (with a dot above the X) indicates the number of residents in state i at time $t+1$.

$$\dot{X}_1 = X_1 + \eta_t - X_1\lambda_t - X_1\mu_{1t}$$

$$\dot{X}_2 = X_2 + X_1\lambda_t(1-p) - X_2(1-m)\lambda_t p + X_3\sigma + X_4\sigma + X_5\kappa_5 g_{5t} + X_6\kappa_6 g_{6t} + X_7\kappa_7 g_{7t} + X_8\kappa_8 g_{8t} - X_2\mu_{2t} - X_2\tau$$

$$\dot{X}_3 = X_3 + X_1\lambda_t p(1-f) + X_2\tau(1-f) + X_2(1-m)\lambda_t p(1-f) + X_5\kappa_5(1-g_{5t})(1-v) + X_6\kappa_6(1-g_{6t})(1-v) + X_5\delta_{5t} + X_6\delta_{6t} - X_3\mu_{3t} - X_3\sigma - X_3\alpha - X_3\gamma_{Dt}y_{D3t}(1-h_{D3t}) - X_3\gamma_{Nt}y_{N3t}(1-h_{N3t})$$

$$\dot{X}_4 = X_4 + X_1\lambda_t pf + X_2\tau f + X_2(1-m)\lambda_t pf + X_7\kappa_7(1-g_{7t})(1-v) + X_8\kappa_8(1-g_{8t})(1-v) + X_7\delta_{7t} + X_8\delta_{8t} - X_4\mu_{4t} - X_4\sigma + X_3\alpha - X_4\gamma_{Dt}y_{D4t}(1-h_{D4t}) - X_4\gamma_{Nt}y_{N4t}(1-h_{N4t})$$

$$\dot{X}_5 = X_5 + X_3\gamma_{Dt}y_{D3t}(1-h_{D3t}) - X_5\kappa_5\big(1-v(1-g_{5t})\big) - X_5\mu_{5t} - X_5\delta_{5t}$$

$$\dot{X}_6 = X_6 + X_3\gamma_{Nt}y_{N3t}(1-h_{N3t}) - X_6\kappa_6\big(1-v(1-g_{6t})\big) - X_6\mu_{6t} - X_6\delta_{6t}$$

$$\dot{X}_7 = X_7 + X_4\gamma_{Dt}y_{D4t}(1-h_{D4t}) - X_7\kappa_7\big(1-v(1-g_{7t})\big) - X_7\mu_{7t} - X_7\delta_{7t}$$

$$\dot{X}_8 = X_8 + X_4\gamma_{Nt}y_{N4t}(1-h_{N4t}) - X_8\kappa_8\big(1-v(1-g_{8t})\big) - X_8\mu_{8t} - X_8\delta_{8t}$$

The total population is given by

$$N = \sum_{i=1}^{8} X_i$$

Individuals enter the model in the susceptible state (X_1), where they face a time-varying risk of TB infection. Formally, the force of infection, λ_t, describes the hazard rate (at time t) by which a susceptible individual acquires TB. The population is assumed to mix randomly with density-independent contact rates, so transmission is modelled as frequency-dependent. The force of infection allows for varying infectivity across different categories of disease, and for temporal trends in contact rates, which yields the following formulation in the simple case of a single circulating TB strain:

$$\lambda_t = \sum_i \frac{X_i}{N} \beta_t q_i$$

where β_t is the transmission parameter for those with untreated, smear-positive, active disease at time t, and q_i is the infectivity of individuals in core state i relative to those with untreated, smear-positive active disease.

Upon infection, individuals progress either directly to active disease or to latent infection. Individuals with latent infection may subsequently progress to active TB, or they may be re-infected at a rate that is subject to the partial immunity conferred by an existing infection. Active disease is categorized as smear-positive or smear-negative. Smear-negative cases may progress to smear-positive, and all individuals with active disease may spontaneously self-cure, which returns them to the latent/recovered state. An individual with active disease can be diagnosed as a TB case, according to the characteristics of the diagnostic algorithm, and initiated on treatment. Treatment may be provided either through the national TB control program (DOTS), or through providers outside of the national program (non-DOTS). Treated individuals may complete treatment, default (returning to active disease) or die. Those who complete treatment are categorized as failures (returning to active disease) or cures (returning to the latent/recovered state). In addition to these transitions, all individuals in the core model are subject to a background mortality rate which is updated in each time step based on demographic data for each country, and to TB-related mortality specific to each active disease state.

2.2 Transitions between HIV subdivisions

Rates of transition from one HIV subdivision to another are based on estimates of HIV incidence, disease progression and treatment initiation (see Section 3.2.3 and Table 2). These rates are assumed independent of core TB states and other subdivisions. HIV incidence is modeled as a transition from the HIV-negative category to the HIV-positive, CD4 count >350 cells/μL category, with time-varying incidence rates defined as exogenous model parameters. HIV-positive individuals not on ART may progress over time to lower CD4 counts. Untreated HIV-positive individuals transition onto ART at rates specific to CD4 count category, which are allowed to vary over time to capture changing eligibility criteria and coverage of testing and referral. HIV-related mortality occurs at rates specific to each subdivision. Certain parameters governing the natural history of TB vary with respect to HIV status, as indicated in Table 2.

2.3 Transitions between drug resistance subdivisions

Transitions between TB strain subdivisions occur through infection, superinfection and acquired resistance. First, we elaborate the specification for the force of infection to allow for multiple circulating strains distinguished by their drug resistance profiles. Individuals may be infected by any of the five types of strains. When calculating the force of infection for a particular strain (λ_s for strain s) we allow for differential fitness across strains, for example indicating lower transmissibility among drug resistant vs. drug sensitive strains. The total force of infection (λ) equals the sum across the five strain-specific forces of infection (λ_s). The general formulation for the force of infection is thus given by:

$$\lambda_t = \sum_s \sum_i \frac{X_{is}}{N} \beta_t q_i \,(1 - r_s)$$

where r_s is the relative reduction in fitness for strain *s* compared to the corresponding pan-sensitive strain. An individual in the susceptible state who is newly infected with TB transitions to the subdivision of the infecting strain. An individual with latent TB who is superinfected by a different strain transitions to the subdivision of the superinfecting strain. Following Lipsitch et al. [20], we allow for superinfection by the same strain in order to preserve model neutrality with respect to strain distribution.

Individuals may also develop acquired drug resistance during TB treatment, such that individuals with pan-sensitive TB can develop mono-INH resistance, mono-RIF resistance, or MDR-TB directly. Individuals with mono-INH or mono-RIF can develop MDR-TB, and individuals with MDR-TB can develop MDR+/XDR-TB. Cases of acquired resistance arise as individuals default from or fail treatment, with rates of acquiring resistance specified for each combination of current strain and specific treatment regimen (Appendix Table 2).

2.4 Transitions between treatment history subdivisions

Individuals enter the model in the treatment-naïve category. Treatment-naïve individuals move into the treatment-experienced category upon the first transition out of any of the TB treatment states (X_5, X_6, X_7 or X_8) in the core model.

3 Model parameterization

3.1 Initialization

The model was used to estimate TB prevalence and incidence starting in 1950 onwards, with this long historical projection allowing the simulation of a realistic TB epidemic as well as providing prevalence and incidence estimates for the recent past to compare to independent data in the calibration procedure. First, we simulated a virgin epidemic, in which one infectious source case is introduced into a population of susceptibles. This epidemic was run to equilibrium, which was assumed to represent the starting conditions in 1950. The model was then run from 1950 through the end of 2011 to produce a historical time trend in TB epidemiology, with time-varying parameter values capturing changes in birth rates, background mortality rates, TB contact rates, access to TB and HIV treatment interventions, and treatment success and default rates.

3.2 Parameter values and ranges

Appendix Table 2 summarizes estimates and ranges for all model parameters. Following is a description of key data sources used to derive these values and ranges.

3.2.1 Demographics

Demographic inputs were estimated separately for each country. Historical estimates for mortality excluding HIV were obtained from the World Health Organization (unpublished data), and future background mortality was held constant at current values. Historical estimates and future projections for population growth were obtained from the United Nations Population Division [21].

3.2.2 TB epidemiology, diagnosis and treatment

Estimates for transition rates between TB-related health states were drawn from the literature and chosen to be consistent with prior TB modeling work [3-5,22-24]. ART delays the immunosuppression associated with HIV thereby reducing the effect of HIV on TB disease progression. We operationalized this as an ART effectiveness parameter (z); the values of TB natural history parameters for individuals on ART were calculated as weighted sums of parameter values for HIV-negative and untreated HIV-positives, with weights z and $(1-z)$ respectively.

Individuals receiving TB treatment were assumed to have reduced infectiousness compared to untreated individuals, with the reduction in infectiousness approximated as 1 minus the failure probability for each regimen/strain pair. Diagnostic algorithms were based on current practice and on WHO guidelines for Xpert implementation [25]. Values for the sensitivity and specificity for each diagnostic test were derived from the published literature [26-28]. As the model distinguishes between smear-negative and smear-positive TB the sensitivity of smear was defined as 0% and 100%, respectively, for these two groups. As sputum culture is considered the gold standard for diagnosis the sensitivity of this test was assumed to be 100%. Few data are available on the percentage of individuals testing negative on smear microscopy who subsequently have this diagnosis confirmed by sputum culture. Dowdy et al. [23] estimated this percentage as 5% and 37% for treatment-naïve and treatment-experienced individuals, respectively, based on 2004 South

African data. It is likely that access to sputum coverage will have risen since then, and we assumed starting values for these parameters of 20% and 80% respectively. In addition, 80% of individuals who are diagnosed positive with a history of prior treatment were assumed to receive DST.

Parameters relating to treatment program coverage and performance were based on routine monitoring data aggregated by the WHO Stop TB Department [29]. Access to DOTS TB programs (parameterized as the rate at which those with active TB attend a health center providing TB diagnosis and treatment) was estimated from reported trends in the case detection rate (CDR). First, a simple time trend was fit to national CDR data using a logistic regression model (see Appendix Figure 1). As the CDR more closely approximates a probability rather than a rate, we transformed the predicted CDR ($\widehat{CDR}$) to calculate the attendance rate (whereby rate = $1 - e^{-\widehat{CDR}}$). For the pre-1990 period, the rate of attendance for DOTS diagnosis was assumed to increase from zero to the 1990 value over a 4-year period. For future years the attendance rate was held constant at the most recent value for which data were available. The imperfections of the CDR as a measure of the probability of detection are well understood [30], and this uncertainty was reflected in the analysis by assuming a wide prior distribution for the attendance rate, with a range spanning from zero to two times the point estimate. There is little information on non-DOTS diagnosis, but this was assumed to start earlier (1970) and to continue at a low level in the future (rate of 0.2 per year, also varying within a range spanning zero to two times the point estimate). The volume of non-DOTS care was calibrated to produce observed drug resistance levels.

Rates of treatment default were based on reported program outcomes [29] for each country and calculated in a similar fashion to the attendance rate, by fitting a simple time trend to the national program data using a logistic regression model (Appendix Figure 1), and transforming the estimated probability of default to obtain the annualized default rate. TB-specific excess mortality rates were assumed to persist for the first two months of treatment before dropping to zero, and the treatment mortality rates produced by this assumption were consistent with reported program outcomes.

The probability of treatment success (probability of cure or completion among all individuals finishing a treatment regimen) will be determined by the appropriateness of the drug regimen as well as other characteristics of the treatment program—such as quality of adherence support—which might change over time. To capture the influence of these other program characteristics we assumed that the effectiveness of the first-line regimen in pan-sensitive TB was equivalent to the fraction of all individuals cured or completing treatment estimated from national program data. This was operationalized as a time trend fit to the observed data in a logistic regression model (Appendix Figure 1). The probabilities of treatment success for other strain-regimen combinations were assumed to be fixed proportions of this value, shown in Appendix Table 2.

It is assumed that diagnosis and treatment was more rudimentary in the early years of TB control programs. This assumption was operationalized in the model as a linear increase in the availability of culture, DST, and second-line regimens over the last 20 years, from an initial scenario in which there was no access to advanced tests or second line regimens.

Little information is available to estimate rates of acquired resistance by regimen and initial strain. We based our estimates on data reported in Lew et al. [31], adjusted for the prevalence of resistance to other first-line drugs (streptomycin, ethambutol) not tracked in the model (values shown in Appendix Table 2).

3.2.3 HIV epidemiology and treatment

Estimates for HIV incidence and ART coverage were obtained from UNAIDS (unpublished data). For future years, HIV incidence was assumed to decline at an exponential rate estimated from the last 7 years of incidence data. Untreated HIV-positive individuals in the model transition onto ART at rates calculated to match national reporting data on ART program scale-up. ART coverage (the fraction of eligible individuals receiving ART) was assumed to increase from current levels to the WHO universal access target of 80% coverage [32] over the course of 10 years. For Botswana, which was providing ART to over 83% of those in need by 2009, coverage was maintained at current levels. Early HIV treatment guidelines suggested a CD4 count criterion of <200 cells/μL for initiating ART [33], while recent revisions to the guidelines have raised this CD4 count criterion to <350 cells/μL [34]. For this reason all ART initiations prior to 2010 were assumed to come from the CD4 count <200 cells/μL group, and for 2010 onwards the fraction of HIV initiations coming from the CD4 count 200–350 cells/μL group was assumed to rise such that by 2015 individuals in the CD4 count 200–350 and <200 cells/μL groups would have equal probability of initiation on ART. Estimates for HIV-specific mortality rates (with and without ART) were drawn from the literature [35-40].

3.2.4 Resource use and costs

Costs were assessed from a health system perspective and expressed in 2011 US dollars. Costs reflected resources used to deliver TB diagnosis and treatment, as provided by both public and private providers, and those used in providing ART to HIV-infected individuals. An ingredients approach to costing was used, by which the total cost to provide a particular diagnostic procedure or a course of treatment was calculated as the number of units of each specific type of resource input needed to deliver the service, multiplied by the unit cost of each resource input.

Average costs for each type of service are shown in Appendix Table 2. Unit costs for service delivery (excluding Xpert) were calculated as the average of values reported in the literature, after adjustment for inflation and differences in price levels. These adjustments were undertaken by (i) inflating values to 2011 prices using the GDP deflator in the country in which the data were derived, then (ii) adjusting for price levels between countries using per-capita GDP as a price index and (iii) converting to US dollars based on market exchange rates. Treatment costs for TB and HIV included drugs, clinic visits and monitoring tests, as well as inpatient care for individuals receiving treatment for MDR-TB. Drug costs were derived from average prices reported to the WHO price reporting mechanism [41]. Quantities of treatment monitoring visits and laboratory tests (including monitoring smears and cultures) followed a previous global analysis [1]. The cost of clinic visits associated with TB diagnosis was based on the cost of a 10-minute outpatient clinic visit as reported for each country by the WHO-CHOICE project, and the cost of a clinic visit during TB treatment based on the cost of a short (<5 minute) outpatient clinic visit from the same source. Inpatient care for MDR-TB treatment was assumed to last for 4 months, with the cost per inpatient

day estimated from the WHO-CHOICE data. For Xpert, limited data are available on the per-test cost of providing the test in routine programmatic settings, although information reported in WHO implementation guidance suggests an economic cost of US$25-35 in southern Africa (including consumables, equipment, personnel, transport, facilities and managerial overheads), and a recent costing study in South Africa suggested a per-test cost of US$26-US$36 in the national program [42]. As the per-test cost of Xpert is of interest to decision-makers and may be sensitive to negotiation, results were calculated and reported separately for three values for the Xpert per-test cost: US$20, US$30 and US$40.

3.2.5 Other parameters

Disability weights were derived from estimates published by the Global Burden of Disease study [43,44]. Published disability weights generally only cover individual conditions, and so to calculate disability weights for comorbid TB-HIV states we assumed a multiplicative functional form, whereby the combined weight was equal to one minus the product of one minus the disability weight for each of the individual conditions [45,46]. An annual discount rate of 3% was applied to all future costs and benefits included in the cost-effectiveness analysis. This value was varied between 0 and 10% in univariate sensitivity analyses.

Appendix Table 2: Base-case parameter values and ranges.

Description	Base-case value	Range*	Source
Parameters related to η_t			
New entrants at time t	Time-varying	—	[21]
Parameters related to λ_t			
Transmission parameter for individuals with (pan-sensitive) smear-positive TB in 1950 (β_{1950})	11.0	[8.3-14.3]	Mean value chosen to produce plausible value on burn-in
Annual percentage decline in transmission parameter	0.7%	[0.2%-1.6%]	[4]
Infectivity of smear-negative TB, relative to smear-positive TB (q_i)	0.22	[0.12-0.37]	[3]
Fitness cost for drug-resistant TB strains (r_s):			[5,22,47,48]
Mono-INH resistant	0.05	[0.03-0.08]	
Mono-RIF resistant	0.15	[0.08-0.23]	
MDR-TB	0.27	[0.15-0.42]	
MDR+ / XDR-TB	0.27	[0.15-0.42]	
Parameters related to γ_{Dt} and γ_{Nt}			
Rate of attending TB testing site, for individuals with active TB	Time-varying	0-200% of base-case value	Trend estimated from country program data 1990-2011 [29]
Rate ratio of attending TB testing, for individuals without active TB compared to those with active TB	0.015	[0.009-0.023]	Calibrated to observed ratio of TB testing to TB notifications [29]
Parameters related to y_{Dit} and y_{Nit}			
Sensitivity of sputum smear microscopy:			Assumed
Smear-negative TB	0.0	—	
Smear-positive TB	1.0	—	
Specificity of sputum smear microscopy	0.974	[0.965-0.982]	[27]

Description	Base-case value	Range*	Source
Sensitivity of sputum culture	1.0	—	Assumed
Specificity of sputum culture	0.984	[0.978-0.989]	[28]
Sensitivity of Xpert for TB:			[26]
Smear-negative TB	0.725	[0.655-0.788]	
Smear-positive TB	0.982	[0.969-0.991]	
Specificity of Xpert for TB	0.992	[0.982-0.997]	[26]
Sensitivity of Xpert for RIF resistance	0.976	[0.946-0.992]	[26]
Specificity of Xpert for RIF resistance	0.981	[0.966-0.990]	[26]
Probability of sputum culture following a negative sputum smear (status quo algorithm):			[23]
Treatment-naïve patients	0.20	[0.11-0.31]	
Treatment-experienced patients	0.80	[0.69-0.89]	
Probability of DST following a positive TB diagnosis (status quo algorithm):			[23]
Treatment-naïve patients	0.00	—	
Treatment-experienced patients	0.80	[0.69-0.89]	
Sensitivity of clinical diagnosis	0.209	[0.12-0.33]	[49]
Specificity of clinical diagnosis	0.953	[0.92-0.97]	[49]
Parameters related to h_{Dit} and h_{Nit}			
Probability of loss to follow-up between initial presentation and treatment initiation:			[24]
With prompt diagnosis (smear, Xpert)	0.15	[0.09 – 0.24]	
With delayed diagnosis (culture, DST)	0.25	[0.14 – 0.39]	
Parameters related to δ_{it}			
Treatment default rate, DOTS	Time-varying	50-150% of point estimate	Trend estimated from country program data 1990-2011 [29]
Treatment default rate, non-DOTS	0.58	[0.27-0.85]	[27]
Parameters related to g_{it}			
Probability of treatment success, for individuals with pan-sensitive TB completing first-line regimen	Time-varying	50-150% of point estimate	Trend estimated from country program data 1990-2011 [29]
Risk ratio of treatment success, relative to pan-sensitive TB treated with first-line DOTS regimen:			[50-54]
First-line regimen, partially-sensitive strain	0.83	[0.73-0.90]	
First-line regimen, non-sensitive strain	0.44	[0.23-0.67]	
Second-line regimen, sensitive strain	0.93	[0.89-0.96]	
Second-line regimen, non-sensitive strain	0.44	[0.23-0.67]	
Non-DOTS regimen, non-MDR strain	0.73	[0.58-0.85]	
Non-DOTS regimen, MDR strain	0.44	[0.23-0.67]	
Parameters related to μ_{it}			
Background mortality rate, (ages 15+)	Time-varying	—	WHO unpublished data
Excess mortality rate for active TB (μ_{TB}):			[4]
Smear-negative	0.21	[0.18 – 0.25]	
Smear-positive	0.30	[0.21 – 0.41]	
Excess mortality rate for HIV (μ_{HIV}):			[35-40]
CD4 >350 cells/μl, no ART	0. 008	[0.005-0.012]	
CD4 200–350 cells/μl, no ART	0.030	[0.018-0.048]	
CD4 <200 cells/μl, no ART	0.230	[0.136-0.366]	
On ART initiated at CD4 >350 cells/μl	0.008	[0.005-0.012]	
On ART initiated at CD4 200-350 cells/μl	0.023	[0.014-0.037]	
On ART initiated at CD4 <200 cells/μl	0.050	[0.031-0.076]	

Description	Base-case value	Range*	Source
Excess mortality rate for advanced HIV (CD4 <200) and active TB without ART ($\mu_{TB\text{-}HIV}$)	0.80	[0.472-1.272]	[14,15]
Parameters related to m			
Partial immunity afforded by prior infection:			[2,3,5,47]
HIV-negative	0.65	[0.37-0.87]	
HIV-positive, CD4 >350 cells/μl, no ART	0.45	[0.23-0.68]	
HIV-positive, CD4 200–350 cells/μl, no ART	0.25	[0.14-0.39]	
HIV-positive, CD4 <200 cells/μl, no ART	0.25	[0.14-0.39]	
Parameters related to p			
Probability of fast breakdown to active TB, for new infections:			[4,55]
HIV-negative	0.115	[0.09-0.14]	
HIV-positive, CD4 >350 cells/μl, no ART	0.33	[0.18-0.51]	
HIV-positive, CD4 200–350 cells/μl, no ART	0.67	[0.49-0.82]	
HIV-positive, CD4 <200 cells/μl, no ART	0.94	[0.70-1.00]	
Parameters related to f			
Probability of smear-positivity, for incident TB cases:			[3,11,55]
HIV-negative	0.62	[0.42-0.80]	
HIV-positive, CD4 >350 cells/μl, no ART	0.45	[0.23-0.68]	
HIV-positive, CD4 200–350 cells/μl, no ART	0.35	[0.19-0.54]	
HIV-positive, CD4 <200 cells/μl, no ART	0.35	[0.19-0.54]	
Parameters related to τ			
Rate of breakdown from latent/recovered to active TB (per 100,000):			[3,4,56]
HIV-negative	0.001	[0.0003-0.0024]	
HIV-positive, CD4 >350 cells/μl, no ART	0.003	[0.001-0.006]	
HIV-positive, CD4 200–350 cells/μl, no ART	0.085	[0.060-0.130]	
HIV-positive, CD4 <200 cells/μl, no ART	0.170	[0.100-0.270]	
Parameters related to α			
Rate of conversion from smear-negative to smear-positive active TB	0.015	[0.010-0.023]	[4]
Parameters related to σ			
Rate of self-cure for active TB:			[4,5,57]
HIV-negative	0.2	[0.15-0.25]	
HIV-positive, CD4 >350 cells/μl, no ART	0.1	[0.06-0.16]	
HIV-positive, CD4 200–350 cells/μl, no ART	0.0	—	
HIV-positive, CD4 <200 cells/μl, no ART	0.0	—	
Parameters related to κ_i			
Duration of TB treatment ($1/\kappa_i$):			[58]
First-line	6 mo.	—	
Mono-INH resistant	9 mo.	—	
Mono-RIF resistant	18 mo.	—	
MDR-TB	21 mo.	—	
MDR+/XDR-TB	21 mo.	—	
Non-DOTS (averaged)	18 mo.	—	
Parameters related to v			
Probability that failed treatment cases are correctly identified and returned to treatment	0.5	[0.25-0.75]	Assumed
Additional parameters related to TB strain subdivisions			

**Description**	**Base-case value**	**Range***	**Source**
Rates of acquisition of TB drug resistance:			[31]
Pan-sensitive → Mono-INH resistant, first-line	0.020	[0.012-0.032]	
regimen	0.003	[0.002-0.005]	
Pan-sensitive → Mono-RIF resistant, first-line	0.010	[0.006-0.016]	
regimen	0.020	[0.012-0.032]	
Pan-sensitive → MDR-TB, first-line regimen			
Mono-RIF or Mono-INH resistant → MDR-TB, appropriate second-line regimen	0.230	[0.139-0.359]	
Mono-RIF or Mono-INH resistant → MDR-TB, inappropriate second-regimen	0.020	[0.012-0.032]	
MDR-TB → MDR+/XDR-TB, appropriate second-line regimen	0.230	[0.139-0.359]	
MDR-TB → MDR+/XDR-TB, inappropriate second-line regimen	3.0	[1.8-4.8]	
Rate ratio of acquired resistance, pan-sensitive, non-DOTS regimen			
Additional parameters related to HIV subdivisions			
HIV incidence	Time-varying	Annual change varied ±5%	UNAIDS unpublished estimates
Rate of HIV progression for individuals not on ART:			[59-62]
From CD4 >350 cells/µl to CD4 200–350 cells/µl	0.134	[0.08-0.21]	
From CD4 200–350 cells/µl to CD4 <200 cells/µl	0.505	[0.30-0.81]	
Historical ART coverage for treatment-eligible HIV-positive individuals	Time-varying	—	UNAIDS unpublished estimates
Future ART coverage for treatment-eligible HIV-positive individuals	0.8	[0.47-0.96]	[32]
Effectiveness of ART in reversing effect of HIV on TB natural history (all TB transition parameters subdivided by HIV status, excluding mortality)	0.7	[0.47-0.87]	[63-65]
Proportion of HIV negative individuals with prior HIV test result	0.5	[0.25-0.75]	[66-68]
Additional parameters related to costs and health outcomes			
Per-test cost of Xpert	$20, $30, $40	Assumed fixed	[25,69,70]
Per-test cost of smear diagnosis:			[24,71-77]
Botswana	$6.13	[4.18-8.68]	
Lesotho	$3.31	[2.26-4.68]	
Namibia	$5.31	[3.63-7.51]	
South Africa	$5.94	[4.06-8.39]	
Swaziland	$4.24	[2.90-5.99]	
Per-test cost of culture:			[24,71,73,74,77]
Botswana	$15.83	[13.07-18.99]	
Lesotho	$8.56	[7.07-10.27]	
Namibia	$13.72	[11.33-16.46]	
South Africa	$15.33	[12.66-18.39]	
Swaziland	$10.94	[9.04 -13.13]	
Per-test cost of chest X-ray:			[71,76,78]
Botswana	$16.69	[11.35-23.70]	
Lesotho	$9.03	[6.14-12.81]	
Namibia	$14.46	[9.83-20.52]	
South Africa	$16.16	[10.99-22.94]	
Swaziland	$11.54	[7.85-16.38]	
Per-test cost of drug sensitivity testing:			[79,80]
Botswana	$81.97	[61.44-107.17]	
Lesotho	$44.32	[33.22-57.94]	
Namibia	$71.02	[53.24-92.85]	
South Africa	$79.37	[59.50-103.77]	
Swaziland	$56.65	[42.47-74.07]	

**Description**	**Base-case value**	**Range***	**Source**
Cost of outpatient diagnostic visit:			[81]
Botswana	$10.32	[6.09-16.40]	
Lesotho	$2.94	[1.73-4.67]	
Namibia	$7.99	[4.71-12.70]	
South Africa	$10.30	[6.08-16.39]	
Swaziland	$6.21	[3.66-9.87]	
Cost of outpatient treatment visit:			[81]
Botswana	$6.85	[4.04-10.89]	
Lesotho	$1.95	[1.15-3.10]	
Namibia	$5.31	[3.13-8.44]	
South Africa	$6.85	[4.04-10.89]	
Swaziland	$4.13	[2.44-6.57]	
Cost of inpatient care, per day:			[81]
Botswana	$38.99	[23.00-61.99]	
Lesotho	$8.78	[5.18-13.96]	
Namibia	$28.76	[16.97-45.73]	
South Africa	$39.38	[23.23-62.61]	
Swaziland	$21.91	[12.93-34.84]	
Monthly TB regimen cost:			[41]
First-line	$5.86	[3.46-9.32]	
Mono-INH resistant	$18.02	[10.63-28.65]	
Mono-RIF resistant	$33.91	[20.01-53.92]	
MDR-TB	$119.37	[70.43-189.79]	
MDR+/XDR-TB	$179.06	[105.64-284.70]	
Monthly frequency of treatment activities, averaged over treatment course:			[1]
Clinic visits (first-line)	5.9	[3.5-9.4]	
Clinic visits (second-line)	22.3	[13.2-35.4]	
Monitoring smears (first-line)	1.0	[0.6-1.6]	
Monitoring smears (second-line)	1.0	[0.6-1.6]	
Sputum cultures (second-line)	0.43	[0.25-0.68]	
Chest X-rays (second-line)	0.14	[0.08-0.22]	
Number of months of inpatient care with MDR-TB treatment	4.0	[2.4-6.4]	[82]
Monthly cost of ART:	$104.97	[84-80-128.48]	[41,83-87]
Botswana	$69.63	[57.22-83.92]	
Lesotho	$94.68	[76.78-115.52]	
Namibia	$102.53	[82.90-125.40]	
South Africa	$81.20	[66.25-98.52]	
Swaziland			
Disability weights:			[43,44]
Active TB	0.271	[0.151-0.422]	
HIV-positive, CD4 >350 cells/μl, no ART	0.135	[0.078-0.213]	
HIV-positive, CD4 200–350 cells/μl, no ART	0.320	[0.176-0.496]	
HIV-positive, CD4 <200 cells/μl, no ART	0.505	[0.252-0.757]	
HIV-positive, ART initiated at CD4 >350 cells/μl	0.135	[0.078-0.213]	
HIV-positive, ART initiated at CD4 200–350 cells/μl	0.151	[0.087-0.238]	
HIV-positive, ART initiated at CD4 <200 cells/μl	0.167	[0.096-0.262]	
Discount rate	3.0%	[0-10%]	[88,89]

All costs are given in 2011 US dollars
* Ranges for parameters were derived from the literature where sufficient data existed, and otherwise were calculated as ± 50% of the point estimate value.

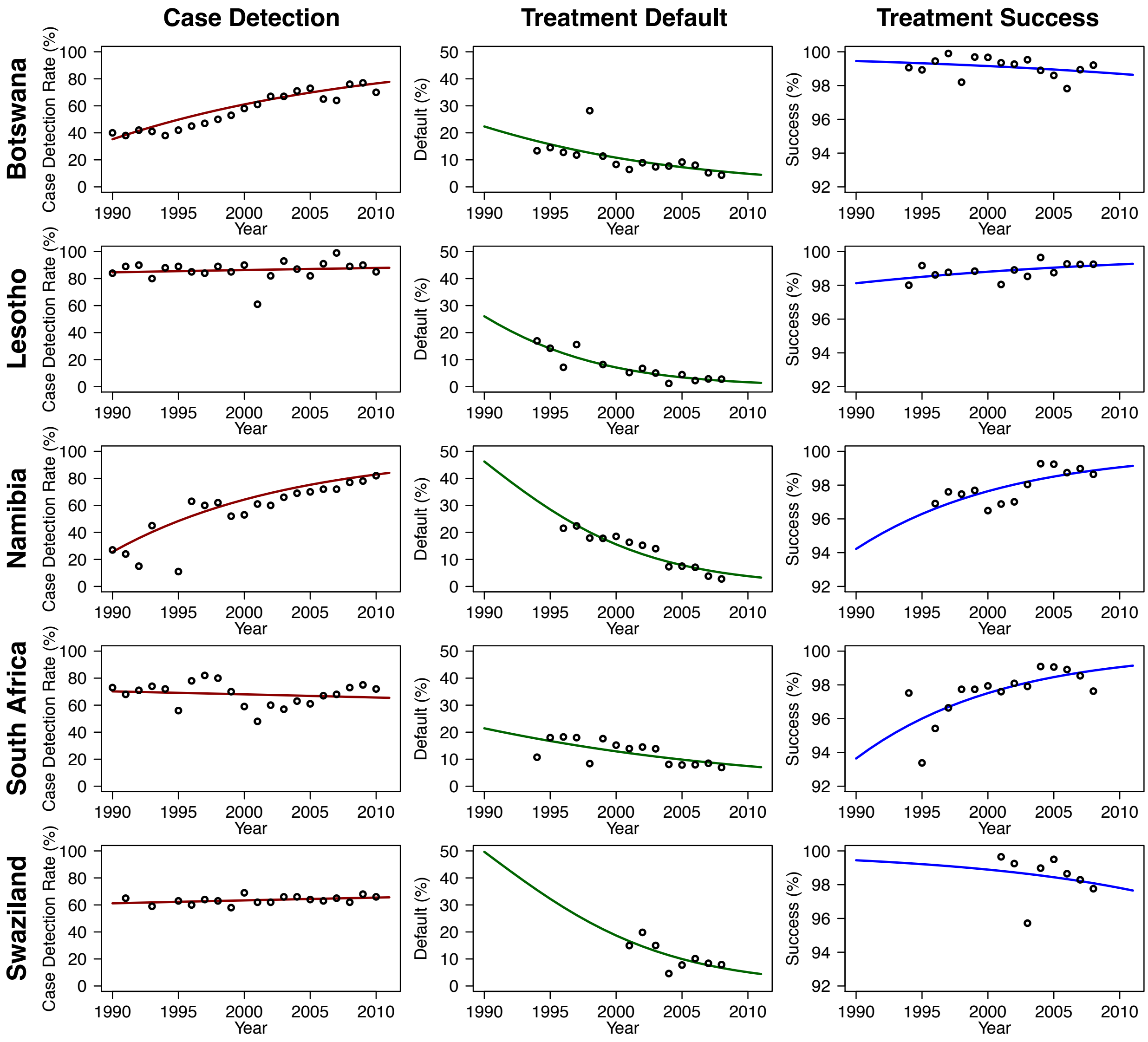

Appendix Figure 1: Time-varying parameter inputs for TB diagnosis and treatment.

4 Model calibration

We adopted a Bayesian approach to calibrate the model, following the prior work of Raftery, Alkema and colleagues [90,91]. The approach enables the synthesis of multiple sources of information on the values of model outputs, and allows for characterization of the uncertainty in model results using Bayesian posterior intervals and similar metrics.

The disease model (M) can be considered a deterministic mapping from the parameter space of the model inputs ($\mathbf{\Theta}$) to that of the model outputs ($\mathbf{\Phi}$), such that $M\colon \theta \rightarrow \varphi$. For some of these outputs (φ_1) we have external data (X) related to φ_1 through a defined probability model. An example of φ_1 would be model projections of MDR-TB prevalence for 2010, and an example for X would be the estimate for MDR-TB prevalence obtained from a population-based survey conducted in the same year. For other outputs (φ_2) — generally those about we would like to make inferences — we have no external data, but can estimate their distribution based on the prior information about θ and φ_2, relying on the deterministic disease model to link these three sets of parameters. As we have probabilistic prior information on θ and φ_1, we can use this information to estimate the posterior density of θ:

$$p(\theta|X) \propto p(\theta) * L(X|\theta)$$

where $p(\theta)$ is the prior distribution of the model inputs, and $L(X|\theta)$ is the likelihood function for θ constructed with the external data X. While this likelihood function cannot be estimated directly, we can transform θ into the output parameter space to estimate the likelihood:

$$\begin{aligned} p(\theta|X) &\propto p(\theta) * L\big(X\big|M(\theta)\big) \\ &\propto p(\theta) * L(X|\varphi_1) \end{aligned}$$

Having obtained a posterior distribution for the model inputs, we can then estimate the posterior density of φ_2 through the model, as $M(p(\theta|X))$. An analytic solution can be difficult or impossible to calculate for disease models of moderate or greater complexity, but the posterior distributions can be approximated using numerical methods. Following Alkema et al. [90], we used a sampling / importance resampling (SIR) algorithm [92]:

(1) The prior uncertainty was quantified for each model parameter, expressed as the ranges given in Appendix Table 2. Each range was assumed to represent the 95% confidence interval for a log-normal distribution (for parameters defined over positive numbers, e.g. rates, costs) or logit-normal distribution (for parameters defined over the interval 0–1, e.g. probabilities, disability weights).

(2) For each country, a likelihood function was constructed to calibrate the model, based on (a) WHO estimates [29] for TB prevalence and incidence in 1990 and 2009 (the earliest and most recent estimates available, respectively); and (b) results from a country-level drug resistance survey, where available [93]. The uncertainty around prevalence and incidence estimates was assumed to be distributed normally, with a variance calculated from the width of the confidence intervals reported with the WHO estimates. The sample size and

MDR-TB prevalence reported by the drug resistance surveys were used to parameterize two beta distributions (one for treatment-experienced and one for treatment-naïve individuals), assuming a design effect of 2.0 for the survey sample. These likelihood functions were assumed to be mutually independent, and multiplied to create a joint likelihood function.

(3) For each country 20,000 random parameter sets were drawn via Latin hypercube sampling, and a separate simulation conducted for each of these parameter sets. A likelihood statistic was calculated for each of these model runs by applying the joint likelihood function to the model outputs produced by a particular parameter set.

(4) The 20,000 parameter sets from the first stage sample were then resampled with replacement to create a final array of parameter sets, using the likelihoods as sampling weights. A sample size of 100,000 was used for this second sample as this step is not computationally intensive.

(5) Results were calculated by running the model for the resampled array of parameter sets. For each quantity of interest from the model, the point estimate was calculated as the mean of the results for the second stage sample, and 95% posterior intervals (the Bayesian equivalent of confidence intervals) calculated from the 2.5th and 97.5th percentiles of the simulation results for each quantity of interest.

This procedure was conducted separately for each country. Appendix Figure 2 shows the results of the calibration for TB prevalence, incidence and MDR-TB prevalence in South Africa, overlaid with the WHO estimates and drug resistance survey data. Posterior distributions for health outcomes and costs for the southern Africa region were calculated by summing the outcomes for each country.

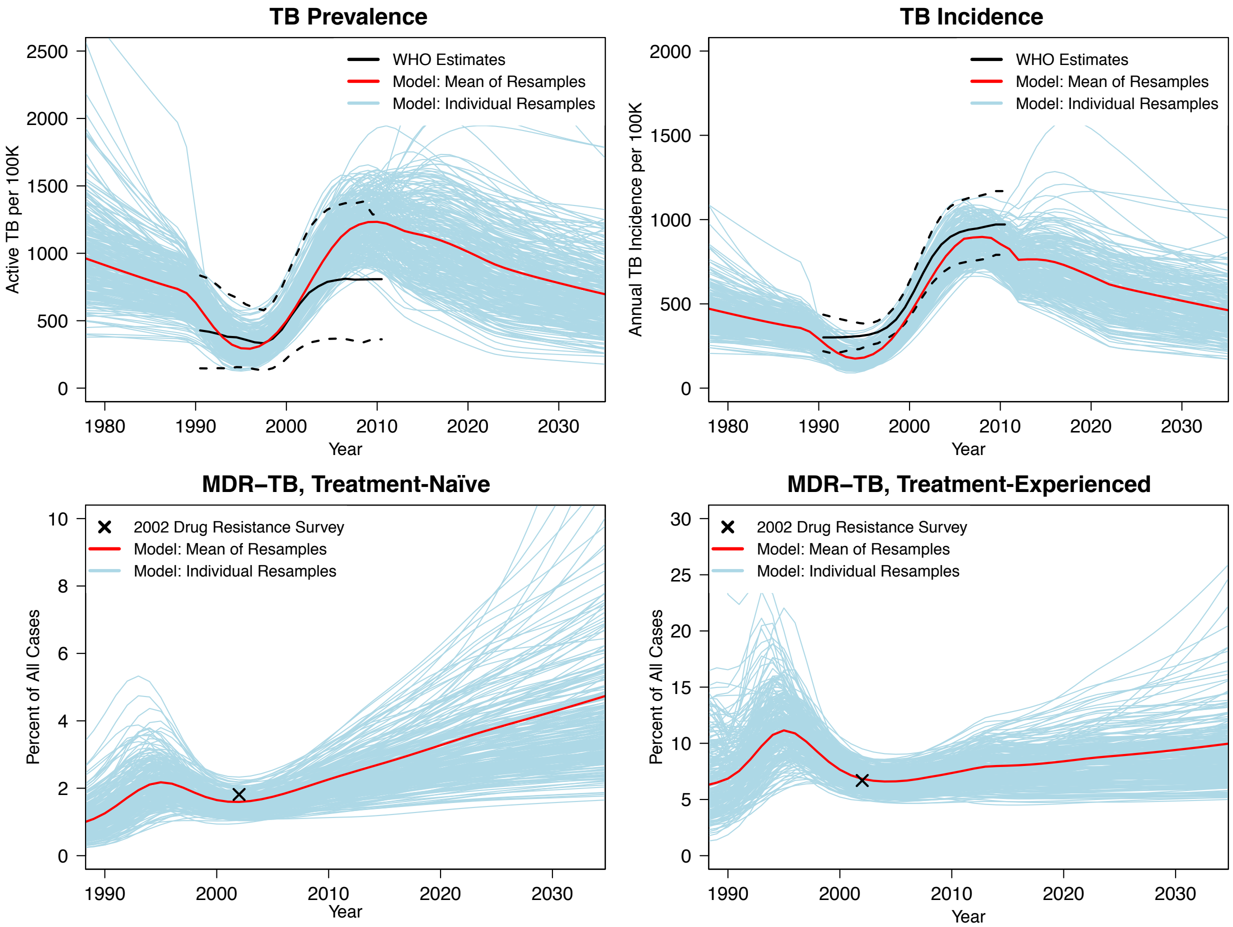

Appendix Figure 2: Calibrated outcomes for South Africa based on sampling / importance resampling.

5 Sensitivity and uncertainty analyses

We adopted four approaches to investigate the sensitivity of results to changes in model inputs.

5.1 Deterministic one-way senstitivity analyses

Traditional deterministic one-way sensitivity analyses describe how the value of a model output responds to deliberate changes in the value of a particular input parameter, when all other variables are held at their expected values. For all input parameters, we evaluated how the 10-year incremental cost-effectiveness ratio for Xpert vs. the status quo changed as each individual parameter was varied ±1 standard deviation from the mean of its posterior distribution, while all other variables were held at their posterior mean values. The resulting information represents a set of 'what-if' analyses, useful for identifying situations where the optimal policy decision might change if the value of an individual parameter were found to differ substantially from prior expectations. The main paper (see Figure 6) reported on results for the 10 most influential parameters identified through this process for South Africa. A full listing of results, by country, is shown here in Appendix Tables 3A-E.

5.2 Analysis of partial rank correlation coefficients

Partial rank correlation coefficients (PRCCs) represent a complementary approach for investigating uncertainty, providing information on the relative influence that individual parameters have on model outcomes based on the results of a probabilistic sensitivity analysis [4,94,95]. We calculated PRCCs using the resampled parameter sets produced by the calibration procedure. Results for the 10 parameters having the greatest influence on the cost-effectiveness ratio for Xpert in South Africa, under a 10-year time horizon, are shown in Figure S3 (linked from the main paper and included among the supplementary figures in Section 7 of this appendix).

Appendix Table 3A: Univariate sensitivity analysis results, Botswana (base-case ICER = US$1,289 / DALY).

Parameter description	Low parameter value	High parameter value	ICER w/ low parameter value	ICER w/ high parameter value
Transmission parameter for individuals with smear-positive TB in 1950	9.6	12.2	1,381	1,248
Annual percentage decline in transmission parameter	0.004	0.010	1,236	1,394
Infectivity of smear-negative TB, relative to smear-positive TB	0.17	0.30	1,447	1,167
Fitness cost for drug-resistant TB strains (% of base-case value)	82%	134%	1,392	1,218
Rate of attending TB testing site, for individuals with active TB, DOTS, 1990 (% of base-case value)	53%	174%	1,292	1,285
Rate of attending TB testing site, for individuals with active TB, DOTS, 2010 (% of base-case value)	90%	191%	1,071	1,561
Rate of attending TB testing site, for individuals with active TB, non-DOTS, 1990 (% of base-case value)	48%	136%	1,242	1,335
Rate of attending TB testing site, for individuals with active TB, non-DOTS, 2010 (% of base-case value)	50%	140%	1,245	1,334
Rate ratio of TB testing, for individuals without active TB compared to those with active TB	0.011	0.019	1,203	1,374
Specificity of sputum smear microscopy	0.97	0.98	1,256	1,321
Specificity of sputum culture	0.98	0.99	1,284	1,293
Sensitivity of Xpert for TB, smear-negative TB	0.69	0.76	1,314	1,267
Sensitivity of Xpert for TB, smear-positive TB	0.98	0.99	1,295	1,282
Specificity of Xpert for TB	0.99	1.00	1,316	1,261
Sensitivity of Xpert for RIF resistance	0.96	0.99	1,280	1,297
Specificity of Xpert for RIF resistance	0.98	0.99	1,291	1,286
Probability of sputum culture following a negative sputum smear (status quo algorithm), treatment-naïve patients	0.16	0.27	1,192	1,389
Probability of sputum culture following a negative sputum smear (status quo algorithm), treatment-experienced patients	0.75	0.85	1,289	1,288
Probability of DST following a positive TB diagnosis (status quo algorithm), treatment-experienced patients	0.75	0.85	1,324	1,253
Probability of loss to follow-up between initial presentation and treatment initiation, with prompt diagnosis	0.12	0.19	1,281	1,298
Probability of loss to follow-up between initial presentation and treatment initiation, with delayed diagnosis	0.18	0.30	1,343	1,238
Treatment default rate, DOTS (% of base-case value)	78%	129%	1,278	1,293
Treatment default rate, non-DOTS	0.39	0.70	1,363	1,218
Probability of treatment success, for individuals with pan-sensitive TB completing first-line regimen (% of base-case value)	77%	125%	1,304	1,283
Risk ratio of treatment success, first-line regimen, semi-sensitive strain	0.79	0.87	1,296	1,282
Risk ratio of treatment success, first-line regimen, non-sensitive strain	0.32	0.56	1,303	1,278
Risk ratio of treatment success, second-line regimen, sensitive strain	0.91	0.95	1,301	1,277
Risk ratio of treatment success, second-line regimen, non-sensitive strain	0.34	0.58	1,339	1,260
Risk ratio of treatment success, non-DOTS regimen, non-MDR strain	0.65	0.81	1,287	1,290

Parameter description	Low parameter value	High parameter value	ICER w/ low parameter value	ICER w/ high parameter value
Risk ratio of treatment success, non-DOTS regimen, MDR strain	0.32	0.56	1,302	1,277
Excess mortality rate for active TB, smear-negative	0.20	0.23	1,282	1,295
Excess mortality rate for active TB, smear-positive	0.26	0.37	1,322	1,267
Excess mortality rate for HIV, CD4 >350 cells/μl, no ART	0.006	0.010	1,285	1,292
Excess mortality rate for HIV, CD4 200-350 cells/μl, no ART	0.023	0.038	1,287	1,290
Excess mortality rate for HIV, CD4 <200 cells/μl, no ART	0.17	0.28	1,285	1,291
Excess mortality rate for HIV, on ART initiated at CD4 >350 cells/μl	0.006	0.010	1,289	1,289
Excess mortality rate for HIV, on ART initiated at CD4 200-350 cells/μl	0.017	0.028	1,286	1,291
Excess mortality rate for HIV, on ART initiated at CD4 <200 cells/μl	0.038	0.062	1,275	1,302
Excess mortality rate for advanced HIV (CD4 <200 cells/μl) and active TB without ART	0.62	1.04	1,287	1,290
TB treatment mortality rates (% of base-case values)	77%	127%	1,308	1,271
Partial immunity afforded by prior infection, HIV-negative	0.60	0.81	1,283	1,303
Partial immunity afforded by prior infection, HIV-positive, CD4 >350 cells/μl, no ART	0.35	0.58	1,291	1,287
Partial immunity afforded by prior infection, HIV-positive, CD4 200-350 cells/μl, no ART	0.19	0.31	1,288	1,290
Partial immunity afforded by prior infection, HIV-positive, CD4 <200 cells/μl, no ART	0.18	0.32	1,288	1,289
Probability of fast breakdown to active TB, with new infection, HIV-negative	0.10	0.12	1,333	1,256
Probability of fast breakdown to active TB, with new infection, HIV-positive, CD4 >350 cells/μl, no ART	0.24	0.41	1,298	1,282
Probability of fast breakdown to active TB, with new infection, HIV-positive, CD4 200-350 cells/μl, no ART	0.59	0.77	1,290	1,287
Probability of fast breakdown to active TB, with new infection, HIV-positive, CD4 <200 cells/μl, no ART	0.87	1.00	1,289	1,288
Probability of smear-positivity, for incident TB cases, HIV-negative	0.56	0.74	1,190	1,428
Probability of smear-positivity, for incident TB cases, HIV-positive, CD4 >350 cells/μl, no ART	0.34	0.57	1,262	1,317
Probability of smear-positivity, for incident TB cases, HIV-positive, CD4 200-350 cells/μl, no ART	0.27	0.44	1,272	1,307
Probability of smear-positivity, for incident TB cases, HIV-positive, CD4 <200 cells/μl, no ART	0.26	0.44	1,273	1,305
Rate of breakdown from latent/recovered to active TB, HIV-negative	0.0006	0.0014	1,339	1,251
Rate of breakdown from latent/recovered to active TB, HIV-positive, CD4 >350 cells/μl, no ART	0.002	0.004	1,296	1,281
Rate of breakdown from latent/recovered to active TB, HIV-positive, CD4 200-350 cells/μl, no ART	0.08	0.11	1,305	1,275
Rate of breakdown from latent/recovered to active TB, HIV-positive, CD4 <200 cells/μl, no ART	0.14	0.22	1,314	1,273
Rate of conversion from smear-negative to smear-positive active TB	0.012	0.019	1,286	1,291
Rate of self-cure for active TB, HIV-negative	0.19	0.24	1,258	1,321
Rate of self-cure for active TB, HIV-positive, CD4 >350 cells/μl, no ART	0.08	0.13	1,284	1,292

Parameter description	Low parameter value	High parameter value	ICER w/ low parameter value	ICER w/ high parameter value
Probability that failed treatment cases are correctly identified and returned to treatment	0.38	0.63	1,294	1,283
Rates of acquisition of TB drug resistance (% of base-case value)	77%	127%	1,179	1,414
HIV incidence trend, post-2011 (% of base-case value)	98%	103%	1,290	1,287
Rate of HIV progression for individuals not on ART, from CD4 >350 cells/µl to CD4 200-350 cells/µl	0.11	0.17	1,266	1,308
Rate of HIV progression for individuals not on ART, from CD4 200-350 cells/µl to CD4 <200 cells/µl	0.35	0.66	1,287	1,290
Future ART coverage for treatment-eligible HIV-positive individuals	0.66	0.93	1,180	1,371
Effectiveness of ART in reversing effect of HIV on TB natural history	0.54	0.75	1,213	1,393
Per-test cost of smear diagnosis	4.9	7.5	1,334	1,243
Per-test cost of culture	14.4	17.4	1,301	1,276
Per-test cost of chest X-ray	13.5	19.8	1,288	1,289
Per-test cost of drug sensitivity testing	71.0	94.5	1,296	1,281
Cost of outpatient diagnostic visit	7.7	13.1	1,321	1,256
Cost of outpatient treatment visit	5.0	9.0	1,281	1,296
Cost of inpatient care, per day	28.6	48.8	1,246	1,331
Monthly TB regimen costs (% of base-case value)	0.74	1.27	1,267	1,310
Monthly frequency of treatment activities, averaged over treatment course, clinic visits (first-line)	4.3	7.5	1,299	1,278
Monthly frequency of treatment activities, averaged over treatment course, clinic visits (second-line)	15.9	27.4	1,266	1,311
Monthly frequency of treatment activities, averaged over treatment course, monitoring smears (first-line)	0.73	1.25	1,290	1,287
Monthly frequency of treatment activities, averaged over treatment course, monitoring smears (second-line)	0.74	1.26	1,288	1,289
Monthly frequency of treatment activities, averaged over treatment course, sputum cultures (second-line)	0.31	0.54	1,288	1,290
Monthly frequency of treatment activities, averaged over treatment course, chest X-rays (second-line)	0.11	0.18	1,288	1,289
Number of months of inpatient care with MDR-TB treatment	3.0	5.1	1,246	1,331
Monthly cost of ART	93.3	116.0	1,227	1,350
Disability weight, active TB	0.20	0.34	1,366	1,219
Disability weight, HIV-positive, CD4 >350 cells/µl, no ART	0.10	0.17	1,285	1,292
Disability weight, HIV-positive, CD4 200-350 cells/µl, no ART	0.23	0.39	1,289	1,289
Disability weight, HIV-positive, CD4 <200 cells/µl, no ART	0.36	0.64	1,282	1,295
Disability weight, HIV-positive, on ART initiated at CD4 >350 cells/µl	0.10	0.17	1,278	1,300
Disability weight, HIV-positive, on ART initiated at CD4 200-350 cells/µl	0.12	0.19	1,284	1,293
Disability weight, HIV-positive, on ART initiated at CD4 <200 cells/µl	0.13	0.21	1,271	1,307
Annual discount rate	0	10%	1,265	1,348

Appendix Table 3B: Univariate sensitivity analysis results, Lesotho (base-case ICER = US$1,071 / DALY).

Parameter description	Low parameter value	High parameter value	ICER w/ low parameter value	ICER w/ high parameter value
Transmission parameter for individuals with smear-positive TB in 1950	9.7	12.4	1,281	942
Annual percentage decline in transmission parameter	0.004	0.010	896	1,294
Infectivity of smear-negative TB, relative to smear-positive TB	0.18	0.30	1,254	933
Fitness cost for drug-resistant TB strains (% of base-case value)	79%	131%	1,085	1,062
Rate of attending TB testing site, for individuals with active TB, DOTS, 1990 (% of base-case value)	58%	185%	1,045	1,090
Rate of attending TB testing site, for individuals with active TB, DOTS, 2010 (% of base-case value)	60%	139%	714	1,537
Rate of attending TB testing site, for individuals with active TB, non-DOTS, 1990 (% of base-case value)	46%	139%	1,060	1,082
Rate of attending TB testing site, for individuals with active TB, non-DOTS, 2010 (% of base-case value)	48%	152%	1,068	1,075
Rate ratio of TB testing, for individuals without active TB compared to those with active TB	0.012	0.019	931	1,212
Specificity of sputum smear microscopy	0.97	0.98	1,058	1,085
Specificity of sputum culture	0.98	0.99	1,070	1,073
Sensitivity of Xpert for TB, smear-negative TB	0.69	0.76	1,101	1,046
Sensitivity of Xpert for TB, smear-positive TB	0.98	0.99	1,077	1,067
Specificity of Xpert for TB	0.99	1.00	1,083	1,060
Sensitivity of Xpert for RIF resistance	0.97	0.99	1,070	1,073
Specificity of Xpert for RIF resistance	0.98	0.99	1,072	1,071
Probability of sputum culture following a negative sputum smear (status quo algorithm), treatment-naïve patients	0.16	0.27	937	1,227
Probability of sputum culture following a negative sputum smear (status quo algorithm), treatment-experienced patients	0.74	0.85	1,067	1,076
Probability of DST following a positive TB diagnosis (status quo algorithm), treatment-experienced patients	0.75	0.85	1,078	1,065
Probability of loss to follow-up between initial presentation and treatment initiation, with prompt diagnosis	0.11	0.18	1,060	1,085
Probability of loss to follow-up between initial presentation and treatment initiation, with delayed diagnosis	0.18	0.32	1,141	1,008
Treatment default rate, DOTS (% of base-case value)	73%	120%	1,064	1,074
Treatment default rate, non-DOTS	0.48	0.78	1,086	1,058
Probability of treatment success, for individuals with pan-sensitive TB completing first-line regimen (% of base-case value)	76%	127%	1,067	1,073
Risk ratio of treatment success, first-line regimen, semi-sensitive strain	0.79	0.87	1,073	1,070
Risk ratio of treatment success, first-line regimen, non-sensitive strain	0.36	0.58	1,071	1,072
Risk ratio of treatment success, second-line regimen, sensitive strain	0.91	0.95	1,074	1,069
Risk ratio of treatment success, second-line regimen, non-sensitive strain	0.35	0.57	1,078	1,067
Risk ratio of treatment success, non-DOTS regimen, non-MDR strain	0.66	0.81	1,066	1,077

Parameter description	Low parameter value	High parameter value	ICER w/ low parameter value	ICER w/ high parameter value
Risk ratio of treatment success, non-DOTS regimen, MDR strain	0.32	0.56	1,073	1,070
Excess mortality rate for active TB, smear-negative	0.20	0.23	1,066	1,078
Excess mortality rate for active TB, smear-positive	0.27	0.39	1,046	1,104
Excess mortality rate for HIV, CD4 >350 cells/μl, no ART	0.006	0.010	1,063	1,080
Excess mortality rate for HIV, CD4 200-350 cells/μl, no ART	0.023	0.038	1,061	1,082
Excess mortality rate for HIV, CD4 <200 cells/μl, no ART	0.16	0.25	1,021	1,114
Excess mortality rate for HIV, on ART initiated at CD4 >350 cells/μl	0.006	0.010	1,071	1,071
Excess mortality rate for HIV, on ART initiated at CD4 200-350 cells/μl	0.017	0.029	1,070	1,073
Excess mortality rate for HIV, on ART initiated at CD4 <200 cells/μl	0.039	0.061	1,054	1,089
Excess mortality rate for advanced HIV (CD4 <200 cells/μl) and active TB without ART	0.61	1.00	1,033	1,103
TB treatment mortality rates (% of base-case values)	78%	130%	1,068	1,075
Partial immunity afforded by prior infection, HIV-negative	0.62	0.82	1,033	1,111
Partial immunity afforded by prior infection, HIV-positive, CD4 >350 cells/μl, no ART	0.33	0.57	1,069	1,074
Partial immunity afforded by prior infection, HIV-positive, CD4 200-350 cells/μl, no ART	0.18	0.31	1,069	1,074
Partial immunity afforded by prior infection, HIV-positive, CD4 <200 cells/μl, no ART	0.18	0.32	1,067	1,076
Probability of fast breakdown to active TB, with new infection, HIV-negative	0.10	0.12	1,159	1,000
Probability of fast breakdown to active TB, with new infection, HIV-positive, CD4 >350 cells/μl, no ART	0.25	0.43	1,109	1,036
Probability of fast breakdown to active TB, with new infection, HIV-positive, CD4 200-350 cells/μl, no ART	0.58	0.76	1,077	1,066
Probability of fast breakdown to active TB, with new infection, HIV-positive, CD4 <200 cells/μl, no ART	0.85	1.00	1,078	1,067
Probability of smear-positivity, for incident TB cases, HIV-negative	0.53	0.72	1,043	1,120
Probability of smear-positivity, for incident TB cases, HIV-positive, CD4 >350 cells/μl, no ART	0.33	0.58	1,051	1,093
Probability of smear-positivity, for incident TB cases, HIV-positive, CD4 200-350 cells/μl, no ART	0.26	0.44	1,058	1,085
Probability of smear-positivity, for incident TB cases, HIV-positive, CD4 <200 cells/μl, no ART	0.27	0.44	1,060	1,084
Rate of breakdown from latent/recovered to active TB, HIV-negative	0.0005	0.00122	1,137	1,025
Rate of breakdown from latent/recovered to active TB, HIV-positive, CD4 >350 cells/μl, no ART	0.002	0.004	1,082	1,061
Rate of breakdown from latent/recovered to active TB, HIV-positive, CD4 200-350 cells/μl, no ART	0.08	0.11	1,099	1,048
Rate of breakdown from latent/recovered to active TB, HIV-positive, CD4 <200 cells/μl, no ART	0.15	0.24	1,140	1,025
Rate of conversion from smear-negative to smear-positive active TB	0.011	0.018	1,071	1,072
Rate of self-cure for active TB, HIV-negative	0.18	0.24	1,027	1,119
Rate of self-cure for active TB, HIV-positive, CD4 >350 cells/μl, no ART	0.07	0.13	1,065	1,078

**Parameter description**	**Low parameter value**	**High parameter value**	**ICER w/ low parameter value**	**ICER w/ high parameter value**
Probability that failed treatment cases are correctly identified and returned to treatment	0.39	0.65	1,072	1,071
Rates of acquisition of TB drug resistance (% of base-case value)	66%	108%	1,052	1,094
HIV incidence trend, post-2011 (% of base-case value)	98%	102%	1,076	1,066
Rate of HIV progression for individuals not on ART, from CD4 >350 cells/μl to CD4 200-350 cells/μl	0.11	0.18	1,092	1,066
Rate of HIV progression for individuals not on ART, from CD4 200-350 cells/μl to CD4 <200 cells/μl	0.36	0.61	1,028	1,102
Future ART coverage for treatment-eligible HIV-positive individuals	0.67	0.91	1,009	1,134
Effectiveness of ART in reversing effect of HIV on TB natural history	0.58	0.79	1,000	1,157
Per-test cost of smear diagnosis	2.7	3.8	1,091	1,052
Per-test cost of culture	7.7	9.4	1,078	1,065
Per-test cost of chest X-ray	7.2	10.6	1,071	1,072
Per-test cost of drug sensitivity testing	37.9	51.1	1,075	1,068
Cost of outpatient diagnostic visit	2.2	3.8	1,080	1,063
Cost of outpatient treatment visit	1.5	2.5	1,073	1,070
Cost of inpatient care, per day	6.6	10.9	1,068	1,075
Monthly TB regimen costs (% of base-case value)	0.75	1.24	1,064	1,079
Monthly frequency of treatment activities, averaged over treatment course, clinic visits (first-line)	4.3	7.4	1,074	1,069
Monthly frequency of treatment activities, averaged over treatment course, clinic visits (second-line)	16.9	28.0	1,069	1,074
Monthly frequency of treatment activities, averaged over treatment course, monitoring smears (first-line)	0.76	1.29	1,072	1,071
Monthly frequency of treatment activities, averaged over treatment course, monitoring smears (second-line)	0.75	1.22	1,071	1,072
Monthly frequency of treatment activities, averaged over treatment course, sputum cultures (second-line)	0.32	0.54	1,071	1,072
Monthly frequency of treatment activities, averaged over treatment course, chest X-rays (second-line)	0.10	0.18	1,071	1,072
Number of months of inpatient care with MDR-TB treatment	3.0	5.1	1,067	1,076
Monthly cost of ART	62.5	76.5	1,035	1,108
Disability weight, active TB	0.21	0.35	1,130	1,019
Disability weight, HIV-positive, CD4 >350 cells/μl, no ART	0.10	0.17	1,067	1,076
Disability weight, HIV-positive, CD4 200-350 cells/μl, no ART	0.24	0.40	1,071	1,071
Disability weight, HIV-positive, CD4 <200 cells/μl, no ART	0.36	0.64	1,064	1,079
Disability weight, HIV-positive, on ART initiated at CD4 >350 cells/μl	0.10	0.17	1,067	1,076
Disability weight, HIV-positive, on ART initiated at CD4 200-350 cells/μl	0.12	0.19	1,065	1,078
Disability weight, HIV-positive, on ART initiated at CD4 <200 cells/μl	0.12	0.21	1,053	1,091
Annual discount rate	0	10%	1,050	1,126

Appendix Table 3C: Univariate sensitivity analysis results, Namibia (base-case ICER = US$863 / DALY).

Parameter description	Low parameter value	High parameter value	ICER w/ low parameter value	ICER w/ high parameter value
Transmission parameter for individuals with smear-positive TB in 1950	10.0	12.8	955	811
Annual percentage decline in transmission parameter	0.003	0.007	803	942
Infectivity of smear-negative TB, relative to smear-positive TB	0.19	0.33	992	768
Fitness cost for drug-resistant TB strains (% of base-case value)	79%	132%	916	828
Rate of attending TB testing site, for individuals with active TB, DOTS, 1990 (% of base-case value)	52%	137%	864	862
Rate of attending TB testing site, for individuals with active TB, DOTS, 2010 (% of base-case value)	42%	96%	688	1,088
Rate of attending TB testing site, for individuals with active TB, non-DOTS, 1990 (% of base-case value)	47%	153%	839	887
Rate of attending TB testing site, for individuals with active TB, non-DOTS, 2010 (% of base-case value)	44%	132%	838	890
Rate ratio of TB testing, for individuals without active TB compared to those with active TB	0.011	0.019	786	941
Specificity of sputum smear microscopy	0.97	0.98	848	879
Specificity of sputum culture	0.98	0.99	861	866
Sensitivity of Xpert for TB, smear-negative TB	0.69	0.76	883	846
Sensitivity of Xpert for TB, smear-positive TB	0.98	0.99	870	857
Specificity of Xpert for TB	0.99	1.00	878	849
Sensitivity of Xpert for RIF resistance	0.97	0.99	859	868
Specificity of Xpert for RIF resistance	0.98	0.99	865	862
Probability of sputum culture following a negative sputum smear (status quo algorithm), treatment-naïve patients	0.15	0.25	804	928
Probability of sputum culture following a negative sputum smear (status quo algorithm), treatment-experienced patients	0.75	0.86	864	863
Probability of DST following a positive TB diagnosis (status quo algorithm), treatment-experienced patients	0.75	0.86	881	846
Probability of loss to follow-up between initial presentation and treatment initiation, with prompt diagnosis	0.12	0.19	858	870
Probability of loss to follow-up between initial presentation and treatment initiation, with delayed diagnosis	0.19	0.31	895	834
Treatment default rate, DOTS (% of base-case value)	74%	116%	858	866
Treatment default rate, non-DOTS	0.45	0.75	908	822
Probability of treatment success, for individuals with pan-sensitive TB completing first-line regimen (% of base-case value)	73%	129%	873	861
Risk ratio of treatment success, first-line regimen, semi-sensitive strain	0.79	0.87	866	861
Risk ratio of treatment success, first-line regimen, non-sensitive strain	0.36	0.60	866	861
Risk ratio of treatment success, second-line regimen, sensitive strain	0.91	0.95	869	858
Risk ratio of treatment success, second-line regimen, non-sensitive strain	0.33	0.56	877	855
Risk ratio of treatment success, non-DOTS regimen, non-MDR strain	0.67	0.81	861	865

Parameter description	Low parameter value	High parameter value	ICER w/ low parameter value	ICER w/ high parameter value
Risk ratio of treatment success, non-DOTS regimen, MDR strain	0.33	0.54	869	859
Excess mortality rate for active TB, smear-negative	0.19	0.23	851	876
Excess mortality rate for active TB, smear-positive	0.27	0.37	886	848
Excess mortality rate for HIV, CD4 >350 cells/μl, no ART	0.006	0.009	861	866
Excess mortality rate for HIV, CD4 200-350 cells/μl, no ART	0.022	0.036	861	866
Excess mortality rate for HIV, CD4 <200 cells/μl, no ART	0.16	0.26	858	870
Excess mortality rate for HIV, on ART initiated at CD4 >350 cells/μl	0.006	0.010	863	863
Excess mortality rate for HIV, on ART initiated at CD4 200-350 cells/μl	0.017	0.028	863	864
Excess mortality rate for HIV, on ART initiated at CD4 <200 cells/μl	0.037	0.061	855	873
Excess mortality rate for advanced HIV (CD4 <200 cells/μl) and active TB without ART	0.61	0.97	860	866
TB treatment mortality rates (% of base-case values)	78%	126%	871	857
Partial immunity afforded by prior infection, HIV-negative	0.61	0.83	847	885
Partial immunity afforded by prior infection, HIV-positive, CD4 >350 cells/μl, no ART	0.32	0.55	863	864
Partial immunity afforded by prior infection, HIV-positive, CD4 200-350 cells/μl, no ART	0.19	0.31	862	865
Partial immunity afforded by prior infection, HIV-positive, CD4 <200 cells/μl, no ART	0.18	0.31	861	866
Probability of fast breakdown to active TB, with new infection, HIV-negative	0.10	0.12	920	819
Probability of fast breakdown to active TB, with new infection, HIV-positive, CD4 >350 cells/μl, no ART	0.26	0.43	868	860
Probability of fast breakdown to active TB, with new infection, HIV-positive, CD4 200-350 cells/μl, no ART	0.58	0.75	864	863
Probability of fast breakdown to active TB, with new infection, HIV-positive, CD4 <200 cells/μl, no ART	0.87	1.00	864	863
Probability of smear-positivity, for incident TB cases, HIV-negative	0.54	0.72	783	981
Probability of smear-positivity, for incident TB cases, HIV-positive, CD4 >350 cells/μl, no ART	0.35	0.58	855	873
Probability of smear-positivity, for incident TB cases, HIV-positive, CD4 200-350 cells/μl, no ART	0.27	0.47	858	869
Probability of smear-positivity, for incident TB cases, HIV-positive, CD4 <200 cells/μl, no ART	0.28	0.45	860	867
Rate of breakdown from latent/recovered to active TB, HIV-negative	0.0005	0.00141	911	826
Rate of breakdown from latent/recovered to active TB, HIV-positive, CD4 >350 cells/μl, no ART	0.002	0.004	866	861
Rate of breakdown from latent/recovered to active TB, HIV-positive, CD4 200-350 cells/μl, no ART	0.08	0.12	871	857
Rate of breakdown from latent/recovered to active TB, HIV-positive, CD4 <200 cells/μl, no ART	0.15	0.25	885	850
Rate of conversion from smear-negative to smear-positive active TB	0.012	0.018	862	865
Rate of self-cure for active TB, HIV-negative	0.18	0.24	831	898
Rate of self-cure for active TB, HIV-positive, CD4 >350 cells/μl, no ART	0.08	0.13	861	865

Parameter description	Low parameter value	High parameter value	ICER w/ low parameter value	ICER w/ high parameter value
Probability that failed treatment cases are correctly identified and returned to treatment	0.37	0.64	866	861
Rates of acquisition of TB drug resistance (% of base-case value)	70%	113%	812	921
HIV incidence trend, post-2011 (% of base-case value)	97%	103%	863	864
Rate of HIV progression for individuals not on ART, from CD4 >350 cells/μl to CD4 200-350 cells/μl	0.12	0.19	860	870
Rate of HIV progression for individuals not on ART, from CD4 200-350 cells/μl to CD4 <200 cells/μl	0.35	0.61	858	868
Future ART coverage for treatment-eligible HIV-positive individuals	0.65	0.92	816	911
Effectiveness of ART in reversing effect of HIV on TB natural history	0.50	0.71	824	917
Per-test cost of smear diagnosis	4.3	6.3	886	841
Per-test cost of culture	12.5	15.2	871	856
Per-test cost of chest X-ray	11.7	17.0	863	864
Per-test cost of drug sensitivity testing	61.0	80.5	867	860
Cost of outpatient diagnostic visit	5.9	10.0	878	849
Cost of outpatient treatment visit	3.9	6.6	854	873
Cost of inpatient care, per day	20.9	35.3	845	882
Monthly TB regimen costs (% of base-case value)	0.73	1.25	850	877
Monthly frequency of treatment activities, averaged over treatment course, clinic visits (first-line)	4.4	7.2	863	864
Monthly frequency of treatment activities, averaged over treatment course, clinic visits (second-line)	16.6	28.9	852	874
Monthly frequency of treatment activities, averaged over treatment course, monitoring smears (first-line)	0.75	1.22	863	864
Monthly frequency of treatment activities, averaged over treatment course, monitoring smears (second-line)	0.76	1.25	863	864
Monthly frequency of treatment activities, averaged over treatment course, sputum cultures (second-line)	0.32	0.54	863	864
Monthly frequency of treatment activities, averaged over treatment course, chest X-rays (second-line)	0.11	0.18	863	864
Number of months of inpatient care with MDR-TB treatment	3.0	5.2	845	882
Monthly cost of ART	84.6	104.0	829	898
Disability weight, active TB	0.21	0.34	912	820
Disability weight, HIV-positive, CD4 >350 cells/μl, no ART	0.10	0.17	863	864
Disability weight, HIV-positive, CD4 200-350 cells/μl, no ART	0.25	0.40	863	863
Disability weight, HIV-positive, CD4 <200 cells/μl, no ART	0.38	0.64	861	866
Disability weight, HIV-positive, on ART initiated at CD4 >350 cells/μl	0.10	0.17	860	867
Disability weight, HIV-positive, on ART initiated at CD4 200-350 cells/μl	0.12	0.19	861	866
Disability weight, HIV-positive, on ART initiated at CD4 <200 cells/μl	0.13	0.21	854	873
Annual discount rate	0	10%	843	915

Appendix Table 3D: Univariate sensitivity analysis results, South Africa (base-case ICER = US$986 / DALY).

Parameter description	Low parameter value	High parameter value	ICER w/ low parameter value	ICER w/ high parameter value
Transmission parameter for individuals with smear-positive TB in 1950	10.4	13.2	979	1,019
Annual percentage decline in transmission parameter	0.003	0.007	1,012	983
Infectivity of smear-negative TB, relative to smear-positive TB	0.17	0.28	1,087	907
Fitness cost for drug-resistant TB strains (% of base-case value)	68%	106%	1,105	903
Rate of attending TB testing site, for individuals with active TB, DOTS, 1990 (% of base-case value)	51%	159%	1,027	958
Rate of attending TB testing site, for individuals with active TB, DOTS, 2010 (% of base-case value)	52%	126%	903	1,085
Rate of attending TB testing site, for individuals with active TB, non-DOTS, 1990 (% of base-case value)	65%	178%	918	1,055
Rate of attending TB testing site, for individuals with active TB, non-DOTS, 2010 (% of base-case value)	48%	165%	905	1,070
Rate ratio of TB testing, for individuals without active TB compared to those with active TB	0.011	0.019	959	1,013
Specificity of sputum smear microscopy	0.97	0.98	976	996
Specificity of sputum culture	0.98	0.99	985	987
Sensitivity of Xpert for TB, smear-negative TB	0.70	0.76	1,003	971
Sensitivity of Xpert for TB, smear-positive TB	0.98	0.99	994	978
Specificity of Xpert for TB	0.99	1.00	994	978
Sensitivity of Xpert for RIF resistance	0.97	0.99	979	993
Specificity of Xpert for RIF resistance	0.98	0.99	988	985
Probability of sputum culture following a negative sputum smear (status quo algorithm), treatment-naïve patients	0.15	0.27	925	1,057
Probability of sputum culture following a negative sputum smear (status quo algorithm), treatment-experienced patients	0.75	0.86	987	985
Probability of DST following a positive TB diagnosis (status quo algorithm), treatment-experienced patients	0.76	0.85	1,011	961
Probability of loss to follow-up between initial presentation and treatment initiation, with prompt diagnosis	0.11	0.20	987	987
Probability of loss to follow-up between initial presentation and treatment initiation, with delayed diagnosis	0.20	0.32	1,014	960
Treatment default rate, DOTS (% of base-case value)	67%	119%	983	986
Treatment default rate, non-DOTS	0.37	0.65	1,083	896
Probability of treatment success, for individuals with pan-sensitive TB completing first-line regimen (% of base-case value)	71%	125%	1,025	973
Risk ratio of treatment success, first-line regimen, semi-sensitive strain	0.79	0.88	993	980
Risk ratio of treatment success, first-line regimen, non-sensitive strain	0.32	0.54	1,029	959
Risk ratio of treatment success, second-line regimen, sensitive strain	0.91	0.95	998	975
Risk ratio of treatment success, second-line regimen, non-sensitive strain	0.32	0.56	1,041	950
Risk ratio of treatment success, non-DOTS regimen, non-MDR strain	0.67	0.81	985	987

Parameter description	Low parameter value	High parameter value	ICER w/ low parameter value	ICER w/ high parameter value
Risk ratio of treatment success, non-DOTS regimen, MDR strain	0.33	0.55	1,016	961
Excess mortality rate for active TB, smear-negative	0.19	0.23	983	989
Excess mortality rate for active TB, smear-positive	0.26	0.37	1,072	922
Excess mortality rate for HIV, CD4 >350 cells/μl, no ART	0.006	0.010	985	987
Excess mortality rate for HIV, CD4 200-350 cells/μl, no ART	0.021	0.036	986	986
Excess mortality rate for HIV, CD4 <200 cells/μl, no ART	0.16	0.26	993	980
Excess mortality rate for HIV, on ART initiated at CD4 >350 cells/μl	0.006	0.010	986	986
Excess mortality rate for HIV, on ART initiated at CD4 200-350 cells/μl	0.018	0.029	986	986
Excess mortality rate for HIV, on ART initiated at CD4 <200 cells/μl	0.040	0.063	988	984
Excess mortality rate for advanced HIV (CD4 <200 cells/μl) and active TB without ART	0.60	1.02	983	987
TB treatment mortality rates (% of base-case values)	73%	125%	1,012	962
Partial immunity afforded by prior infection, HIV-negative	0.58	0.81	1,044	967
Partial immunity afforded by prior infection, HIV-positive, CD4 >350 cells/μl, no ART	0.33	0.54	988	985
Partial immunity afforded by prior infection, HIV-positive, CD4 200-350 cells/μl, no ART	0.18	0.30	985	987
Partial immunity afforded by prior infection, HIV-positive, CD4 <200 cells/μl, no ART	0.19	0.32	985	987
Probability of fast breakdown to active TB, with new infection, HIV-negative	0.10	0.13	988	997
Probability of fast breakdown to active TB, with new infection, HIV-positive, CD4 >350 cells/μl, no ART	0.27	0.42	986	988
Probability of fast breakdown to active TB, with new infection, HIV-positive, CD4 200-350 cells/μl, no ART	0.58	0.76	984	988
Probability of fast breakdown to active TB, with new infection, HIV-positive, CD4 <200 cells/μl, no ART	0.88	1.00	985	987
Probability of smear-positivity, for incident TB cases, HIV-negative	0.57	0.74	876	1,134
Probability of smear-positivity, for incident TB cases, HIV-positive, CD4 >350 cells/μl, no ART	0.37	0.62	956	1,020
Probability of smear-positivity, for incident TB cases, HIV-positive, CD4 200-350 cells/μl, no ART	0.28	0.46	973	1,000
Probability of smear-positivity, for incident TB cases, HIV-positive, CD4 <200 cells/μl, no ART	0.28	0.48	970	1,003
Rate of breakdown from latent/recovered to active TB, HIV-negative	0.0005	0.00126	991	983
Rate of breakdown from latent/recovered to active TB, HIV-positive, CD4 >350 cells/μl, no ART	0.002	0.004	987	985
Rate of breakdown from latent/recovered to active TB, HIV-positive, CD4 200-350 cells/μl, no ART	0.08	0.11	984	988
Rate of breakdown from latent/recovered to active TB, HIV-positive, CD4 <200 cells/μl, no ART	0.14	0.23	979	995
Rate of conversion from smear-negative to smear-positive active TB	0.012	0.018	984	988
Rate of self-cure for active TB, HIV-negative	0.18	0.23	975	998
Rate of self-cure for active TB, HIV-positive, CD4 >350 cells/μl, no ART	0.08	0.12	983	989

**Parameter description**	**Low parameter value**	**High parameter value**	**ICER w/ low parameter value**	**ICER w/ high parameter value**
Probability that failed treatment cases are correctly identified and returned to treatment	0.38	0.62	994	978
Rates of acquisition of TB drug resistance (% of base-case value)	85%	123%	888	1,094
HIV incidence trend, post-2011 (% of base-case value)	98%	103%	986	987
Rate of HIV progression for individuals not on ART, from CD4 >350 cells/μl to CD4 200-350 cells/μl	0.11	0.17	960	1,005
Rate of HIV progression for individuals not on ART, from CD4 200-350 cells/μl to CD4 <200 cells/μl	0.37	0.63	980	990
Future ART coverage for treatment-eligible HIV-positive individuals	0.67	0.93	941	1,026
Effectiveness of ART in reversing effect of HIV on TB natural history	0.60	0.80	978	994
Per-test cost of smear diagnosis	4.8	7.0	998	974
Per-test cost of culture	14.2	17.1	990	982
Per-test cost of chest X-ray	13.6	19.3	986	986
Per-test cost of drug sensitivity testing	66.7	89.5	989	983
Cost of outpatient diagnostic visit	7.9	12.6	997	975
Cost of outpatient treatment visit	5.0	8.3	952	1,020
Cost of inpatient care, per day	28.9	49.9	938	1,034
Monthly TB regimen costs (% of base-case value)	0.79	1.27	961	1,011
Monthly frequency of treatment activities, averaged over treatment course, clinic visits (first-line)	4.3	7.5	977	995
Monthly frequency of treatment activities, averaged over treatment course, clinic visits (second-line)	16.0	27.9	956	1,016
Monthly frequency of treatment activities, averaged over treatment course, monitoring smears (first-line)	0.77	1.21	985	987
Monthly frequency of treatment activities, averaged over treatment course, monitoring smears (second-line)	0.75	1.18	985	987
Monthly frequency of treatment activities, averaged over treatment course, sputum cultures (second-line)	0.32	0.52	985	987
Monthly frequency of treatment activities, averaged over treatment course, chest X-rays (second-line)	0.11	0.17	986	986
Number of months of inpatient care with MDR-TB treatment	3.0	4.7	945	1,027
Monthly cost of ART	91.9	114.0	943	1,029
Disability weight, active TB	0.20	0.32	1,037	940
Disability weight, HIV-positive, CD4 >350 cells/μl, no ART	0.10	0.17	983	989
Disability weight, HIV-positive, CD4 200-350 cells/μl, no ART	0.23	0.38	986	986
Disability weight, HIV-positive, CD4 <200 cells/μl, no ART	0.38	0.63	982	990
Disability weight, HIV-positive, on ART initiated at CD4 >350 cells/μl	0.11	0.17	983	989
Disability weight, HIV-positive, on ART initiated at CD4 200-350 cells/μl	0.12	0.20	982	990
Disability weight, HIV-positive, on ART initiated at CD4 <200 cells/μl	0.13	0.21	973	999
Annual discount rate	0	10%	966	1,038

Appendix Table 3E: Univariate sensitivity analysis results, Swaziland (base-case ICER = US$770 / DALY).

Parameter description	Low parameter value	High parameter value	ICER w/ low parameter value	ICER w/ high parameter value
Transmission parameter for individuals with smear-positive TB in 1950	9.9	12.6	819	751
Annual percentage decline in transmission parameter	0.003	0.008	742	827
Infectivity of smear-negative TB, relative to smear-positive TB	0.18	0.32	880	695
Fitness cost for drug-resistant TB strains (% of base-case value)	77%	130%	837	729
Rate of attending TB testing site, for individuals with active TB, DOTS, 1990 (% of base-case value)	56%	182%	783	762
Rate of attending TB testing site, for individuals with active TB, DOTS, 2010 (% of base-case value)	73%	193%	648	940
Rate of attending TB testing site, for individuals with active TB, non-DOTS, 1990 (% of base-case value)	48%	143%	751	789
Rate of attending TB testing site, for individuals with active TB, non-DOTS, 2010 (% of base-case value)	53%	153%	746	796
Rate ratio of TB testing, for individuals without active TB compared to those with active TB	0.011	0.019	731	810
Specificity of sputum smear microscopy	0.97	0.98	764	777
Specificity of sputum culture	0.98	0.99	770	771
Sensitivity of Xpert for TB, smear-negative TB	0.70	0.77	784	758
Sensitivity of Xpert for TB, smear-positive TB	0.98	0.99	774	767
Specificity of Xpert for TB	0.99	1.00	776	765
Sensitivity of Xpert for RIF resistance	0.96	0.99	767	774
Specificity of Xpert for RIF resistance	0.98	0.99	772	769
Probability of sputum culture following a negative sputum smear (status quo algorithm), treatment-naïve patients	0.15	0.27	713	837
Probability of sputum culture following a negative sputum smear (status quo algorithm), treatment-experienced patients	0.75	0.86	771	770
Probability of DST following a positive TB diagnosis (status quo algorithm), treatment-experienced patients	0.75	0.85	785	756
Probability of loss to follow-up between initial presentation and treatment initiation, with prompt diagnosis	0.12	0.19	767	775
Probability of loss to follow-up between initial presentation and treatment initiation, with delayed diagnosis	0.18	0.31	797	746
Treatment default rate, DOTS (% of base-case value)	75%	125%	766	772
Treatment default rate, non-DOTS	0.43	0.72	807	736
Probability of treatment success, for individuals with pan-sensitive TB completing first-line regimen (% of base-case value)	73%	121%	784	765
Risk ratio of treatment success, first-line regimen, semi-sensitive strain	0.79	0.87	774	767
Risk ratio of treatment success, first-line regimen, non-sensitive strain	0.35	0.58	782	763
Risk ratio of treatment success, second-line regimen, sensitive strain	0.91	0.95	776	765
Risk ratio of treatment success, second-line regimen, non-sensitive strain	0.33	0.57	791	758
Risk ratio of treatment success, non-DOTS regimen, non-MDR strain	0.65	0.81	769	772

Parameter description	Low parameter value	High parameter value	ICER w/ low parameter value	ICER w/ high parameter value
Risk ratio of treatment success, non-DOTS regimen, MDR strain	0.33	0.55	778	764
Excess mortality rate for active TB, smear-negative	0.20	0.23	765	776
Excess mortality rate for active TB, smear-positive	0.27	0.39	800	748
Excess mortality rate for HIV, CD4 >350 cells/μl, no ART	0.006	0.010	768	773
Excess mortality rate for HIV, CD4 200-350 cells/μl, no ART	0.023	0.038	769	772
Excess mortality rate for HIV, CD4 <200 cells/μl, no ART	0.16	0.26	772	769
Excess mortality rate for HIV, on ART initiated at CD4 >350 cells/μl	0.006	0.010	770	770
Excess mortality rate for HIV, on ART initiated at CD4 200-350 cells/μl	0.017	0.029	770	770
Excess mortality rate for HIV, on ART initiated at CD4 <200 cells/μl	0.039	0.062	769	773
Excess mortality rate for advanced HIV (CD4 <200 cells/μl) and active TB without ART	0.65	1.08	759	779
TB treatment mortality rates (% of base-case values)	77%	130%	784	758
Partial immunity afforded by prior infection, HIV-negative	0.60	0.81	766	778
Partial immunity afforded by prior infection, HIV-positive, CD4 >350 cells/μl, no ART	0.33	0.56	769	772
Partial immunity afforded by prior infection, HIV-positive, CD4 200-350 cells/μl, no ART	0.18	0.31	769	772
Partial immunity afforded by prior infection, HIV-positive, CD4 <200 cells/μl, no ART	0.18	0.33	768	773
Probability of fast breakdown to active TB, with new infection, HIV-negative	0.10	0.12	795	751
Probability of fast breakdown to active TB, with new infection, HIV-positive, CD4 >350 cells/μl, no ART	0.26	0.44	789	756
Probability of fast breakdown to active TB, with new infection, HIV-positive, CD4 200-350 cells/μl, no ART	0.58	0.76	771	770
Probability of fast breakdown to active TB, with new infection, HIV-positive, CD4 <200 cells/μl, no ART	0.88	1.00	772	770
Probability of smear-positivity, for incident TB cases, HIV-negative	0.54	0.72	715	842
Probability of smear-positivity, for incident TB cases, HIV-positive, CD4 >350 cells/μl, no ART	0.36	0.60	749	795
Probability of smear-positivity, for incident TB cases, HIV-positive, CD4 200-350 cells/μl, no ART	0.27	0.45	764	778
Probability of smear-positivity, for incident TB cases, HIV-positive, CD4 <200 cells/μl, no ART	0.27	0.45	763	779
Rate of breakdown from latent/recovered to active TB, HIV-negative	0.0005	0.00128	779	763
Rate of breakdown from latent/recovered to active TB, HIV-positive, CD4 >350 cells/μl, no ART	0.002	0.004	773	768
Rate of breakdown from latent/recovered to active TB, HIV-positive, CD4 200-350 cells/μl, no ART	0.08	0.12	773	769
Rate of breakdown from latent/recovered to active TB, HIV-positive, CD4 <200 cells/μl, no ART	0.14	0.24	777	769
Rate of conversion from smear-negative to smear-positive active TB	0.012	0.018	769	772
Rate of self-cure for active TB, HIV-negative	0.18	0.24	756	785
Rate of self-cure for active TB, HIV-positive, CD4 >350 cells/μl, no ART	0.07	0.13	765	775

Parameter description	Low parameter value	High parameter value	ICER w/ low parameter value	ICER w/ high parameter value
Probability that failed treatment cases are correctly identified and returned to treatment	0.38	0.65	774	767
Rates of acquisition of TB drug resistance (% of base-case value)	70%	116%	716	833
HIV incidence trend, post-2011 (% of base-case value)	97%	102%	772	769
Rate of HIV progression for individuals not on ART, from CD4 >350 cells/μl to CD4 200-350 cells/μl	0.11	0.17	751	785
Rate of HIV progression for individuals not on ART, from CD4 200-350 cells/μl to CD4 <200 cells/μl	0.39	0.66	760	778
Future ART coverage for treatment-eligible HIV-positive individuals	0.66	0.92	722	814
Effectiveness of ART in reversing effect of HIV on TB natural history	0.56	0.78	745	802
Per-test cost of smear diagnosis	3.4	5.0	780	761
Per-test cost of culture	10.0	11.9	773	767
Per-test cost of chest X-ray	9.4	13.5	770	771
Per-test cost of drug sensitivity testing	48.6	64.8	773	768
Cost of outpatient diagnostic visit	4.6	8.0	779	762
Cost of outpatient treatment visit	3.1	5.4	757	784
Cost of inpatient care, per day	15.8	27.5	753	788
Monthly TB regimen costs (% of base-case value)	0.72	1.20	755	786
Monthly frequency of treatment activities, averaged over treatment course, clinic visits (first-line)	4.4	7.4	766	775
Monthly frequency of treatment activities, averaged over treatment course, clinic visits (second-line)	16.5	27.4	761	780
Monthly frequency of treatment activities, averaged over treatment course, monitoring smears (first-line)	0.76	1.29	770	771
Monthly frequency of treatment activities, averaged over treatment course, monitoring smears (second-line)	0.71	1.24	770	771
Monthly frequency of treatment activities, averaged over treatment course, sputum cultures (second-line)	0.34	0.57	770	771
Monthly frequency of treatment activities, averaged over treatment course, chest X-rays (second-line)	0.10	0.18	770	771
Number of months of inpatient care with MDR-TB treatment	2.9	5.2	753	788
Monthly cost of ART	73.7	89.6	733	808
Disability weight, active TB	0.21	0.35	814	731
Disability weight, HIV-positive, CD4 >350 cells/μl, no ART	0.10	0.17	767	774
Disability weight, HIV-positive, CD4 200-350 cells/μl, no ART	0.25	0.41	770	770
Disability weight, HIV-positive, CD4 <200 cells/μl, no ART	0.36	0.62	766	775
Disability weight, HIV-positive, on ART initiated at CD4 >350 cells/μl	0.11	0.17	767	774
Disability weight, HIV-positive, on ART initiated at CD4 200-350 cells/μl	0.12	0.19	767	774
Disability weight, HIV-positive, on ART initiated at CD4 <200 cells/μl	0.13	0.21	759	783
Annual discount rate	0	10%	758	802

5.3 Alternative scenarios relating to HIV treatment, TB diagnostic algorithms and MDR-TB treatment

In addition to the one-way sensitivity analyses described above, we defined a range of additional scenarios that included alternative assumptions regarding HIV treatment, TB diagnostic algorithms, and MDR-TB treatment components. In each of these further analyses, we adjusted the model inputs relating to each new scenario then re-ran the whole simulation, calculating point estimates and posterior 95% intervals as described for the main analysis.

The cost-effectiveness ratios from the main analysis aim to capture the major changes in health system resource use and health outcomes resulting from the adoption of the Xpert algorithm, including increases in TB treatment and HIV treatment volume. The increase in TB treatment volume is a direct consequence of better case-finding under the Xpert algorithm. The increase in ART volume is an indirect consequence of Xpert introduction, resulting from improved survival of TB-HIV coinfected individuals currently receiving ART or those who would go on to receive ART in the future. In order to disentangle the direct effect of Xpert from this secondary effect through HIV survival, we constructed a scenario in which access to ART under a scaled-up Xpert approach was constrained to be the same as in the status quo scenario (as might be the case if the future HIV treatment budget were fixed and did not increase as a function of HIV treatment need). While artificial, this scenario allowed us to estimate the cost-effectiveness of Xpert adoption separate from the effects on HIV treatment. In this scenario, incremental costs and DALYs averted dropped by 35-40% and 10-15%, respectively, compared to the main analysis, and the cost per DALY averted dropped to US$656 [386 - 1,115] over a 10-year analytic horizon (assuming a US$30 per-test cost for Xpert). While this analysis is informative, we emphasize that a policy-maker aiming to maximize the effectiveness of the entire health portfolio should use the ICER generated in the main analysis unless planning to limit ART enrollment without consideration of actual treatment need.

We also investigated the potential consequences of time-trends in ART prices. In the main analysis the per-patient costs of ART were assumed to be constant. Recent analyses have observed a net downward trend [86], although an upward trend might be possible, with the uncertainty reflecting a tradeoff between price reductions and increasing use of more expensive second-line therapies. We investigated the possible consequences of ART price reductions by recalculating the results under an assumption that ART costs would drop by 50% every 10 years. This change reduced the cost per DALY to US$812 [522-1,283] over the 10-year analytic horizon and to US$552 [320 - 1,023] over 20 years, reductions of 15% and 30% compared to the results in the main analysis.

Similar to ART, MDR-TB treatment is another expensive service with increased volume under the Xpert strategy, due to both better TB case-finding and better identification of drug resistance. Inpatient care adds substantially to MDR-TB treatment costs, yet there is limited evidence that it improves treatment outcomes [82,96]. We constructed a scenario to investigate how Xpert cost-effectiveness would change if inpatient care were no longer required for MDR-TB treatment, assuming this would produce no net change in health outcomes. This change was found to reduce incremental health system costs of the Xpert algorithm by 15%, and to reduce the cost per DALY averted by the same percentage, to US$812 [522 - 1,283] over a 10-year analytic horizon.

Our main analysis focused on an Xpert algorithm in which a negative Xpert diagnosis would be treated as definitive, whereas South Africa has developed local guidelines that call for more aggressive investigation (including culture, chest X-ray and antibiotic trial) for Xpert-negative individuals who have positive or unknown HIV status [97]. We compared this algorithm to the Xpert algorithm used in the main analysis, assuming that all truly HIV-positive individuals would be categorized as 'HIV positive or unknown' at TB diagnosis, while 50% (range 25-75%) of all truly HIV-negative individuals would have a prior HIV test confirming this status, based on recent population based surveys [66-68]. In this comparison the South African Xpert algorithm was found to increase incremental costs by 60% and incremental DALYs averted by 27%, which resulted in an incremental cost-effectiveness ratio of US$2,128 [1,215-3,954] per DALY averted (10-year analytic horizon, US$30 Xpert cost) for the more aggressive strategy compared to the base-case Xpert algorithm. We also conducted sensitivity analyses on how the cost-effectiveness of Xpert might change if all individuals with a positive Xpert RIF result receive empiric MDR-TB treatment while waiting for the DST result to be returned (a delay estimated at 80 days [98]). This change had a modest effect, raising incremental costs by 8%, and resulting in a cost per DALY averted of US$1,038 [683-1,584] (10-year analytic horizon, US$30 year Xpert cost).

In another set of sensitivity analyses we tested the robustness of the results to changes in the status quo algorithm. In the main analysis we assumed incomplete access to TB culture and DST. If instead we assumed 100% access to TB culture, such that all treatment-experienced patients testing negative with sputum smear received a confirmatory TB culture, incremental costs and DALYs averted by the Xpert algorithm both dropped by 3%, with little change in the cost per DALY averted, which was estimated as US$956 [628-1,491]. If we also assumed that 100% of treatment-experienced patients diagnosed with TB received DST, then incremental costs and DALYS averted by the Xpert algorithm dropped by 15% and 4%, respectively, compared to the main analysis, and the cost per DALY averted dropped marginally to US$851 [570-1,323]. We also conducted a three-way sensitivity analysis that considered a much wider range of estimates for culture and DST access, investigating the possibility of country-level differences in access to these diagnostic services. The results of these changes on incremental costs, incremental health benefits, and incremental cost-effectiveness ratios are shown in Figure S4. This figure shows that if use of culture under the status quo algorithm is higher than the value used in the main analysis, this would reduce the incremental costs and health benefits produced by adopting Xpert and result in a less favorable cost-effectiveness ratio. In some countries very high values of culture use would result in the status quo strategy dominating the Xpert strategy, i.e. having lower costs and greater health benefits. The coverage levels that produce such a result (80% of all treatment- naïve and treatment-experienced patients diagnosed via culture), however, are unlikely to be in place at present given current infrastructure and program constraints. Higher than expected DST access under the status quo would produce modest reductions in incremental costs and minimal changes in cost-effectiveness ratios.

Similarly, allowing for the possibility of clinical diagnosis as part of the base case algorithm did not substantially alter the cost-effectiveness of Xpert. When we compared the Xpert algorithm to an altered status quo algorithm in which all individuals with suspected TB testing negative with sputum smear receive clinical diagnosis (which might include chest X-ray or antibiotic trial [49]) to

confirm the negative diagnosis, this increased the incremental cost-effectiveness ratio for Xpert by approximately 10%, to US$1,052 [643 - 1,785], with both incremental costs and DALYs averted approximately one-third lower than estimated in the main analysis.

5.4 Cost-effectiveness acceptability curves

As described in the main paper, cost-effectiveness acceptibility curves [99] were constructed for 10-year and 30-year analytic horizons, showing how the probability that the Xpert strategy is optimal (i.e. cost-effective) changes as a function of the willingness to pay for health benefits (see Figure S5). If society were willing to pay up to the average per capita GDP (US$6,850 for the region) for each averted DALY, our results suggest essentially no uncertainty in the conclusion that Xpert would be cost-effective. At a threshold of only US$1,000 (representing <15% of per capita GDP in the region), the probability that Xpert would be regarded as cost-effective was 85% when we considered the benefits that would accumulate over 20 years, or 55% over a 10-year horizon.

6 References

1. Baltussen R, Floyd K, Dye C (2005) Cost effectiveness analysis of strategies for tuberculosis control in developing countries. BMJ 331: 1364.

2. Cohen T, Lipsitch M, Walensky RP, Murray M (2006) Beneficial and perverse effects of isoniazid preventive therapy for latent tuberculosis infection in HIV-tuberculosis coinfected populations. Proc Natl Acad Sci U S A 103: 7042-7047.

3. Dowdy DW, Chaisson RE (2009) The persistence of tuberculosis in the age of DOTS: reassessing the effect of case detection. Bull World Health Organ 87: 296-304.

4. Dye C, Garnett GP, Sleeman K, Williams BG (1998) Prospects for worldwide tuberculosis control under the WHO DOTS strategy. Directly observed short-course therapy. Lancet 352: 1886-1891.

5. Dye C, Williams BG (2000) Criteria for the control of drug-resistant tuberculosis. Proc Natl Acad Sci U S A 97: 8180-8185.

6. Murray CJ, Salomon JA (1998) Modeling the impact of global tuberculosis control strategies. Proc Natl Acad Sci U S A 95: 13881-13886.

7. Salomon JA, Lloyd-Smith JO, Getz WM, Resch S, Sanchez MS, et al. (2006) Prospects for advancing tuberculosis control efforts through novel therapies. PLoS Med 3: e273.

8. Daley CL, Small PM, Schecter GF, Schoolnik GK, McAdam RA, et al. (1992) An outbreak of tuberculosis with accelerated progression among persons infected with the human immunodeficiency virus. An analysis using restriction-fragment-length polymorphisms. N Engl J Med 326: 231-235.

9. Shafer RW, Singh SP, Larkin C, Small PM (1995) Exogenous reinfection with multidrug-resistant Mycobacterium tuberculosis in an immunocompetent patient. Tuber Lung Dis 76: 575-577.

10. Bucher HC, Griffith LE, Guyatt GH, Sudre P, Naef M, et al. (1999) Isoniazid prophylaxis for tuberculosis in HIV infection: a meta-analysis of randomized controlled trials. AIDS 13: 501-507.

11. Corbett EL, Watt CJ, Walker N, Maher D, Williams BG, et al. (2003) The growing burden of tuberculosis: global trends and interactions with the HIV epidemic. Arch Intern Med 163: 1009-1021.

12. Elliott AM, Halwiindi B, Hayes RJ, Luo N, Tembo G, et al. (1993) The impact of human immunodeficiency virus on presentation and diagnosis of tuberculosis in a cohort study in Zambia. J Trop Med Hyg 96: 1-11.

13. Getahun H, Harrington M, O'Brien R, Nunn P (2007) Diagnosis of smear-negative pulmonary tuberculosis in people with HIV infection or AIDS in resource-constrained settings: informing urgent policy changes. Lancet 369: 2042-2049.

14. Manosuthi W, Tantanathip P, Chimsuntorn S, Eampokarap B, Thongyen S, et al. (2010) Treatment outcomes of patients co-infected with HIV and tuberculosis who received a nevirapine-based antiretroviral regimen: a four-year prospective study. Int J Infect Dis 14: e1013-1017.

15. van der Sande MA, Schim van der Loeff MF, Bennett RC, Dowling M, Aveika AA, et al. (2004) Incidence of tuberculosis and survival after its diagnosis in patients infected with HIV-1 and HIV-2. AIDS 18: 1933-1941.

16. Adam MA, Johnson LF (2009) Estimation of adult antiretroviral treatment coverage in South Africa. S Afr Med J 99: 661-667.

17. Granich RM, Gilks CF, Dye C, De Cock KM, Williams BG (2009) Universal voluntary HIV testing with immediate antiretroviral therapy as a strategy for elimination of HIV transmission: a mathematical model. Lancet 373: 48-57.

18. Stover J, Bollinger L, Avila C (2011) Estimating the Impact and Cost of the WHO 2010 Recommendations for Antiretroviral Therapy. AIDS Res Treat 2011: 738271.

19. UNAIDS Reference Group on Estimates Modelling and Projections (2011) Technical Meeting to Review Spectrum 2011 and considering potential bias in DHS and ANC data. Geneva: Joint United Nations Programme on HIV/AIDS.

20. Lipsitch M, Colijn C, Cohen T, Hanage WP, Fraser C (2009) No coexistence for free: neutral null models for multistrain pathogens. Epidemics 1: 2-13.

21. United Nations Population Division (2011) World Population Prospects: The 2010 Revision, CD-ROM Edition. Geneva: United Nations, Department of Economic and Social Affairs, Population Division.

22. Cohen T, Murray M (2004) Modeling epidemics of multidrug-resistant M. tuberculosis of heterogeneous fitness. Nat Med 10: 1117-1121.

23. Dowdy DW, Chaisson RE, Maartens G, Corbett EL, Dorman SE (2008) Impact of enhanced tuberculosis diagnosis in South Africa: a mathematical model of expanded culture and drug susceptibility testing. Proc Natl Acad Sci U S A 105: 11293-11298.

24. Dowdy DW, O'Brien MA, Bishai D (2008) Cost-effectiveness of novel diagnostic tools for the diagnosis of tuberculosis. Int J Tuberc Lung Dis 12: 1021-1029.

25. WHO (2011) Rapid Implementation of the Xpert MTB/RIF diagnostic test: Technical and operational 'How-to'. Geneva: WHO.

26. Boehme CC, Nabeta P, Hillemann D, Nicol MP, Shenai S, et al. (2010) Rapid molecular detection of tuberculosis and rifampin resistance. N Engl J Med 363: 1005-1015.

27. Frieden T (2004) Toman's tuberculosis: case detection, treatment and monitoring: questions and answers (2nd edition). Geneva: WHO.

28. Levy H, Feldman C, Sacho H, van der Meulen H, Kallenbach J, et al. (1989) A reevaluation of sputum microscopy and culture in the diagnosis of pulmonary tuberculosis. Chest 95: 1193-1197.

29. WHO (2011) Global TB Database (http://www.who.int/tb/country/data/download/en/index.html, accessed September 30 2011). Geneva: WHO.

30. WHO (2009) TB Impact Measurement: Policy and recommendations for how to assess the epidemiological burden of TB and the impact of TB control. Stop TB policy paper; no 2. Geneva: WHO.

31. Lew W, Pai M, Oxlade O, Martin D, Menzies D (2008) Initial drug resistance and tuberculosis treatment outcomes: systematic review and meta-analysis. Ann Intern Med 149: 123-134.

32. UNAIDS (2010) Towards universal access: Scaling up priority HIV/AIDS interventions in the health sector. Progress report 2010. Geneva: UNAIDS.

33. WHO (2006) Antiretroviral Therapy for HIV Infection in Adults and Adolescents: Recommendations for a public health approach. 2006 Revision. Geneva: WHO.

34. WHO (2010) Antiretroviral therapy for HIV infection in adults and adolescents: recommendations for a public health approach – 2010 Revision. Geneva: WHO.

35. Anglaret X, Minga A, Gabillard D, Ouassa T, Messou E, et al. (2012) AIDS and non-AIDS morbidity and mortality across the spectrum of CD4 cell counts in HIV-infected adults before starting antiretroviral therapy in Cote d'Ivoire. Clin Infect Dis 54: 714-723.

36. Badri M, Lawn SD, Wood R (2006) Short-term risk of AIDS or death in people infected with HIV-1 before antiretroviral therapy in South Africa: a longitudinal study. Lancet 368: 1254-1259.

37. Egger M, May M, Chene G, Phillips AN, Ledergerber B, et al. (2002) Prognosis of HIV-1-infected patients starting highly active antiretroviral therapy: a collaborative analysis of prospective studies. Lancet 360: 119-129.

38. May M, Sterne JA, Sabin C, Costagliola D, Justice AC, et al. (2007) Prognosis of HIV-1-infected patients up to 5 years after initiation of HAART: collaborative analysis of prospective studies. AIDS 21: 1185-1197.

39. Phillips A, Pezzotti P, Cascade Collaboration (2004) Short-term risk of AIDS according to current CD4 cell count and viral load in antiretroviral drug-naive individuals and those treated in the monotherapy era. AIDS 18: 51-58.

40. When To Start Consortium, Sterne JA, May M, Costagliola D, de Wolf F, et al. (2009) Timing of initiation of antiretroviral therapy in AIDS-free HIV-1-infected patients: a collaborative analysis of 18 HIV cohort studies. Lancet 373: 1352-1363.

41. WHO (2011) Global Price Reporting Mechanism (http://www.who.int/hiv/amds/gprm/en/, accessed May 21 2011). Geneva: WHO.

42. Meyer-Rath G, Bistline K, Long L, MacLeod B, Sanne I, et al. (2011) The incremental cost of introducing Xpert® MTB/RIF into the South African national tuberculosis programme: Results of the National TB Cost Model 2011/12 - 2016/17. Johannesburg: University of Witwatersrand, Health Economics and Epidemiology Research Office.

43. Murray CJL, Lopez AD (1996) The Global Burden of Disease: A comprehensive assessment of mortality and disability from diseases, injuries and risk factors in 1990 and projected to 2020. Cambridge, MA: Harvard University Press.

44. WHO (2004) Global Burden of Disease 2004 Update: Disability Weights for Diseases and Conditions. Geneva: WHO.

45. Flanagan W, McIntosh CN, Le Petit C, Berthelot JM (2006) Deriving utility scores for co-morbid conditions: a test of the multiplicative model for combining individual condition scores. Popul Health Metr 4: 13.

46. Mathers CD, R. S, Salomon JA, L. MCJ, Lopez AD (2000) Estimates of DALE for 191 countries: methods and results. Geneva: WHO.

47. Dye C, Espinal MA (2001) Will tuberculosis become resistant to all antibiotics? Proc Biol Sci 268: 45-52.

48. Luciani F, Sisson SA, Jiang H, Francis AR, Tanaka MM (2009) The epidemiological fitness cost of drug resistance in Mycobacterium tuberculosis. Proc Natl Acad Sci U S A 106: 14711-14715.

49. Vassall A, van Kampen S, Sohn H, Michael JS, John KR, et al. (2011) Rapid Diagnosis of Tuberculosis with the Xpert MTB/RIF Assay in High Burden Countries: A Cost-Effectiveness Analysis. PLoS Med 8: e1001120.

50. Espinal MA, Kim SJ, Suarez PG, Kam KM, Khomenko AG, et al. (2000) Standard short-course chemotherapy for drug-resistant tuberculosis: treatment outcomes in 6 countries. JAMA 283: 2537-2545.

51. Menzies D, Benedetti A, Paydar A, Royce S, Madhukar P, et al. (2009) Standardized treatment of active tuberculosis in patients with previous treatment and/or with mono-resistance to isoniazid: a systematic review and meta-analysis. PLoS Med 6: e1000150.

52. Migliori GB, Espinal M, Danilova ID, Punga VV, Grzemska M, et al. (2002) Frequency of recurrence among MDR-tB cases 'successfully' treated with standardised short-course chemotherapy. Int J Tuberc Lung Dis 6: 858-864.

53. Orenstein EW, Basu S, Shah NS, Andrews JR, Friedland GH, et al. (2009) Treatment outcomes among patients with multidrug-resistant tuberculosis: systematic review and meta-analysis. Lancet Infect Dis 9: 153-161.

54. Verma R, Khanna P, Prinja S (2010) A Comparative Study Between Dots & Non-Dots Patients In Two Districts Of Haryana, India. Internet Journal of Epidemiology 8: 246e.

55. Vynnycky E, Fine PE (1997) The natural history of tuberculosis: the implications of age-dependent risks of disease and the role of reinfection. Epidemiol Infect 119: 183-201.

56. Ferebee SH (1970) Controlled chemoprophylaxis trials in tuberculosis. A general review. Bibl Tuberc 26: 28-106.

57. Datiko DG, Lindtjorn B (2010) Cost and cost-effectiveness of smear-positive tuberculosis treatment by Health Extension Workers in Southern Ethiopia: a community randomized trial. PLoS One 5: e9158.

58. WHO (2008) Guidelines for the programmatic management of drug-resistant tuberculosis: Emergency update 2008. Geneva: WHO.

59. Mellors JW, Margolick JB, Phair JP, Rinaldo CR, Detels R, et al. (2007) Prognostic value of HIV-1 RNA, CD4 cell count, and CD4 Cell count slope for progression to AIDS and death in untreated HIV-1 infection. JAMA 297: 2349-2350.

60. Rodriguez B, Sethi AK, Cheruvu VK, Mackay W, Bosch RJ, et al. (2006) Predictive value of plasma HIV RNA level on rate of CD4 T-cell decline in untreated HIV infection. JAMA 296: 1498-1506.

61. Williams BG, Korenromp EL, Gouws E, Schmid GP, Auvert B, et al. (2006) HIV infection, antiretroviral therapy, and CD4+ cell count distributions in African populations. J Infect Dis 194: 1450-1458.

62. Wolbers M, Babiker A, Sabin C, Young J, Dorrucci M, et al. (2010) Pretreatment CD4 cell slope and progression to AIDS or death in HIV-infected patients initiating antiretroviral therapy--the CASCADE collaboration: a collaboration of 23 cohort studies. PLoS Med 7: e1000239.

63. Badri M, Wilson D, Wood R (2002) Effect of highly active antiretroviral therapy on incidence of tuberculosis in South Africa: a cohort study. Lancet 359: 2059-2064.

64. Golub JE, Saraceni V, Cavalcante SC, Pacheco AG, Moulton LH, et al. (2007) The impact of antiretroviral therapy and isoniazid preventive therapy on tuberculosis incidence in HIV-infected patients in Rio de Janeiro, Brazil. AIDS 21: 1441-1448.

65. Lawn SD, Kranzer K, Wood R (2009) Antiretroviral Therapy for Control of the HIVassociated Tuberculosis Epidemic in Resource- Limited Settings. Clin Chest Med 30: 685-699.

66. Central Statistical Office Swaziland, Macro International Inc (2008) Swaziland Demographic and Health Survey 2006-07. Swaziland: Central Statistical Office and Macro International Inc.

67. Ministry of Health and Social Welfare Lesotho, ICF Macro (2010) Lesotho Demographic and Health Survey 2009. Lesotho: Ministry of Health and Social Welfare Lesotho & ICF Macro.

68. Peltzer K, Matseke G, Mzolo T, Majaja M (2009) Determinants of knowledge of HIV status in South Africa: results from a population-based HIV survey. BMC Public Health 9: 174.

69. Meyer-Rath G, Schnippel K, Long L, MacLeod W, Sanne I, et al. (2012) The impact and cost of scaling up GeneXpert MTB/RIF in South Africa. PLoS One 7: e36966.

70. Schnippel K, Meyer-Rath G, Long L, Macleod W, Sanne I, et al. (2012) Scaling up Xpert MTB/RIF technology: the costs of laboratory- vs. clinic-based roll-out in South Africa. Trop Med Int Health 17(9): 1142-1151.

71. Albert H (2004) Economic analysis of the diagnosis of smear-negative pulmonary tuberculosis in South Africa: incorporation of a new rapid test, FASTPlaqueTB, into the diagnostic algorithm. Int J Tuberc Lung Dis 8: 240-247.

72. Floyd K, Skeva J, Nyirenda T, Gausi F, Salaniponi F (2003) Cost and cost-effectiveness of increased community and primary care facility involvement in tuberculosis care in Lilongwe District, Malawi. Int J Tuberc Lung Dis 7: S29-37.

73. Hausler HP, Sinanovic E, Kumaranayake L, Naidoo P, Schoeman H, et al. (2006) Costs of measures to control tuberculosis/HIV in public primary care facilities in Cape Town, South Africa. Bull World Health Organ 84: 528-536.

74. Hudson CP, Wood R, Maartens G (2000) Diagnosing HIV-associated tuberculosis: reducing costs and diagnostic delay. Int J Tuberc Lung Dis 4: 240-245.

75. Nganda B, Wang'ombe J, Floyd K, Kangangi J (2003) Cost and cost-effectiveness of increased community and primary care facility involvement in tuberculosis care in Machakos District, Kenya. Int J Tuberc Lung Dis 7: S14-20.

76. Samandari T, Bishai D, Luteijn M, Mosimaneotsile B, Motsamai O, et al. (2011) Costs and consequences of additional chest x-ray in a tuberculosis prevention program in Botswana. Am J Respir Crit Care Med 183: 1103-1111.

77. Suarez PG, Floyd K, Portocarrero J, Alarcon E, Rapiti E, et al. (2002) Feasibility and cost-effectiveness of standardised second-line drug treatment for chronic tuberculosis patients: a national cohort study in Peru. Lancet 359: 1980-1989.

78. van Cleeff MR, Kivihya-Ndugga LE, Meme H, Odhiambo JA, Klatser PR (2005) The role and performance of chest X-ray for the diagnosis of tuberculosis: a cost-effectiveness analysis in Nairobi, Kenya. BMC Infect Dis 5: 111.

79. Acuna-Villaorduna C, Vassall A, Henostroza G, Seas C, Guerra H, et al. (2008) Cost-effectiveness analysis of introduction of rapid, alternative methods to identify multidrug-resistant tuberculosis in middle-income countries. Clin Infect Dis 47: 487-495.

80. Resch SC, Salomon JA, Murray M, Weinstein MC (2006) Cost-effectiveness of treating multidrug-resistant tuberculosis. PLoS Med 3: e241.

81. Adam T, Evans DB, Murray CJ (2003) Econometric estimation of country-specific hospital costs. Cost Eff Resour Alloc 1: 3.

82. Loveday M, Wallengren K, Voce A, Margot B, Reddy T, et al. (2012) Comparing early treatment outcomes of MDR-TB in decentralised and centralised settings in KwaZulu-Natal, South Africa. Int Journal of Tub Luncg Dis 16: 209-215.

83. Bikilla AD, Jerene D, Robberstad B, Lindtjorn B (2009) Cost estimates of HIV care and treatment with and without anti-retroviral therapy at Arba Minch Hospital in southern Ethiopia. Cost Eff Resour Alloc 7: 6.

84. Bratt JH, Torpey K, Kabaso M, Gondwe Y (2011) Costs of HIV/AIDS outpatient services delivered through Zambian public health facilities. Trop Med Int Health 16: 110-118.

85. Harling G, Wood R (2007) The evolving cost of HIV in South Africa: changes in health care cost with duration on antiretroviral therapy for public sector patients. J Acquir Immune Defic Syndr 45: 348-354.

86. Menzies NA, Berruti AA, Berzon R, Filler S, Ferris R, et al. (2011) The cost of providing comprehensive HIV treatment in PEPFAR-supported programs. AIDS 25: 1753-1760.

87. Rosen S, Long L, Sanne I (2008) The outcomes and outpatient costs of different models of antiretroviral treatment delivery in South Africa. Trop Med Int Health 13: 1005-1015.

88. Tan Torres T, Baltussen R, Adam T, Hutubessy R, Acharya A, et al. (2003) Making Choices in Health: WHO Guide to Cost Effectiveness Analysis. Geneva: WHO.

89. Weinstein MC, Siegel JE, Gold MR, Kamlet MS, Russell LB (1996) Recommendations of the Panel on Cost-effectiveness in Health and Medicine. JAMA 276: 1253-1258.

90. Alkema L, Raftery AE, Clark SJ (2007) Probabilistic Projections of HIV Prevalence Using Bayesian Melding. Ann Appl Stat 1: 229-248.

91. Poole D, Raftery AE (2000) Inference for deterministic simulation models: The Bayesian melding approach. J Am Stat Assoc 95: 452.

92. Rubin D (1988) Using the SIR algorithm to simulate posterior distributions. In: Bernardo JM, DeGroot MH, Lindley DV, Smith AFM, editors. Bayesian Statistics 3. Oxford: Oxford University Press. pp 395–402

93. WHO (2010) Multidrug and extensively drug-resistant TB (M/XDR-TB): 2010 global report on surveillance and response. Geneva: WHO.

94. Iman RL, Helton JC, Campbell JE (1981) An approach to sensitivity analysis of computer models: part I—introduction, input variable selection and preliminary variable assessment. J Qual Technol 13: 174-183.

95. Iman RL, Helton JC, Campbell JE (1981) An approach to sensitivity analysis of computer models: part II—ranking of input variables, response surface validation, distribution effect, and technique synopsis variable assessment. J Qual Technol 13: 232-240.

96. Heller T, Lessells RJ, Wallrauch CG, Barnighausen T, Cooke GS, et al. (2010) Community-based treatment for multidrug-resistant tuberculosis in rural KwaZulu-Natal, South Africa. Int J Tuberc Lung Dis 14: 420-426.

97. National Health Laboratory Service (2012) Xpert Testing Algorithm. South Africa: National Health Laboratory Service.

98. Jacobson KR, Theron D, Kendall EA, Franke MF, Barnard M, et al. (2012) Implementation of GenoType MTBDRplus Reduces Time to Multidrug-Resistant Tuberculosis Therapy Initiation in South Africa. Manuscript Under Review.

99. Fenwick E, O'Brien BJ, Briggs A (2004) Cost-effectiveness acceptability curves--facts, fallacies and frequently asked questions. Health Econ 13: 405-415.

7 Supplementary figures

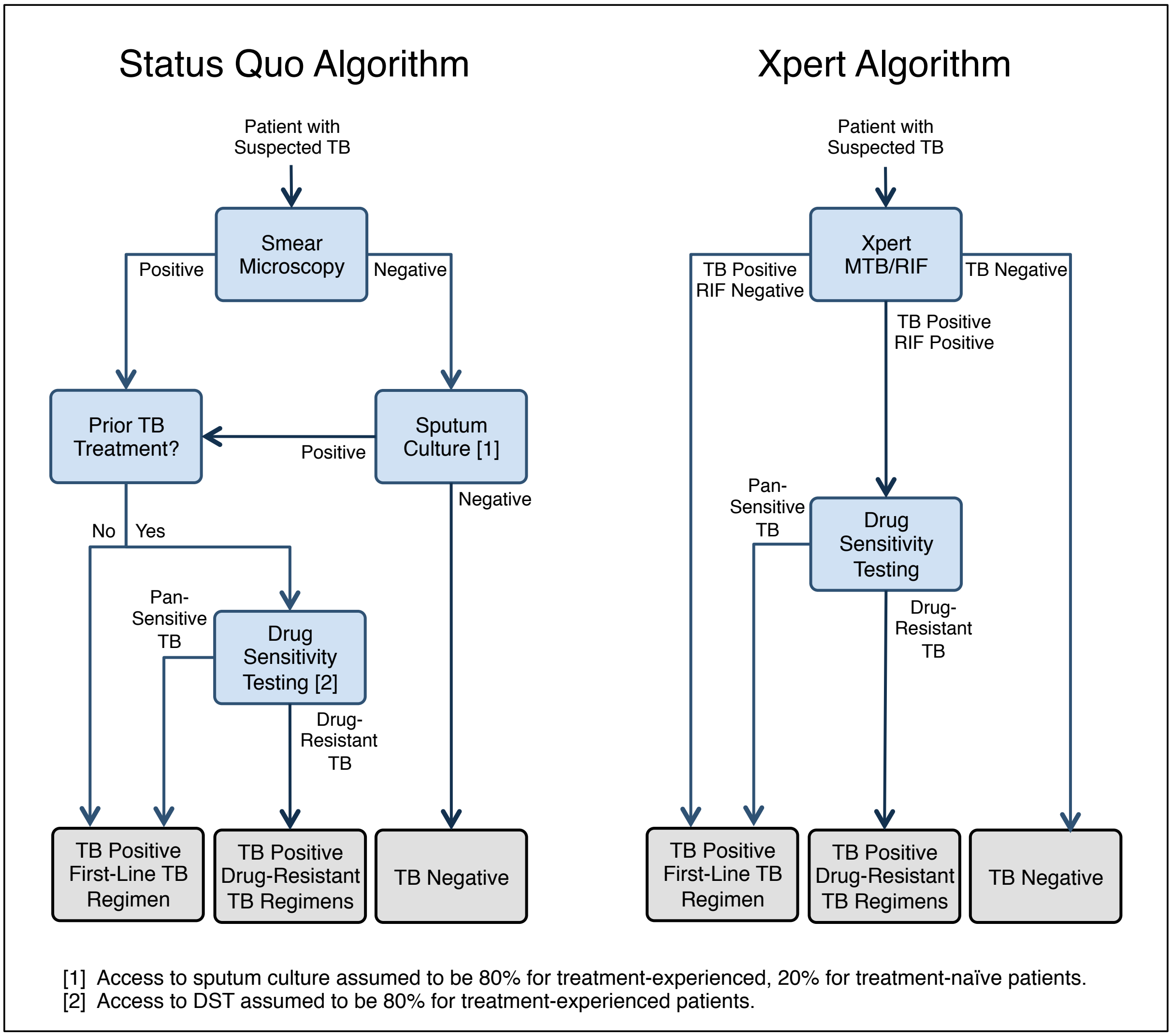

Figure S1: Status quo and Xpert diagnostic algorithms.

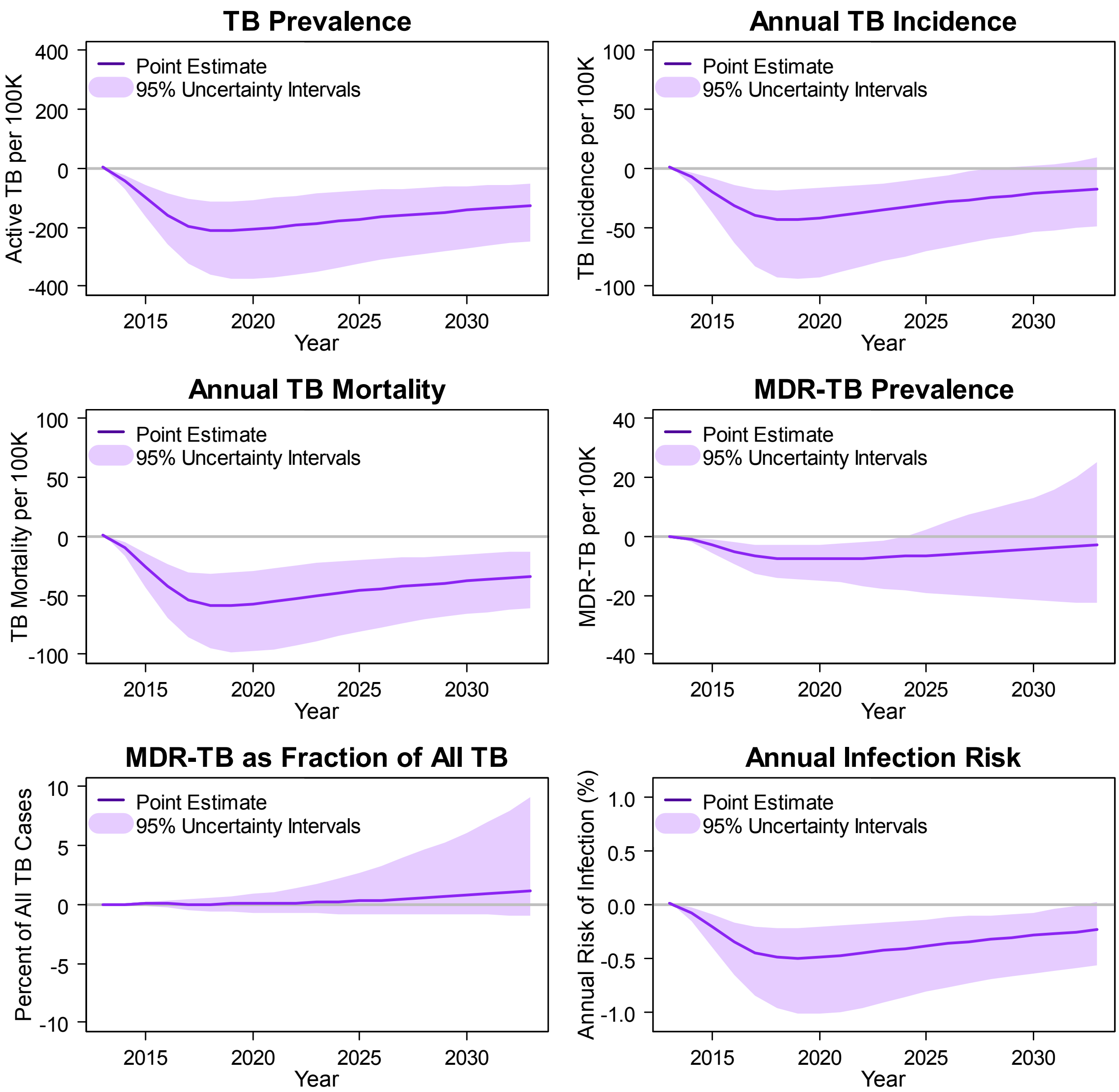

Figure S2: Incremental difference in epidemiologic outcomes between Xpert and status quo scenarios, 2012-2032.

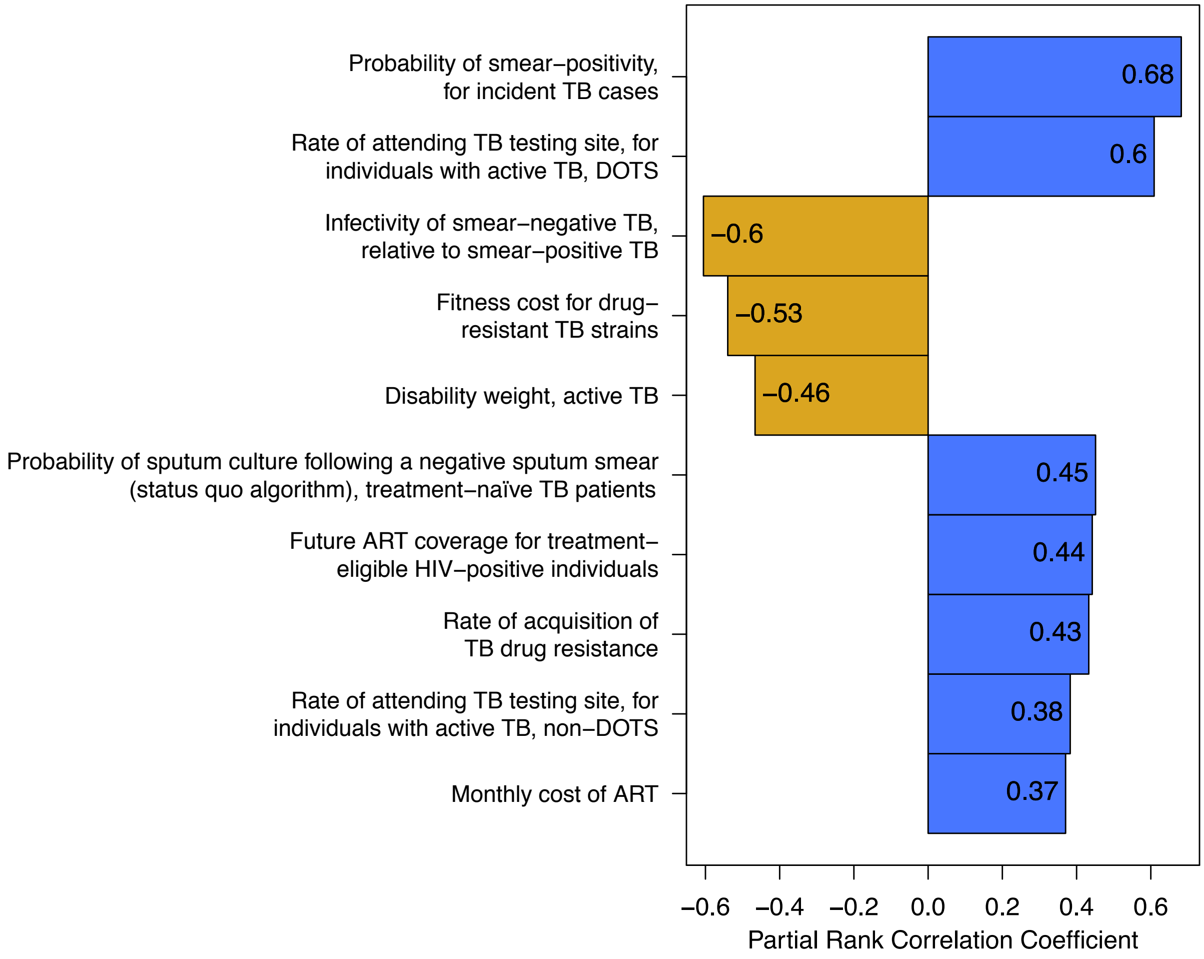

Figure S3: Partial rank correlation coefficients for 10 parameters with greatest influence on the cost-effectiveness of Xpert compared to status quo, South Africa, 10-year time horizon.

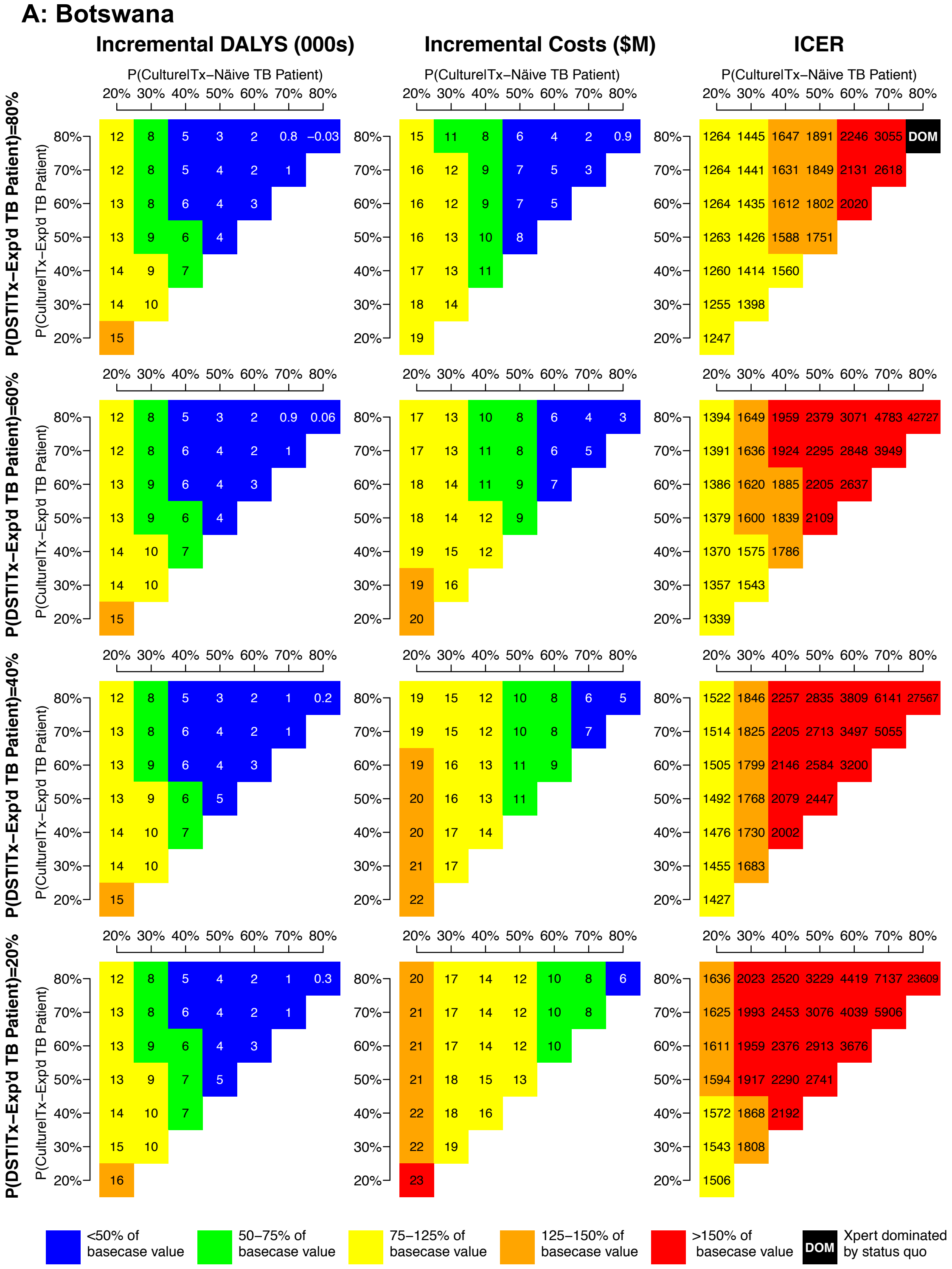

Figure S4: Three-way sensitivity analyses showing effects of changes in culture and DST coverage on major study outcomes, by country.

* Costs, DALYs and ICERs assessed over a 10-year analytic horizon with a US$30 Xpert unit cost. All other parameters held at their mean posterior values.

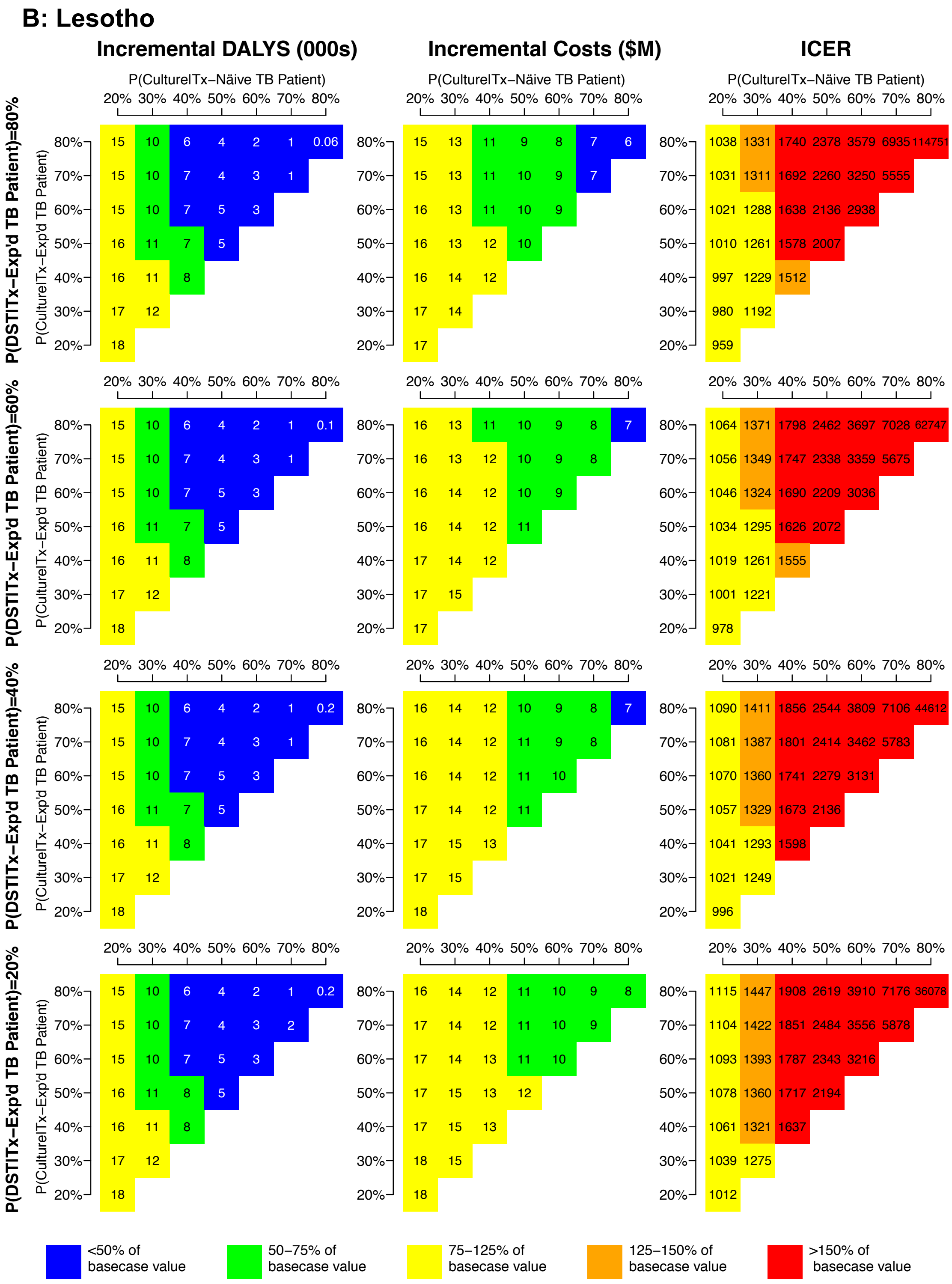

Figure S4 (continued): Three-way sensitivity analyses showing effects of changes in culture and DST coverage on major study outcomes, by country.

* Costs, DALYs and ICERs assessed over a 10-year analytic horizon with a US$30 Xpert unit cost. All other parameters held at their mean posterior values.

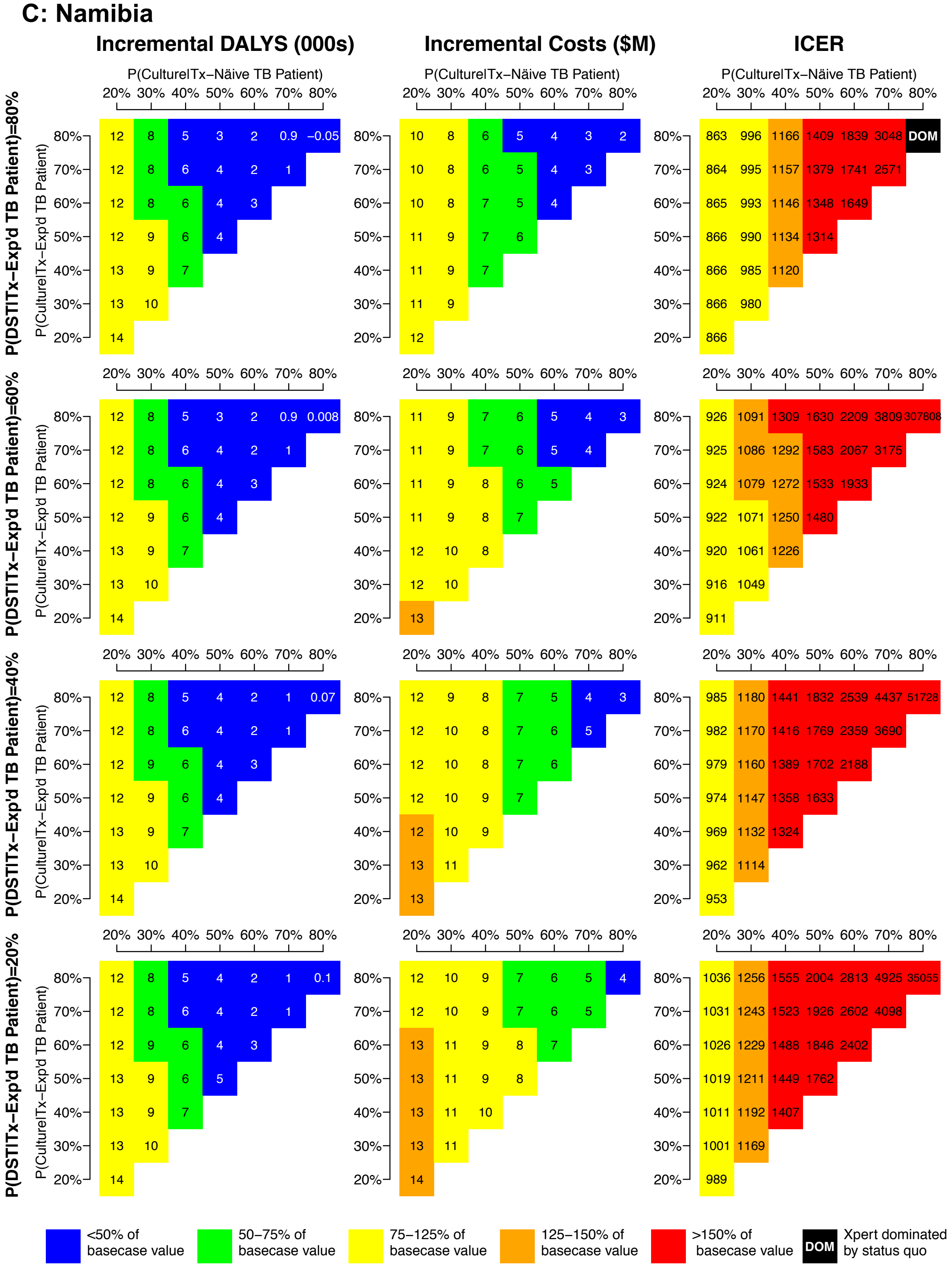

Figure S4 (continued): Three-way sensitivity analyses showing effects of changes in culture and DST coverage on major study outcomes, by country.

* Costs, DALYs and ICERs assessed over a 10-year analytic horizon with a US$30 Xpert unit cost. All other parameters held at their mean posterior values.

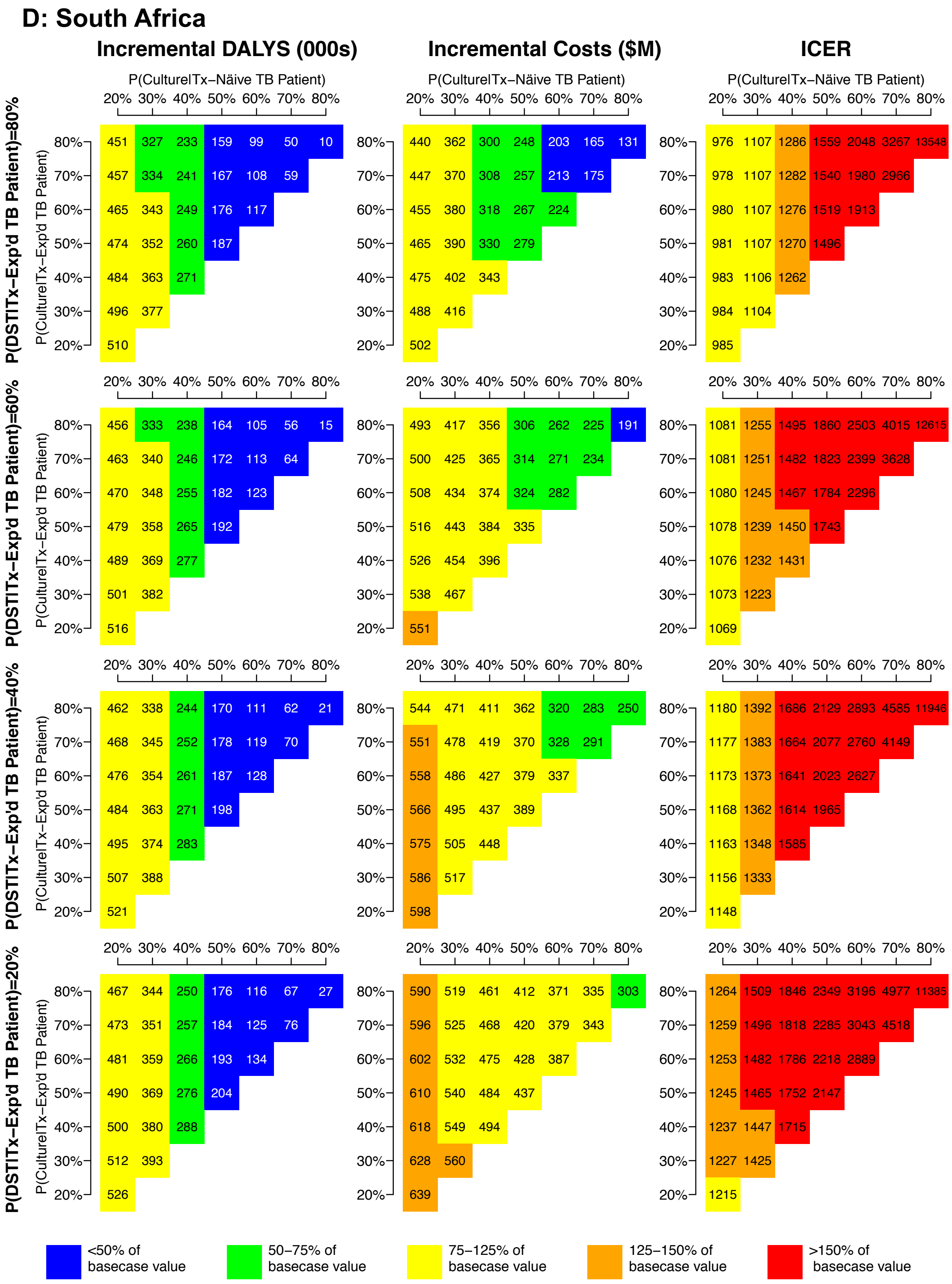

Figure S4 (continued): Three-way sensitivity analyses showing effects of changes in culture and DST coverage on major study outcomes, by country.

* Costs, DALYs and ICERs assessed over a 10-year analytic horizon with a US$30 Xpert unit cost. All other parameters held at their mean posterior values.

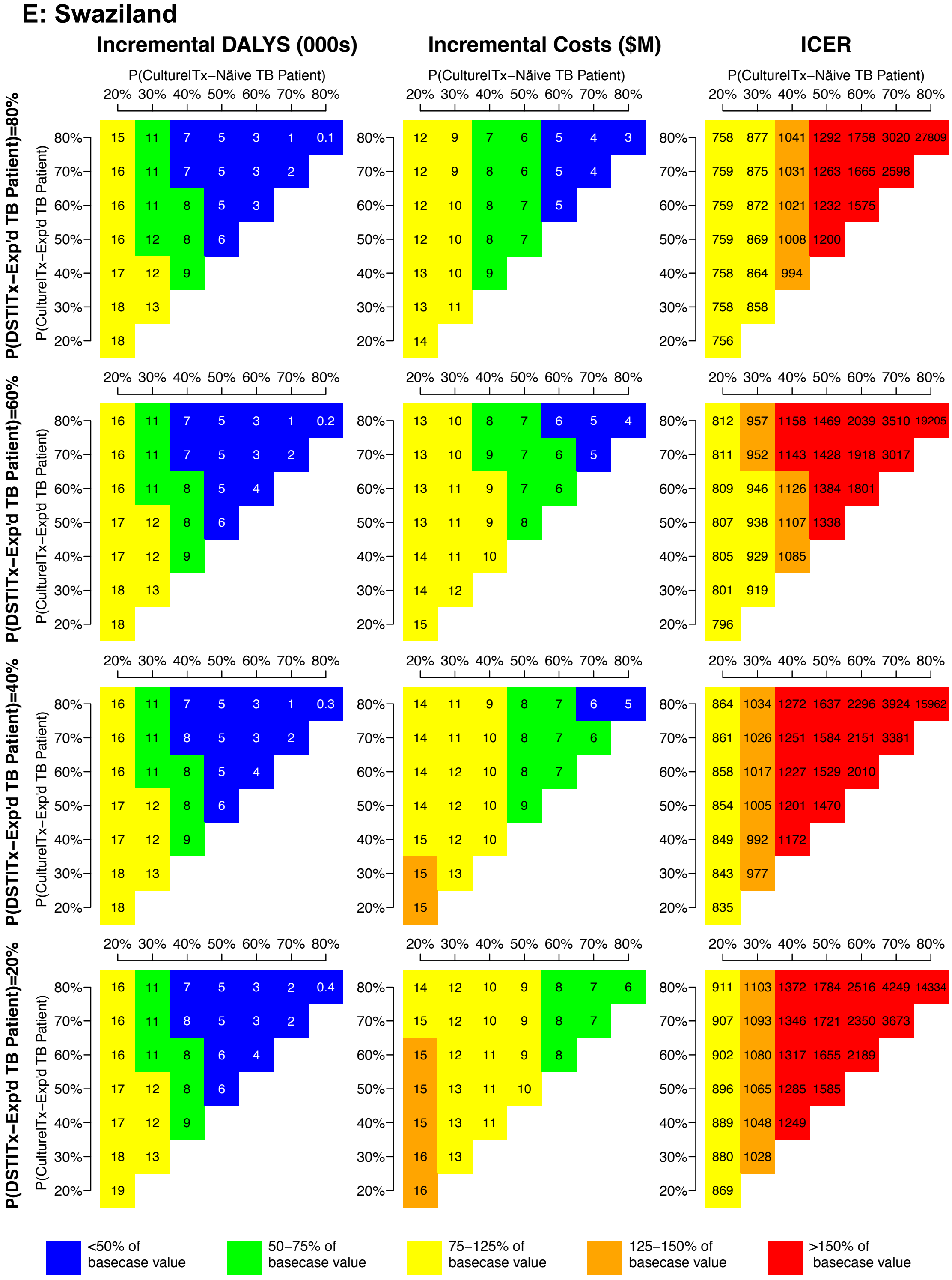

Figure S4 (continued): Three-way sensitivity analyses showing effects of changes in culture and DST coverage on major study outcomes, by country.

* Costs, DALYs and ICERs assessed over a 10-year analytic horizon with a US$30 Xpert unit cost. All other parameters held at their mean posterior values.

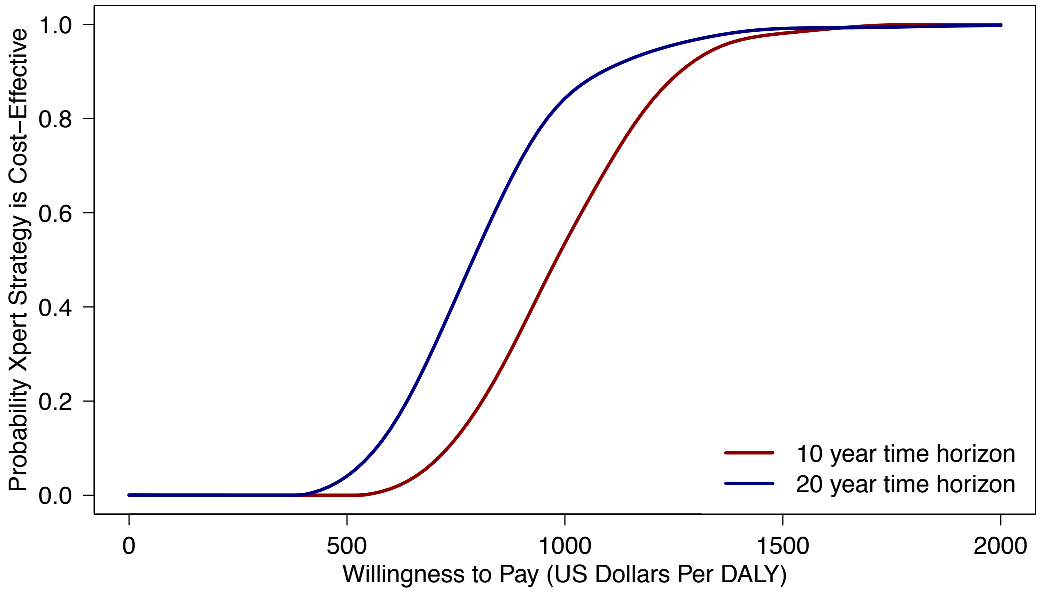

Figure S5: Cost-effectiveness acceptability curves showing probability that Xpert strategy is cost-effective as a function of willingness to pay for health benefits.
